# The mosses (Bryophyta) of Capitán Prat Province, Aisén Region, southern Chile

**DOI:** 10.3897/phytokeys.68.9181

**Published:** 2016-08-16

**Authors:** Juan Larraín

**Affiliations:** 1 Instituto de Biología, Facultad de Ciencias, Pontificia Universidad Católica de Valparaíso, Campus Curauma, Av. Universidad 330, Curauma, Valparaíso, Chile

**Keywords:** Biodiversity, checklist, range extensions, new records, southern South America

## Abstract

The bryophytes of Capitán Prat province have remained one of the least explored in Chile. The eventual construction of several dams on the rivers Baker and Pascua required prospection of all groups of organisms including bryophytes, work that was facilitated by the recent construction of vehicular roads that now offer easy access to previously almost unaccessible locations. The results of intense bryophyte collecting during the austral summer of 2007 are here presented. A total of 260 moss taxa are reported for the province, corresponding to 256 species and four infraspecific taxa, of which 211 are new records for the province, 54 are new for Aisén Region, and two are new records for continental Chile (*Pohlia longicollis* (Hedw.) Lindb. and Rigodium toxarion var. robustum (Broth.) Zomlefer). Twelve species extend their known distribution ranges to the north, whereas 49 extend them to the south.

## Introduction

The Aisén Region is divided into four administrative provinces, including Capitán Prat as the southernmost province of the Region, bordering by the south with Última Esperanza Province in the Magallanes Region. It is of particular botanical interest due to: (i) the diversity of climates, ranging from the per-humid temperate rainforests along the coast line and the western archipelago, to the extremely dry steppe in the eastern border with Argentina, and (ii) it is the southernmost ice-free area of continental Chile before the beginning of the massive Southern Patagonian Ice Field that partially isolates it from the neighboring Magallanes Region.

**Figure 1.**
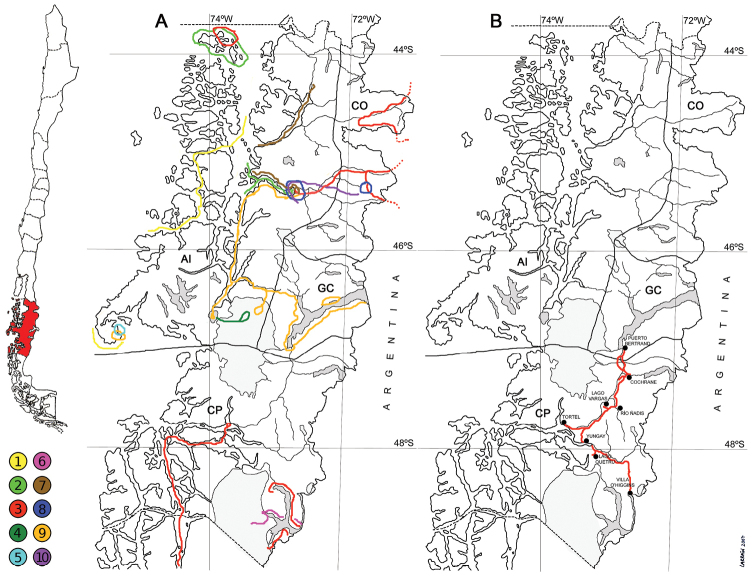
Map of Aisén Region showing the four provinces (**AI** = Aisén; **CO** = Coihaique; **GC** = General Carrera; **CP** = Capitán Prat) and the itinerary of bryological expeditions. **A** Expeditions that collected mosses previous to this study: 1. Charles Darwin, 1834; 2. Per Dusén, 1896–97; 3. Carl Skottsberg, 1908–09; 4. Federico Reichert and Cristóbal Hicken, 1921; 5. Heikii Roivainen, 1929; 6. Arturo Donat, 1933; 7. Gerhard Schwabe, 1939–41; 8. Rolf Santesson, 1940–41; 9. Tarow Seki, 1967; 10. Hironori Deguchi, 1981 **B** Explored area during the 2007 expedition where collections reported here were made. Inset: map of Chile showing in red the location of Aisén Region. (dotted line = regional border; continuous line = provincial limit).

Although the Aisén Region has received considerable exploration by bryologists, and there is even a moss flora already published (Seki 1974, published under the incorrect title of “Provincia de Aisén”), the southernmost province of the region has received very few visits by moss collectors (Fig. 1A). Surprisingly, to date, only two expeditions have collected mosses in the province, i.e., those by Carl Skottsberg at Canal Baker (Caleta Hale, Caleta Connor and Puerto Cueri-Cueri) in the austral winter of 1908, and at Lago O’Higgins in the summer of 1909 (Skottsberg 1916), and those by Arturo Donat at Lago O’Higgins on 1933, as part of the Argentinian expedition “Gaea” (Donat 1936a, 1936b). The first bryophytes reported for the province were 15 liverwort species (Stephani 1911), based on Skottsberg’s collections of 1908. The main scope of Skottsberg’s work was investigating vascular plants, yet Skottsberg (1916) reported 19 moss species, 38 liverworts, and some lichens. Skottsberg’s collections where further studied by Cardot and Brotherus (1923), who reported five new records for the localities mentioned above. Later, Donat reported 12 additional moss species (Donat 1936a), including six new to science (although one of them in the Argentinian side of Lago O’Higgins, locally named there Lago San Martín), and in a second work describing the flora of the western shore of O’Higgins lake (Donat 1936b), he added five more species, making a total of 40 moss species known for the province by 1936. In his moss flora of Aisén Region, Seki (1974) did not report a single locality that belonged to Capitán Prat Province. More recently, Bell et al. (2007) described a new genus of mosses, *Ombronesus* N.E.Bell, N.Pedersen & A.E.Newton, based on collections gathered by the Raleigh International expedition of 2003 in Katalalixar National Reserve, near Caleta Tortel. The floristic results of the latter expedition have not yet been published.

Unfortunately, in his checklist of Chilean mosses, Müller (2009a) reports most of the species listed by Cardot and Brotherus (1923) and all those reported by Donat (1936a) as belonging to Aisén Province, and did not mention the works by Skottsberg (1916) or by Donat (1936b). The result is that Müller (2009a) reports only seven taxa for Capitán Prat Province in his checklist. This partial information led Cuvertino et al. (2012) to report a new record for the province, *Distichium capillaceum* (Hedw.) Bruch & Schimp., although this taxon was already reported 100 years ago by Skottsberg (1916) from the NE shore of Lago O’Higgins. With the description of the new species *Ombronesus stuvensis* (Bell et al. 2007), *Ulota billbuckii* Garilleti, Mazimpaka & F.Lara and *Ulota streptodon* Garilleti, Mazimpaka & F.Lara (Garilleti et al. 2012), and *Ulota larrainii* Garilleti, Mazimpaka & F.Lara (Garilleti et al. 2015), and the new reports of *Lepyrodon patagonicus* (Cardot & Broth.) B.H.Allen (Allen 1999), *Brachythecium subpilosum* (Hook.f. & Wilson) A.Jaeger (Cuvertino et al. 2012), Hedwigia ciliata var. nivalis Hampe (Ellis et al. 2014), *Philonotis esquelensis* Matteri (Jimenez et al. 2014), and *Philonotis brevifolia* Herzog (Jimenez et al. 2016), the moss taxa known for the province rises to 49. This number is in contrast with the 250 moss taxa known from the neighboring Última Esperanza Province, or with the 311 taxa reported for Aisén Province (Müller 2009a).

## Methods

### Study site

**Figure 2.**
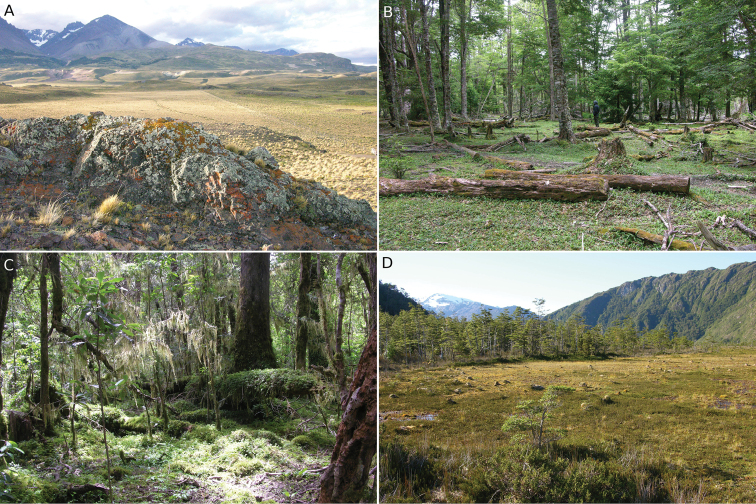
Different habitats of Capitán Prat. **A** Steppe at Estancia Chacabuco **B** *Nothofagus dombeyi–Nothofagus antarctica–Pilgerodendron uviferum* forest at Lago Vargas **C** *Nothofagus dombeyi–Drimys winteri–Podocarpus nubigenus* forest at Río Bravo **D** Peatland at Lago Leal.

Capitan Prat Province lies in the southern portion of Aisén Region (XI) in southern Chile, spanning between lat. 46°57'-49°09'S and long. 71°51'-75°36'W, with a total area of 37200 km². The province is crossed by three major river basins from north to south (the rivers Baker, Bravo and Pascua), with Baker river the largest river in Chile in terms of the volume of water. The province shows a remarkable heterogeneity of landscapes and climates (Fig. 2), ranging from extremely rainy areas in the western archipelago with rainfall measured up to 4266 mm a year at San Pedro station, to 191 mm in the steppe habitats in the easternmost areas, measured at Chile Chico, in adjacent General Carrera Province (di Castri and Hajek 1976), the closest available weather station in the easternmost side. Temperatures are mild, with a mean annual temperature measured at 8.2 °C at San Pedro and 11.5 °C at Chile Chico (di Castri and Hajek 1976). The geography is very rough, with snow-capped mountains dominating the landscape, Mt. San Lorenzo being the highest peak in the province, at 3706 m a.s.l. The province is flanked by the Northern and Southern Patagonian Ice Fields, and to the west it sinks into the Pacific Ocean in a large number of islands, fjords and channels, many of them still barely explored. It is one of the least populated provinces of Chile with a little more than 3000 people as measured by the 2012 census, and with more than twice the area of the whole Metropolitan Region of Chile, where more than 7 million people live.

In terms of the vegetation that dominates the landscape, there is a clearly marked gradient from east to west, mostly determined by the extreme variation in rainfall as explained above. In the easternmost parts of the province the vegetation fits into Gajardo’s (1984) concept of the “Patagonian Steppe of Aisén”, dominated by low shrubs and grasses, where the dominant species are *Baccharis patagonica* Hook. & Arn., *Stipa neaei* Nees ex Steud., *Festuca simpliciuscula* (Hack.) E.B.Alexeev, *Mulinum spinosum* Pers., *Adesmia longipes* Phil., and *Azorella incisa* Wedd., among others. This dry vegetation can be found in Estancia Chacabuco, around Lago Cochrane, and in Tamango National Reserve.

Descending in altitude, and towards the west, the vegetation is dominated by deciduous forests and shrublands, inserted in what Gajardo (1984) calls “Deciduous Forest fo Aisén”. This vegetation is very heterogeneous depending on the degree of disturbance of the land and in the local variations in soils, exposition, slopes and drainage. The dominant tree species are *Nothofagus pumilio* (Poepp. & Endl.) Krasser, *Nothofagus betuloides* (Mirb.) Oerst., *Nothofagus antarctica* (G.Forst.) Oerst., and *Embothrium coccineum* J.R.Forst. & G.Forst. In the drier places shrublands can be found, dominated mostly by *Gaultheria mucronata* Hook. & Arn., *Chiliotrichum diffusum* (G.Forst.) Kuntze, *Berberis microphylla* G.Forst. and *Escallonia serrata* Sm. In the areas with stronger grazing impact, and where the forest has been cut, fields of *Taraxacum officinale* F.H.Wigg., *Holcus lanatus* L. and *Dactylis glomerata* L. are common. The mosaic formed by these plant formations is frequent at mid altitude and is where most of the farming activity of the province is developed, mostly in the middle part of Río Baker.

Moving further towards the west, the vegetation changes into what Gajardo (1984) classifies as the region of “Evergreen Forests and Peatlands”. Several different kinds of forests coexist here, and there is a marked gradient too in its composition that relates with the available rainfall. The less humid forests are dominated by *Nothofagus dombeyi* (Mirb.) Oerst. and *Nothofagus nitida* (Phil.) Krasser, whereas the most humid are dominated by *Podocarpus nubigenus* Lindl., *Drimys winteri* J.R.Forst. & G.Forst., *Weinmannia trichosperma* Cav., *Raukaua laetevirens* (Gay) Frodin, and several Myrtaceae species. Areas with little drainage are dominated by *Pilgerodendron uviferum* (D.Don) Florin, *Tepualia stipularis* (Hook. & Arn.) Griseb., and *Sphagnum magellanicum* Brid. peatlands. Moving even further to the west the vegetation is dominated by cushion plants and shrubs like *Astelia pumila* (G.Forst.) Gaudich., *Donatia fascicularis* J.R.Forst. & G.Forst., *Oreobolus obtusangulus* Gaudich., *Empetrum rubrum* Vahl ex Willd. and *Lepidothamnus fonckii* Phil. (Gajardo 1984), which alternates with very humid evergreen forests in the ravines and protected places. This vegetation can be found along the coastline at Caleta Tortel, Puerto Yungay, and around Lago Quetru.

### Data collection

Two field trips were made during the Austral summer of 2007, making up a total of 13 full days of collecting. During these expeditions a total of 1283 bryophyte collections were made throughout the area between the localities of Puerto Bertrand and Villa O’Higgins, most of them adjacent to the main vehicular roads (Route 7, the road to Tortel, and the road to Villa O’Higgins) spanning the whole basin of Baker River and some sites along the Bravo and Pascua River basins (Fig. 1B), distributed in 56 collecting sites. Of this total, 1091 collections corresponded to mosses, with the remainder being liverworts and hornworts. Additionally, 74 recent moss collections from this same area kept at the Universidad de Concepción herbarium (CONC) were examined (Suppl. material 1).

The collecting localities were arbitrarily chosen, attempting to include the largest number of different floristic associations and landscapes, both in the driest and the wettest areas of the province and throughout all the gradients in between, from the coast line to about 700 m a.s.l.

The specimens were identified with the monographs or taxonomic revisions currently available for each group, and comparing with reference material kept at CONC, NY, and F herbaria. Additionally, many colleagues helped with the identification of difficult material, or for groups where there are no revisions available: María Jesús Cano and Mayte Gallego (Pottiaceae), Ricardo Garilleti (*Ulota*), Paco Lara (*Orthotrichum*), Lars Hedenäs (Amblystegiaceae), John Spence (*Bryum*), Barbara Murray (*Andreaea*), Guillermo Suárez (*Pohlia*), Soledad Jimenez (*Philonotis*) and Barbara Andreas (*Blindia*). All vouchers are deposited in the Universidad de Concepción herbarium (CONC).

The species list is given below in alphabetical order, indicating the new records for Chile (***), for Aisén Region (**) and for Capitán Prat Province (*). A systematic arrangement of the taxa, including habitat information, altitude where taxa were found, frequency in the studied area, global and Chilean distribution, and a list of studied specimens, is presented in the Suppl. material 1.

For the analysis of the distribution patterns, the many different patterns observed were reduced into five major categories for simplification: (1) Wide distribution (WD), meaning cosmopolitan or subcosmopolitan species present in both hemispheres and in both tropical and temperate areas of the planet; (2) Bipolar, meaning species distributed in the temperate areas of both hemispheres with eventual isolated populations at high altitudes in the tropics; (3) Austral, meaning species with various distribution patterns along the Southern Hemisphere - this is the most heterogeneous group because it includes both species with very wide circumsubantarctic distributions to narrow “sub-endemics” maybe recorded from a few populations in southern South America and South Africa, for example; (4) Andean, meaning species mostly distributed throughout the Andes but incidentally extending into Antarctica, Africa or some subantarctic Islands; and (5) Endemic, meaning species only known from southern South America and eventually extending into Juan Fernández or the Falkland and South Georgia Islands.

## Results

A total of 260 moss taxa belonging to 256 species and 4 infraspecific taxa, in 42 botanical families are reported for Capitán Prat Province (Suppl. material 1). The most diverse families were the Orthotrichaceae with 33 taxa, the Pottiaceae with 24 and the Bryaceae with 20 taxa. The most species rich genera were *Bryum* with 20 species, *Ulota* with 15 and *Syntrichia* with 11 species. Two taxa are reported for continental Chile for the first time, 54 for Aisén Region, and 211 are for the first time reported for Capitán Prat Province, with 12 taxa extending their known distribution limits to the north, and 49 to the south.

*Achrophyllum anomalum (Schwägr.) H.Rob. var. anomalum

Achrophyllum anomalum var. pallidum (Cardot & Broth.) S.He

*Achrophyllum haesselianum (Matteri) Matteri

**Figure 3.**
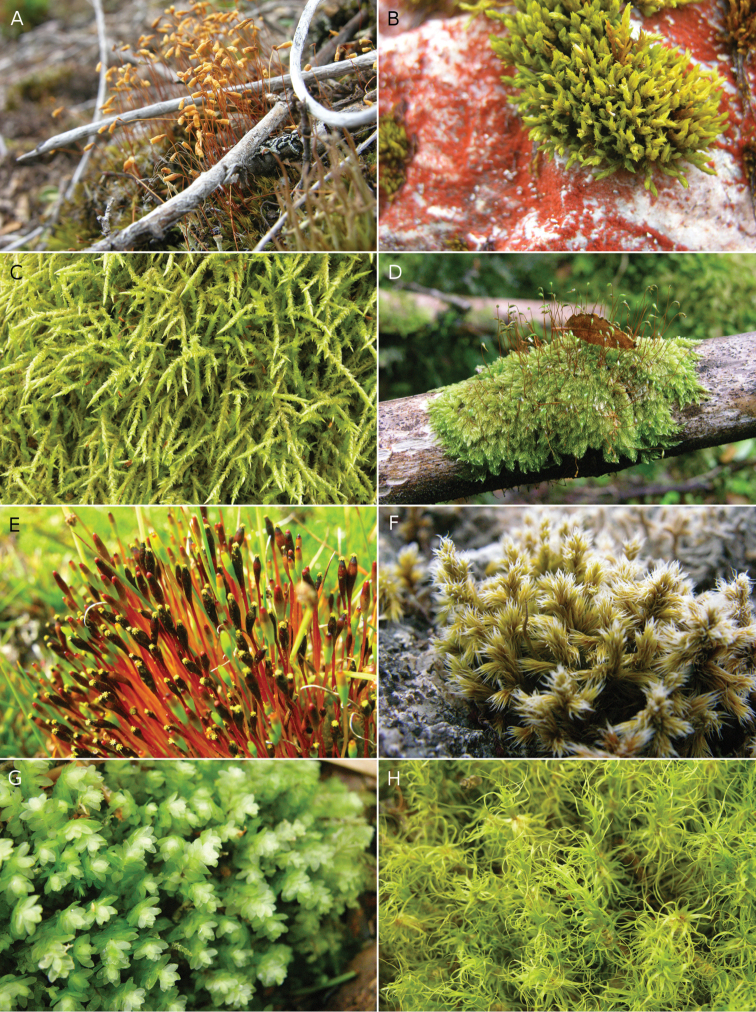
Mosses of Capitán Prat Province. **A** *Pohlia nutans* **B** *Racomitrium didymum* **C** *Acrocladium auriculatum* **D** *Rhaphidorrhynchium callidum* **E** *Tetraplodon fuegianus* **F** *Racomitrium geronticum* **G** *Achrophyllum magellanicum* **H** *Platyneuron praealtum*.

*Achrophyllum magellanicum (Besch.) Matteri (Figure 3G)

*Acrocladium auriculatum* (Mont.) Mitt. (Figure 3C)

**Acroschisma wilsonii (Hook.f.) A.Jaeger

*Amphidium tortuosum (Hornsch.) Cufod.

*Ancistrodes genuflexa (Müll.Hal.) Crosby

*Andreaea alpina* Hedw.

*Andreaea fuegiana* (Cardot) S.W.Greene

*Andreaea regularis* Müll.Hal.

*Andreaea vaginalis* Herzog

*Arbusculohypopterygium arbuscula* (Brid.) M.Stech, T.Pfeiff. & W.Frey

*Bartramia ithyphylloides Schimp. ex Müll.Hal.

*Bartramia mossmaniana* Müll.Hal.

*Bartramia patens* Brid.

*Bartramia robusta Hook.f. & Wilson

*Bartramia stricta Brid.

*Blindia contecta (Hook.f. & Wilson) Müll.Hal.

*Blindia magellanica* Schimp.

*Brachytheciastrum paradoxum* (Hook.f. & Wilson) Ignatov & Huttunen

*Brachythecium albicans (Hedw.) Schimp.

*Brachythecium austroglareosum (Müll.Hal.) Paris

*Brachythecium austrosalebrosum (Müll.Hal.) Paris

*Brachythecium subpilosum* (Hook.f. & Wilson) A.Jaeger

*Brachythecium subplicatum (Hampe) A.Jaeger

*Breutelia angustiretis E.B.Bartram

*Breutelia aureola (Besch. ex Müll.Hal.) Besch.

*Breutelia dumosa Mitt.

*Breutelia integrifolia* (Taylor) A.Jaeger

*Breutelia plicata Mitt.

*Breutelia subplicata Broth.

**Bryum algovicum Sendtn. ex Müll.Hal.

**Bryum archangelicum Bruch & Schimp.

*Bryum australe Hampe

**Figure 4.**
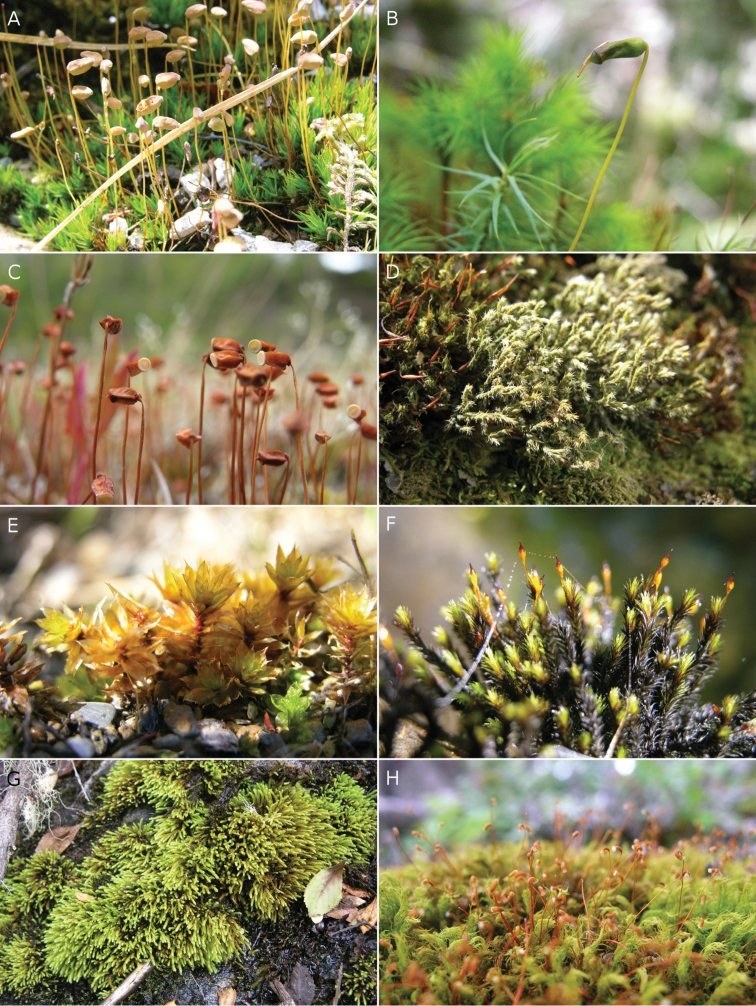
Mosses of Capitán Prat Province. **A** *Notoligotrichum minumum* **B** *Polytrichadelphus magellanicus* **C** *Polytrichum piliferum* **D** Hedwigia ciliata var. nivalis **E** *Bryum billarderi* **F** *Racomitrium lamprocarpum* **G** *Pohlia wahlenbergii* **H** *Hymenodontopsis mnioides*.

**Bryum billarderi Schwägr. (Figure 4E)

*Bryum caespiticium Hedw.

**Bryum canariense Brid.

*Bryum capillare Hedw.

*Bryum clavatum* (Schimp.) Müll.Hal.

*Bryum coronatum Schwägr.

*Bryum crassinervium* Lorentz

**Bryum dichotomum Hedw.

*Bryum donatii* Thér.

*Bryum gemmatum* Müll.Hal.

*Bryum laevigatum Hook.f. & Wilson

*Bryum macrophyllum Cardot & Broth.

*Bryum perlimbatum Cardot

**Bryum platyphyllum (Schwägr.) Müll.Hal.

*Bryum pseudotriquetrum* (Hedw.) Schwägr.

**Bryum puconense Herzog & Thér.

*Bryum subapiculatum Hampe

*Calyptopogon mnioides (Schwägr.) Broth.

*Camptodontium cryptodon (Mont.) Reimers

**Campylium stellatum (Hedw.) C.E.O.Jensen

*Campylopodium euchlorum (Mont.) Matteri

*Campylopodium medium (Duby) Giese & J.-P.Frahm

*Campylopus acuminatus Mitt.

*Campylopus clavatus (R.Br.) Wilson

*Campylopus incrassatus Müll.Hal.

*Campylopus introflexus (Hedw.) Brid.

*Campylopus purpureocaulis* Dusén

*Campylopus pyriformis (Schultz) Brid.

*Campylopus vesticaulis Mitt.

*Catagonium nitens (Brid.) Cardot

*Ceratodon purpureus (Hedw.) Brid. subsp. purpureus

*Ceratodon purpureus subsp. convolutus (Reichardt) Burley

*Chorisodontium aciphyllum* (Hook.f. & Wilson) Broth.

*Chorisodontium dicranellatum (Dusén) Roiv.

*Chorisodontium spegazzinii (Müll.Hal.) Roiv.

**Chrysoblastella chilensis (Mont.) Reimers

*Conostomum pentastichum (Brid.) Lindb.

Cratoneuropsis relaxa (Hook.f. & Wilson) M.Fleisch. ex Broth. subsp. minor (Hook.f. & Wilson) Ochyra

*Cryphaea consimilis Mont.

*Cryphaeophilum molle (Dusén) M.Fleisch.

*Daltonia gracilis Mitt.

*Daltonia trachyodonta Mitt.

*Dendroligotrichum dendroides (Hedw.) Brid.

*Dendroligotrichum squamosum* (Hook.f. & Wilson) Broth. ex Cardot

*Dicranella campylophylla (Taylor) A.Jaeger

*Dicranella hookeri (Müll.Hal.) Cardot

**Dicranella pseudorufescens Cardot & Broth.

*Dicranoloma billardieri* (Brid.) Paris

*Dicranoloma chilense (De Not.) Ochyra & Matteri

**Dicranoloma imponens (Mont.) Renauld

*Dicranoloma menziesii (Hook.f. & Wilson) Paris

*Dicranoloma perremotifolium (Dusén) Broth.

*Dicranoloma robustum* (Hook.f. & Wilson) Paris

*Didymodon andreaeoides Cardot & Broth.

*Didymodon australasiae (Hook. & Grev.) R.H.Zander

*Didymodon fuscus (Müll.Hal.) J.A.Jiménez & M.J.Cano

*Distichium capillaceum* (Hedw.) Bruch & Schimp.

*Distichophyllum dicksonii (Hook. & Grev.) Mitt.

*Ditrichum cylindricarpum (Müll.Hal.) F.Muell.

*Ditrichum difficile (Duby) M.Fleisch.

*Ditrichum ditrichoideum (Cardot) Ochyra

*Ditrichum heteromallum (Hedw.) E.Britton

**Drepanocladus aduncus (Hedw.) Warnst.

*Drepanocladus longifolius (Mitt.) Broth. ex Paris

*Drepanocladus polygamus (Schimp.) Hedenäs

*Dryptodon austrofunalis (Müll.Hal.) Ochyra & Żarnowiec

*Dryptodon humilis (Mitt.) Ochyra & Żarnowiec

*Dryptodon navicularis (Herzog) Ochyra & Żarnowiec

*Dryptodon trichophyllus* (Grev.) Brid.

*Encalypta ciliata Hedw.

**Encalypta rhaptocarpa Schwägr.

*Eriodon conostomus Mont.

*Eucamptodon perichaetialis (Mont.) Mont.

*Eurhynchiella acanthophylla (Mont.) M.Fleisch.

*Eustichia longirostris (Brid.) Brid

**Fabronia jamesonii Taylor

*Fissidens curvatus Hornsch.

*Fissidens rigidulus Hook.f. & Wilson

*Funaria hygrometrica Hedw.

*Glyphothecium sciuroides (Hook.) Hampe

*Gymnostomum calcareum Nees & Hornsch.

**Hebantia rigida (Lorentz) G.L.Merr.

Hedwigia ciliata Hedw. var. nivalis Hampe (Figure 4D)

**Hennediella antarctica (Ångström) Ochyra & Matteri

*Hennediella arenae (Besch.) R.H.Zander

**Hennediella heimii (Hedw.) R.H.Zander

*Hymenodontopsis mnioides (Hook.) N.E.Bell, A.E.Newton & D.Quandt (Figure 4H)

*Hymenoloma crispulum* (Hedw.) Ochyra

*Hymenoloma turpe* (Cardot) Cardot & Broth.

*Hymenostylium recurvirostrum (Hedw.) Dixon

*Hypnodendron microstictum Mitt. ex A.Jaeger & Sauerb.

*Hypnum chrysogaster Müll.Hal.

*Hypnum cupressiforme Hedw. var. cupressiforme

**Hypnum cupressiforme var. filiforme Brid.

*Hypnum cupressiforme var. mossmanianum (Müll.Hal.) Ando

*Hypnum skottsbergii Ando

*Hypopterygium didictyon Müll.Hal.

*Kiaeria pumila (Mitt.) Ochyra

*Leptobryum pyriforme (Hedw.) Wilson

**Leptodontium longicaule Mitt. var. microruncinatum (Dusén) R.H.Zander

**Leptostomum menziesii R.Br.

*Leptotheca gaudichaudii* Schwäger.

*Lepyrodon hexastichus (Mont.) Wijk & Margad.

*Lepyrodon lagurus* (Hook.) Mitt.

*Lepyrodon patagonicus* (Cardot & Broth.) B.H.Allen

*Lepyrodon tomentosus (Hook.) Mitt.

**Looseria orbiculata (Thér.) D.Quandt, Huttunen, Tangney & M.Stech

*Lopidium concinnum (Hook.) Wilson

**Macromitrium pertriste Müll.Hal.

**Mahua enervis W.R.Buck

*Matteria gracillima (Besch.) Goffinet

*Matteria papillosula (Thér.) Goffinet

*Neckera scabridens Müll.Hal.

*Notoligotrichum minimum (Cardot) G.L.Sm. (Figure 4A)

*Notoligotrichum trichodon (Hook.f. & Wilson) G.L.Sm.

*Oligotrichum austroaligerum G.L.Sm.

*Ombronesus stuvensis* N.E.Bell, N.Pedersen & A.E.Newton

**Orthodontium lineare Schwägr.

*Orthotrichum assimile Müll.Hal.

*Orthotrichum brotheri Dusén ex Lewinsky

*Orthotrichum elegantulum Schimp. ex Mitt.

**Orthotrichum hortense Bosw.

*Orthotrichum incanum Müll.Hal.

*Orthotrichum ludificans Lewinsky

**Orthotrichum pariatum Mitt.

*Orthotrichum rupestre Schleich. ex Schwägr.

*Pararhacocarpus patagonicus (Broth.) J.-P.Frahm

*Philonotis brevifolia* Herzog

*Philonotis esquelensis* Matteri

*Philonotis krausei (Müll.Hal.) Broth.

**Philonotis polymorpha (Müll.Hal.) Kindb.

**Philonotis scabrifolia (Hook.f. & Wilson) Braithw.

*Philonotis vagans* (Hook.f. & Wilson) Mitt.

**Pilopogon schilleri Herzog & Thér.

**Plagiothecium lucidum (Hook.f. & Wilson) Paris

*Platyneuron praealtum (Mitt.) Ochyra & Bednarek-Ochyra (Figure 3H)

*Pohlia cruda* (Hedw.) Lindb.

***Pohlia longicollis (Hedw.) Lindb.

*Pohlia nutans (Hedw.) Lindb. (Figure 3A)

*Pohlia wahlenbergii (F.Weber & D.Mohr) A.L.Andrews (Figure 4G)

*Polytrichadelphus magellanicus (Hedw.) Mitt. (Figure 4B)

**Polytrichastrum alpinum (Hedw.) G.L.Sm.

*Polytrichastrum longisetum (Sw. ex Brid.) G.L.Sm.

*Polytrichum juniperinum Hedw.

*Polytrichum piliferum* Hedw. (Figure 4C)

**Polytrichum strictum Menzies ex Brid.

*Porotrichum arbusculans (Müll.Hal.) Ochyra

*Pseudocrossidium crinitum (Schultz) R.H.Zander

*Ptychomniella ptychocarpon* (Schwägr.) W.R.Buck, C.J.Cox, A.J.Shaw & Goffinet

*Ptychomnion cygnisetum (Müll.Hal.) Kindb.

*Racomitrium didymum (Mont.) Lorentz (Figure 3B)

*Racomitrium geronticum* Müll.Hal. (Figure 3F)

*Racomitrium heterostichoides Cardot

*Racomitrium laevigatum* A.Jaeger

*Racomitrium lamprocarpum (Müll.Hal.) A.Jaeger (Figure 4F)

*Racomitrium orthotrichaceum (Müll.Hal.) Paris

*Racomitrium pachydictyon Cardot

*Racomitrium rupestre (Hook.f. & Wilson) Wilson & Hook.f.

*Racomitrium subcrispipilum (Müll.Hal.) A.Jaeger

*Rhacocarpus purpurascens (Brid.) Paris

**Rhaphidorrhynchium amoenum (Hedw.) M.Fleisch.

*Rhaphidorrhynchium callidum (Mont.) Broth. (Figure 3D)

*Rhaphidorrhynchium dendroligotrichum (Dusén) Broth.

*Rigodium adpressum Zomlefer

*Rigodium brachypodium (Müll.Hal.) Paris

*Rigodium pseudothuidium Dusén

*Rigodium tamarix Müll.Hal.

*Rigodium toxarion (Schwägr.) A.Jaeger var. toxarion

***Rigodium toxarion var. robustum (Broth.) Zomlefer

*Sanionia uncinata (Hedw.) Loeske

*Sauloma tenella (Hook.f. & Wilson) Mitt.

*Schistidium andinum* (Mitt.) Herzog

**Sciuro plumosum (Hedw.) Ignatov & Huttunen

**Scorpidium revolvens (Sw.) Rubers

*Scouleria patagonica* (Mitt.) A.Jaeger

*Sematophyllum scorpiurus (Mont.) Mitt.

*Sphagnum falcatulum Besch.

*Sphagnum fimbriatum* Wilson

*Sphagnum magellanicum* Brid.

*Straminergon stramineum* (Dicks. ex Brid.) Hedenäs

*Syntrichia anderssonii* (Ångström) R.H.Zander

**Syntrichia costesii (Thér.) R.H.Zander

**Syntrichia epilosa (Broth. ex Dusén) R.H.Zander

**Syntrichia fragilis (Taylor) Ochyra

**Syntrichia glacialis (Kunze ex Müll.Hal.) R.H.Zander

*Syntrichia lithophila (Dusén) Ochyra & R.H.Zander

*Syntrichia magellanica (Mont.) R.H.Zander

*Syntrichia princeps (De Not.) Mitt.

*Syntrichia pseudorobusta (Dusén) R.H.Zander

**Syntrichia ruralis (Hedw.) F.Weber & D.Mohr

**Syntrichia scabrella (Dusén) R.H.Zander

*Tayloria dubyi Broth.

**Tayloria magellanica (Brid.) Mitt.

**Tayloria stenophysata (Herzog) A.K.Kop.

*Tetraplodon fuegianus Besch. (Figure 3E)

**Tortella knightii (Mitt.) Broth.

**Tortula atrovirens (Sm.) Lindb.

*Ulota billbuckii* Garilleti, Mazimpaka & F.Lara

**Ulota carinata Mitt.

*Ulota fuegiana Mitt.

*Ulota germana (Mont.) Mitt.

*Ulota larrainii* Garilleti, Mazimpaka & F.Lara

*Ulota luteola (Hook.f. & Wilson) Wijk & Marg.

**Ulota macrocalycina Mitt.

*Ulota macrodontia Dixon & Malta

*Ulota magellanica (Mont.) A.Jaeger

**Ulota phyllantha Brid.

**Ulota pusilla Malta

**Ulota pycnophylla Dusén ex Malta

*Ulota pygmaeothecia (Müll.Hal.) Kindb.

*Ulota rufula (Mitt.) A.Jaeger

*Ulota streptodon* Garilleti, Mazimpaka & F.Lara

*Vittia pachyloma (Mont.) Ochyra

*Warnstorfia exannulata (Schimp.) Loeske

**Warnstorfia fluitans (Hedw.) Loeske

*Warnstorfia fontinialopsis (Müll.Hal.) Ochyra

*Weymouthia cochlearifolia (Schwägr.) Dixon

*Weymouthia mollis (Hedw.) Broth.

**Zygodon hookeri Hampe var. hookeri

*Zygodon hookeri var. leptobolax (Müll.Hal.) Calabrese

*Zygodon magellanicus Dusén ex Malta

*Zygodon papillatus Mont.

*Zygodon pentastichus (Mont.) Müll.Hal.

*Zygodon pichinchensis (Taylor) Mitt.

*Zygodon reinwardtii* (Hornsch.) A.Braun

From the analysis of the five simplified distribution patterns, 38.08% (n=99) of the taxa are actually endemics of southern South America, followed by the “austral” taxa with 30.77% (n=80). The rest of the taxa were found to be either of “wide distribution” (13.85%, n=36), “bipolar” (9.62%, n=25), or “Andean” (7.69%, n=20).

## Discussion

The known diversity of Capitán Prat Province was incremented from 49 to 260 taxa, representing a 531% increment. This is remarkable and demonstrates the very little attention this area has historically received in terms of its bryophyte diversity. The current number of species reported for the province makes sense with the numbers known from the adjacent provinces of Aisén (311) and Última Esperanza (250). The number of moss taxa known from adjacent General Carrera province [27 in Müller’s (2009a) checklist] is still very low and certainly needs further study, although General Carrera province lacks the most humid habitats found in the coastal lowland rainforests, that in this study yielded a large number of taxa not found anywhere else.

Three taxa were described as new based on the collections gathered during this study in Capitán Prat (*Ulota billbuckii*, *Ulota larraini* and *Ulota streptodon*), and four others were found to be new records for Chile (Hedwigia ciliata var. nivalis, *Philonotis esquelensis*, *Pohlia longicollis* and Rigodium toxarion var. robustum), the latter previously known to be an endemic of the Juan Fernández Islands. The number of new species and new records for Chile might increment even more after study of the material that still remains unidentified.

The high level of endemic taxa in southern South America temperate rainforests has already been reported in the literature for neighboring areas (i.e., Seki 1974, Villagrán et al. 2003, Larraín 2005). This is also the principal distribution pattern in Capitán Prat Province, with almost 40% of all the taxa known for the area being endemics. It is also interesting that the moss flora of these Austral forests is way more related to distant Austral lands as New Zealand, Australia, or some remote subantarctic islands rather than to neighboring Neotropical countries like Perú or Bolivia: only 7.69% of the taxa studied has a continuous distribution along the Andes into the northern Tropics. This is explained by the condition of “biogeographic island” of southern Chile forests, isolated from the rest of the continent by the very high peaks of central and northern Chile, and extremely arid deserts both to the north (Atacama) and to the east (Patagonian steppe).

Several species were found farther south from their previously known distribution ranges. This is interesting as it would appear to suggest that many of these taxa are apparently not able to disperse south of the Southern Patagonia Ice Field. This ice field would act as a natural barrier to the dispersion of Valdivian rainforest endemics such as *Ancistrodes genuflexa*, *Cryphaea consimilis*, *Cryphaeophilum molle*, *Eriodon conostomus*, *Eurhynchiella acanthophylla*, *Rhaphidorrhynchium dendroligotrichum*, *Rigodium tamarix*, and *Weymouthia cochlearifolia*, among others, preventing their colonization of Magallanes Region. This might be due also to less collecting effort in the more wet areas of Magallanes, because of the logistical difficulties to access the most western islands, where rainfall is similar to what can be found in the wettest areas of Capitán Prat.

It is important to mention that not a single locality above 700 m was visited. Although most of the area above this altitude is covered in ice and snow all year round, it would be interesting to visit higher altitude spots and glaciers, since several of the species found by Donat around the glaciers of Lago O’Higgins were not found during this expedition. Covering these sites would maybe increase even more the number of moss taxa known from Capitán Prat Province.

## Supplementary materials

Supplementary material 1 Systematic list of taxa and additional information Data type: specimens data This dataset is made available under the Open Database License (http://opendatacommons.org/licenses/odbl/1.0/). The Open Database License (ODbL) is a license agreement intended to allow users to freely share, modify, and use this Dataset while maintaining this same freedom for others, provided that the original source and author(s) are credited.Juan Larraín
