# Supplementary material for: The mosses (Bryophyta) of Capitán Prat Province, Aisén Region, southern Chile
Source: PhytoKeys. 2016 Aug 16;(68):91–116. doi: 10.3897/phytokeys.68.9181 (PMC5029133; doi:10.3897/phytokeys.68.9181)
Supplement: Supplementary material 1 — Systematic list of taxa and additional information [file phytokeys-068-091-s001.pdf]

Supplementary material 1: systematic list of taxa and additional information. The systematic arrangement of taxa follows Stech and Frey (2010). When there are alternative names for a taxon that have been widely used in the literature, they are indicated below the currently accepted name. For each taxon, habitat details of the observed vouchers are indicated, as well as the altitude they were collected, followed by a comment on how frequent the taxon was in the area; these comments are based in the number of collections available for each taxon and/or how frequent and abundant they were as observed in the field. Both the global and the Chilean distribution are given for each taxon. In the lists of the studied exsiccata, the name initials of the collectors are indicated (JL=Juan Larraín, AJ=Alejandra Jiménez, AM=Alicia Marticorena, RR= Roberto Rodríguez, ET=Ernesto Teneb, CS= Carl Skottsberg, AD=Arturo Donat).

Subdivision Sphagnophytina Doweld
Class Sphagnopsida Schimp.
Order Sphagnales Limpr.
Family Sphagnaceae Dumort.

Sphagnum falcatulum Besch.
Floating or submerged in puddles in *S. magellanicum* dominated peatlands, in swampy forest edges, or in draining ridges next to roads; alt. 20-380 m a.s.l.; rare in the studied area.
Distribution: an austral taxon growing in New Zealand, Tasmania, Macquarie Island, and southern South America (Karlin et al. 2013); reported in Chile from Malleco Province to Cape Horn (Müller 2009a).
Exsiccata: cuesta mirador Baker (JL 26507), Puerto Yungay (JL 26520), road south of Lago Quetru (JL 27765), Río Baker between Río Ñadis and Río Colonia (RR & ET 81).
Notes: new to Capitán Prat.

Sphagnum fimbriatum Wilson
On *Nothofagus-Pilgerodendron* or *Nothofagus-Drimys-Podocarpus* forest floor, or in forest edges next to roads, in humid to temporally flooded ground, sometimes in puddles in *Donatia-Astelia-Lepidothamnus* tundras, or in swampy *Escallonia* shrublands; alt. 20-90 m a.s.l.; common in the studied area.
Distribution: a widely distributed taxon in the Northern Hemisphere, also in New Zealand, South Africa, the Falkland Is., and southern South America (Crum 1984); in Chile known from Arauco Province to Cape Horn (Müller 2009a).
Exsiccata: road front of Lago Quetru (JL 27825), pasarela Lago Vargas (JL 26813, JL 26839), Caleta Tortel (JL 26879), Río Bravo (JL 26974, JL 26980, JL 26984), Route 7 south of Río Carrera (JL 26456), Route 7 north of Vagabundo (JL 26465).
Notes: previously reported by Donat (1936b) from the west side of O'Higgins Lake (in text, no voucher indicated).

Sphagnum magellanicum Brid.
This is the major peat-forming taxon in the area, although *Sphagnum* peatlands are not very abundant in Capitán Prat Province compared with adjacent areas; it can be found also on the ground in open *Pilgerodendron* forest, among *Donatia-Astelia-Lepidothamnus* tundras, or even in open slopes colonizing road cuts or clearcutted forest; alt 30-200 m a.s.l.; common in the studied area.
Distribution: widely distributed in temperate areas of the Northern Hemisphere, growing also in montane areas in the Neotropics, and in southern South America and the Falkland Is. (Crum 1984, Karlin et al. 2008); in Chile known from Arauco Province to Cape Horn (Müller 2009a).
Exsiccata: Caleta Tortel (JL 26906), Río Bravo (JL 26991), road to Villa O'Higgins (JL 27050), Lago

Leal (JL 27137), Route 7 north of Vagabundo (JL 26464 p.p. + *Riccardia fuscobrunnea*), Río Baker between Río Ñadis and Río Colonia (RR & ET 80, RR & ET 82), pasarela Lago Vargas (AM 1019a + *Polytrichastrum longisetum*).
Notes: previously reported by Skottsberg (1916) from Puerto Cueri-Cueri, Canal Baker (CS 73).

Subdivision Bryophytina Engler
Class Andreaeopsida J.H.Schaffn.
Order Andreaeales Limpr.
Family Andreaeaceae Dumort.

Acroschisma wilsonii (Hook.f.) A.Jaeger
On rocks in open places, ocasionally next to roads or rivers, or in open *Escallonia* shrublands; alt. 50-250 m a.s.l.; common in the studied area.
Distribution: an Andean taxon found between Colombia and the southernmost tip of South America (Gradstein et al. 2001), reported in Chile from Valdivia Province to Cape Horn (Müller 2009a).
Exsiccata: Lago Leal (JL 27810), road to Villa O'Higgins (JL 27054), road between Cochrane and Tortel, near río Carrera bridge (JL 26446B, JL 26448), in Tortel/Yungay fork, by the river (JL 26486), Lago Vargas (AM 1016), cruce Vagabundo (AM 1031).
Notes: new to Capitán Prat and to Aisén Region.

Andreaea alpina Hedw.
On rocks in open tundra environments; alt. 90-450 m a.s.l.; common in the studied area.
Distribution: widely distributed in the Southern Hemisphere, reported from Tasmania, New Zealand, almost every subantarctic island, southern South America, also in NW Europe and Greenland (Murray 2006); in Chile known from Ñuble Province to Cape Horn, and in Juan Fernández (Müller 2009a).
Exsiccata: Caleta Tortel (JL 26889, JL 26900, JL 26902), road to Villa O'Higgins (JL 27066, JL 27068, JL 27071, JL 27076).
Notes: previously reported by Donat (1936a) as *Andreaea squarrosa* Mitt. from west side of Lago O'Higgins (AD s.n.).

Andreaea fuegiana (Cardot) S.W.Greene
≡*Neuroloma fuegianum* Cardot
This species grows on seeping rocks, generally next to glaciers, forming small cushions; very rare in the studied area, but eventually more common around glaciers.
Distribution: endemic to southern South America, distributed in Chile from Aisén Province to Tierra del Fuego (Müller 2009a).
Notes: previously reported by Donat (1936a) as *Neuroloma fuegianum* Cardot from the west side of Lago O'Higgins (AD s.n.).

Andreaea regularis Müll.Hal.
On rocks; very rare in the studied area, but eventually more common around glaciers.
Distribution: native from southern South America and Antarctica (Ochyra et al. 2008), distributed in Chile from Malleco Province to Cape Horn (Müller 2009a).
Notes: previously reported by Donat (1936a) as *Andreaea verruculosa* Cardot from west side of Lago O'Higgins (no voucher indicated).

Andreaea vaginalis Herzog
Notes: insufficiently known taxon, at present endemic to Capitán Prat Province, incorrectly reported to Aisén Province by Müller (2009a). First described by Herzog from Donat collections at glaciers of

Lago O'Higgins at 1,600-1,800 m a.s.l. (Donat 1936a) from the west side of Lago O'Higgins (AD 14, AD 15). Known only from the type material housed at JE (JE04000683 and JE04000682) with duplicates in PC (PC0657737 and PC0657738), labeled as collected at 1,200 m a.s.l. (AD 15) and 1,500 m a.s.l. (AD 14).

Class Polytrichopsida Doweld
Order Polytrichales M.Fleisch.
Family Polytrichaceae Schwägr.

Dendroligotrichum dendroides (Hedw.) Brid.
The largest moss in Chile, a forest floor species reaching up to 60 cm tall in favorable conditions, in *Nothofagus dombeyi-Podocarpus nubigena-Drimys winteri* or *Nothofagus-Embothrium-Amomyrtus* forests; alt. 30-130 m a.s.l.; common in the studied area.
Distribution: endemic to southern South America (Stech et al. 2008), distributed in Chile from Arauco Province to Cape Horn (Müller 2009a).
Exsiccata: Lago Leal (JL 27813), Lago Quetru (JL 27771, JL 27847, JL 27846 p.p. + *Rhaphidorrhynchium dendroligotrichum* + *Distichophyllum dicksonii*), Route 7 south of Cochrane km 90 (JL 27921), Río Bravo (JL 27040B), road to Tortel (JL 27906 p.p. + *Rhaphidorrhynchium dendroligotrichum*, JL 27910 p.p. + *Rhaphidorrhynchium dendroligotrichum*, JL 26997 p.p. + *Rhaphidorrhynchium dendroligotrichum*).
Notes: new to Capitán Prat. This taxon has been considered also distributed in New Zealand (e.g. Schiavone 1993), but recent molecular studies support the recognition of the New Zealand species as a separate taxon (Stech et al. 2008).

Dendroligotrichum squamosum (Hook.f. & Wilson) Broth. ex Cardot
On the floor of *Nothofagus dombeyi-Nothofagus antarctica-Pilgerodendron uviferum* or *Nothofagus-Podocarpus-Drimys* forests; alt. 20-130 m a.s.l.; not common in the studied area.
Distribution: endemic to southern South America, extending to the Falkland Islands and South Georgia (Schiavone 1993), distributed in Chile from Valdivia Province to Cape Horn (Müller 2009a).
Exsiccata: Río Bravo (JL 27025), Lago Vargas (JL 26802).
Notes: previously reported by Donat (1936a) from west side of Lago O'Higgins (in text, no voucher indicated).

Hebantia rigida (Lorentz) G.L.Merr.
On stony soil next to vehicular road by *Drimys-Podocarpus-Amomyrtus* forest; alt. 50 m a.s.l.; extremely rare in the studied area.
Distribution: endemic to Chile (Ireland et al. 2006), previously known from Arauco to Palena provinces (Müller 2009a), this record representing an extension in its distribution range to the south.
Exsiccata: road to Tortel (JL 27889).
Notes: new to Capitán Prat and to Aisén Region.

Notoligotrichum minimum (Cardot) G.L.Sm. (Figure 4A)
On peat or soil in open swampy shrubland or open areas next to roads; alt. 20-120 m a.s.l.; relatively common in the studied area.
Distribution: endemic to southern South America (Schiavone 1993), distributed in Chile from Malleco Province to Cape Horn and in Juan Fernández Islands (Müller 2009a), native also to Brazil (Peralta and Yano 2010).
Exsiccata: Lago Vargas (JL 26837), Caleta Tortel (JL 26895), Lago Leal (JL 27127).
Notes: new to Capitán Prat.

Notoligotrichum trichodon (Hook.f. & Wilson) G.L.Sm.
On bare soil next to roads or in open sites or gaps inside *Nothofagus* forests; alt. 50-90 m a.s.l.; not common in the studied area.
Distribution: an Andean-subantarctic taxon extending north to Colombia, and also in South Georgia, Falkland Is., Kerguelen and the maritime Antarctic (Schiavone 1993; Ochyra et al. 2008), distributed in Chile from Malleco Province to Cape Horn (Müller 2009a).
Exsiccata: Route 7 south of Cochrane km 90 (JL 27922), Río Bravo (JL 26971A).
Notes: new to Capitán Prat.

Oligotrichum austroaligerum G.L.Sm.
On open soil close to the sea shore; alt. 5 m a.s.l.; rare in the studied area.
Distribution: endemic to southern South America (Schiavone 1993), distributed in Chile from Aisén to Magallanes Province (Müller 2009a).
Exsiccata: Puerto Yungay (JL 26531).
Notes: new to Capitán Prat.

Polytrichadelphus magellanicus (Hedw.) Mitt. (Figure 4B)
On soil or rocks in *Nothofagus* forests or ocassionally in open sites; alt. 20-130 m a.s.l.; common in the studied area.
Distribution: endemic to southern South America (Stech et al. 2008), distributed in Chile from Cautín Province to Cape Horn (Müller 2009a).
Exsiccata: Route 7 south of Cochrane km 90 (JL 27919), Lago Vargas (JL 26820), Río Bravo (JL 27005, JL 27145), Lago Leal (JL 27129, JL 27139).
Notes: new to Capitán Prat. This taxon has been considered also distributed in Tasmania, Australia and New Zealand (e.g. Schiavone 1993), but recent molecular studies support the recognition of the Australasian species as a separate taxon (Stech et al. 2008).

Polytrichastrum alpinum (Hedw.) G.L.Sm.
On soil in open swampy shrublands; alt. 50-180 m a.s.l.; rare in the studied area.
Distribution: a bipolar taxon native to Antarctica, several subantarctic islands, New Zealand, SE Australia, Tasmania, southern South America, North America, Europe and Asia (Ochyra et al. 2008), distributed in Chile from Cautín Province to Cape Horn (Müller 2009a).
Exsiccata: Río Carrera (JL 26441), Río Pascua (ET 167).
Notes: new to Capitán Prat and to Aisén Region.

Polytrichastrum longisetum (Sw. ex Brid.) G.L.Sm.
On soil on road banks and in forest or peatland borders, sometimes partly submerged; alt. 30-140 m a.s.l.; common in the studied area.
Distribution: native to Antarctica, southern South America, New Zealand, New Guinea, North America, Europe and Asia (Ochyra et al. 2008), in Chile reported from Valdivia Province to Tierra del Fuego (Müller 2009a).
Exsiccata: Río Bravo (JL 27001), Lago Leal (JL 27128), Lago Quetru (JL 27775), cuesta mirador Baker (JL 26501), road between Cochrane and Tortel, near Vagabundo (JL 26466), Fiordo Mitchell (AJ 310), Lago Vargas (AM 1019b + *Sphagnum magellanicum*), aerodrome at sector Vargas (RR 520).
Notes: new to Capitán Prat.

Polytrichum juniperinum Hedw.
On soil or rock in a variety of habitats from dry steppe to relatively dry deciduous *Nothofagus* forests,

found on road banks, fallen trees, rocks in rivers or swamps; alt. 50-500 m a.s.l.; very common in the studied area.
Distribution: a bipolar taxon with several stations at high altitude in the tropics (Ochyra et al. 2008) distributed in Chile from San Antonio Province in central Chile to Cape Horn (Müller 2009a).
Exsiccata: Río Ñadis (JL 26787c + *Ceratodon purpureus* + *Polytrichum piliferum*), road to Villa O'Higgins (JL 27080), pasarela El Salto (JL 27640), mirador Lago Chacabuco (JL 27687, JL 27693D + *Syntrichia anderssonii* + *Acrocladium auriculatum* + *Cratoneuropsis relaxa* subsp. *minor*), balsa Baker (JL 26680), by road 10 km south of Cochrane (JL 26380), cruce Lago Vargas (JL 26438), río Balboa (RR & ET 70-1, ET 199a + *Polytrichum piliferum*), río Baker (AJ 457a + *Polytrichum strictum*), Cochrane (AM 983A), río Pascua (ET 168a + *Racomitrium geronticum*), Estancia Chacabuco (JL 26930 p.p. + *Ceratodon purpureus*), Caleta Tortel (JL 26898c + *Ceratodon purpureus* + *Campylopus introflexus*).
Notes: new to Capitán Prat.

Polytrichum piliferum Hedw. (Figure 4C)
On soil or rocks in dry habitats next to roads or at the edges of relatively dry *Nothofagus* forests, sometimes inside the forest, on rocks or soil; alt. 60-400 m a.s.l; very common in the studied area.
Distribution: a bipolar taxon with some stations at high altitude in the tropics (Ochyra et al. 2008), distributed in Chile from Talca Province to Cape Horn (Müller 2009a).
Exsiccata: Río Ñadis (JL 26787b + *Ceratodon purpureus* + *Polytrichum juniperinum*), mirador Lago Chacabuco (JL 27688), pasarela Ñadis (JL 27744A), Tamango National Reserve (JL 26610), balsa Baker (JL 26715 p.p. + *Pohlia nutans*), Lago Vargas (AJ 89), puente El Manzano (ET & RR s.n.), Río Balboa (ET 199b + *Polytrichum juniperinum*), Estancia Chacabuco (JL 26941).
Notes: previously reported by Donat (1936a) from west side of Lago O'Higgins (in text, no voucher indicated).

Polytrichum strictum Menzies ex Brid.
A peat-forming taxon found in tundra environments or swampy *Escallonia* shrubland; alt. 50-250 m a.s.l.; not common in the studied area.
Distribution: a bipolar taxon native to southern South America, Antarctica, North America, Europe and Asia (Ochyra et al. 2008), distributed in Chile from Ñuble Province to Cape Horn (Müller 2009a).
Exsiccata: road to Villa O'Higgins (JL 27063), road between Cochrane and Tortel (JL 26445), río Baker (AJ 457b + *Polytrichum juniperinum*).
Notes: new to Capitán Prat and to Aisén Region.

Class Bryopsida Rothm.
Subclass Encalyptidae Ochyra, Żarnowiec & Bednarek-Ochyra
Order Encalyptales Dixon
Family Encalyptaceae Schimp.

Encalypta ciliata Hedw.
On rocks or soil in dry shrubland near steppe areas; alt. 200-400 m a.s.l.; uncommon in the studied area.
Distribution: a subcosmopolitan taxon native to southern South America, Africa, New Guinea, North America, Europe and Asia (Horton 1983), distributed in Chile from Bío-Bío Province to Cape Horn (Müller 2009a).
Exsiccata: Tamango National Reserve (JL 26621), Route 7 south of Cochrane km 26 (JL 26399), puente El Manzano (ET & RR s.n.), Lago Vargas (ET 86a + *Bartramia stricta*).
Notes: new to Capitán Prat.

Encalypta rhaptocarpa Schwägr.
On dry soil amongst grasses and shrubs in dry steppe; alt. 600 m a.s.l.; rare in the studied area.
Distribution: a bipolar species distributed in southern South America, Antarctica, New Zealand, Society Is., North America, Europe and Asia (Ochyra et al. 2008), previously known in Chile only from Magallanes Region (Müller 2009a), so this record represents a northern range extension in Chile.
Exsiccata: Estancia Chacabuco (JL 26957).
Notes: new to Capitán Prat and to Aisén Region.

Subclass Funariidae Ochyra
Order Funariales M.Fleisch.
Family Funariaceae Schwägr.

Funaria hygrometrica Hedw.
On soil in peatlands, tundras, or on the floor of disturbed *Nothofagus* forests; alt. 20-300 m a.s.l.; common in the studied area.
Distribution: a widely distributed cosmopolitan taxon (Ochyra et al. 2008) distributed in Chile from Elqui Province to Cape Horn and in Juan Fernández Is. (Müller 2009a).
Exsiccata: Puerto Yungay (JL 26515b + *Bryum dichotomum*), 10 km south of Cochrane by road (JL 26379), Caleta Tortel (JL 26912 p.p. + *Pohlia wahlenbergii*).
Notes: new to Capitán Prat.

Subclass Dicranidae Doweld
Order Scouleriales Goffinet & W.R.Buck
Family Scouleriaceae S.P.Churchill

Scouleria patagonica (Mitt.) A.Jaeger
On partly submerged rocks in fast flowing rivers; alt. 300 m a.s.l.; rare in the studied area.
Distribution: endemic to southern South America (Churchill 1985), distributed in Chile from Bío-Bío to Capitán Prat Province (Donat 1936a; Müller 2009a).
Exsiccata: Route 7 south of Cochrane at Los Mellizos bridge (JL 27757, JL 27762).
Notes: previously reported by Donat (1936a) from west side of Lago O'Higgins (in text, no voucher indicated).

Order Grimmiales M.Fleisch.
Family Grimmiaceae Arn.

Dryptodon austrofunalis (Müll.Hal.) Ochyra & Żarnowiec
≡*Grimmia austrofunalis* Müll.Hal.
On rocks in open dry shrubland near steppe areas or in dry forest edges; alt. 100-260 m a.s.l.; rare in the studied area.
Distribution: a Gondwanic taxon native also to the Tropical Andes, central Africa and Australasia (Muñoz 1999, Muñoz and Pando 2000), previously known in Chile between Cachapoal and Aisén provinces (Müller 2009a), so this record represents an extension in the southern distribution limit of the species.
Exsiccata: Tamango National Reserve (JL 26646), Balsa Baker (JL 26707).
Notes: new to Capitán Prat.

Dryptodon humilis (Mitt.) Ochyra & Żarnowiec

≡*Grimmia humilis* Mitt.
On rock outcrops in dry steppe; alt. 400-680 m a.s.l.; rare in the studied area, only in steppe habitats.
Distribution: endemic to southern South America (Muñoz 1999; Muñoz and Pando 2000), distributed in Chile between Santiago and Magallanes provinces (Müller 2009a).
Exsiccata: Estancia Chacabuco (JL 26942, JL 26951, JL 26959, JL 26964A).
Notes: new to Capitán Prat.

Dryptodon navicularis (Herzog) Ochyra & Żarnowiec
≡*Grimmia navicularis* Herzog
On rocks in deciduous *Nothofagus pumilio* forest; alt. 400 m a.s.l.; rare in the studied area.
Distribution: an Andean species native to the Tropical Andes of Peru, Bolivia, Ecuador, Colombia, Venezuela, and southern South America (Muñoz 1999), previously known in Chile between Petorca and Aisén provinces (Müller 2009a), so this record represents an extension in the southern distribution of the species.
Exsiccata: Route 7 south of Cochrane km 26 (JL 26396B).
Notes: new to Capitán Prat.

Dryptodon trichophyllus (Grev.) Brid.
≡*Grimmia trichophylla* Grev.
On rocks in deciduous and relatively dry evergreen *Nothofagus* forests or in open areas; alt. 50-400 m a.s.l.; very common in the studied area.
Distribution: a cosmopolitan taxon (Muñoz 1999; Muñoz and Pando 2000) distributed in Chile from Elqui Province to Cape Horn (Müller 2009a).
Exsiccata: front of Laguna Esmeralda (JL 27683A, JL 27683B), mirador Lago Chacabuco (JL 27689A, JL27694, JL 27700, JL 27704), Route 7 south of Cochrane km 90 (JL 27960), Lago Chacabuco (JL 27719), Route 7 south of Cochrane km 26 (JL 26395, JL 26396A), Tamango National Reserve (JL 26611, JL 26627, JL 26658B + *Racomitrium didymum*, JL 26661B + *Racomitrium didymum*), balsa Baker (JL 26681, JL 26693, JL 26703, JL 26714).
Notes: previously reported by Skottsberg (1916) from NE side of Lago O'Higgins as *Grimmia crassiretis* Cardot & Broth. (CS 380).

Racomitrium didymum (Mont.) Lorentz (Figure 3B)
On rocks in wide diversity of habitats, seldom on peat or open soil; alt. 10-360 m a.s.l.; very common in the studied area.
Distribution: taxon widely distributed in the Southern Hemisphere, native to southern South America, the Falkland Is., South Georgia, South Shetland Is., Kerguelen Is., Crozet Is., Prince Edward Is., Heard Is., South Africa, SE Australia and New Zealand (Ochyra et al. 2008), growing in Chile from Talca Province to Cape Horn and in Juan Fernández Is. (Larraín 2012).
Exsiccata: 40 km south of Cochrane by Route 7 (JL 26409A, JL 26410, JL 26415), road between Cochrane and Tortel (JL 26423), río Carrera (JL 26446A, JL 26446D, JL 26447), road S of Cochrane at Tortel/Yungay fork (JL 26474A, JL 26481, JL 26482), mirador Baker (JL 26503A), Puerto Yungay (JL 26517A, JL 26527A, Fiordo Mitchell NE of Puerto Yungay (JL 26554, JL 26558A), Tamango National Reserve (JL 26623, JL 26644B, JL 26658 p.p. + *Dryptodon trichophyllus*, JL 26661A + *Dryptodon trichophyllus*), pasarela Río Ñadis (JL 26779, JL 26783, JL 26786A), pasarela Lago Vargas (JL 26808, JL 26811), between Yungay fork and Caleta Tortel (JL 26842A, JL 26869A), Caleta Tortel (JL 26875D, JL 26929C), Río Bravo (JL 27010), road to Villa O'Higgins (JL 27051, JL 27081), Río Mayer (JL 27090), front of Laguna Esmeralda (JL 27676B, JL 27682A, JL 27684), mirador Lago Chacabuco (JL 27689B, JL 27695, JL 27699A, JL 27701), pasarela Ñadis (JL 27734, JL 27736A, JL 27736B, JL 27738, JL 27742, JL 27750), Lago Quetru (JL 27861B, JL 27878A), Route 7 south of Cochrane km 90

(JL 27928A, JL 27936), El Barrancoso (AM 992).
Notes: new to Capitán Prat.

Racomitrium geronticum Müll.Hal. (Figure 3F)
On rocks, sandy soil or peat in open places, growing in a wide variety of ecological conditions, from dry river banks to very wet peatlands or *Astelia-Donatia* tundras, sometimes dominant in the ground vegetation; alt. 5-260 m a.s.l.; very common in the studied area.
Disribution: as here interpreted, this taxon would have an Andean distribution, extending to the north until Costa Rica, and also distributed in Juan Fernández, Falkland Is., South Georgia, Azores Islands, South Africa and New Zealand (Larraín et al. in preparation), distributed in Chile from Linares Province to Cape Horn (Larraín 2012).
Exsiccata: 40 km south of Cochrane by road (JL 26407, JL 26412, JL 26413), road south of Cochrane at Tortel/Yungay fork (JL 26474B1 + *Racomitrium laevigatum*), mirador Baker (JL 26502B, JL 26504A, JL 26504B), Puerto Yungay (JL 26514, JL 26516, JL 26519, JL 26527D), Tamango National Reserve (JL 26615), road to Tortel just S of Yungay fork (JL 26844), road to Tortel (JL 26869B, JL 26870), Caleta Tortel (JL 26871, JL 26891, JL 26893, JL 26911, JL 26929B), road to Villa O'Higgins (JL 27060, JL 27053, JL 27055B, JL 27057A, JL 27058), road between Lago Leal and Villa O'Higgins fork (JL 27144), Lago Leal (JL 27819), Lago Quetru (JL 27876), Río Bravo (JL 27884), between Vagabundo and Puerto Yungay (AM 1075), Río Pascua (ET 134, ET 168b + *Polytrichum juniperinum*), Río Balboa valley (ET 195).
Notes: previously reported by Donat (1936b) from west side of Lago O'Higgins as *Racomitrium hypnoides* Lindb. (in text, no voucher indicated). Recently Ochyra & Plášek (2016) reported *Racomitrium patagonicum* Bednarek-Ochyra & Ochyra as new for Capitán Prat Province, however my own ongoing taxonomic and molecular studies in the *Racomitrium s.s.* complex would suggest that this taxon may be either a synonym of *R. geronticum* or a subspecies of it.

Racomitrium heterostichoides Cardot
On rocks in open *Donatia-Astelia-Lepidothamnus* tundras; alt. 90 m a.s.l.; rare in the studied area.
Distribution: southern South America, Falkland Is., South Georgia, Kerguelen and Tristan da Cunha (Larraín 2012), in Chile between Osorno Province and Cape Horn (Larraín 2012).
Exsiccata: Caleta Tortel (JL 26872, JL 26875B).
Notes: new to Capitán Prat.

Racomitrium laevigatum A.Jaeger
On rocks or soil in exposed sites adjacent to forests, shrublands or peatlands, ocasionally next to roads; alt. 15-250 m a.s.l.; common in the studied area.
Distribution: a south-temperate taxon distributed in southern South America, the Falkland Islands, South Georgia, Gough Is. and Tristan da Cunha (Bednarek-Ochyra 2006), in Chile between Valdivia Province and Cape Horn, and in Masafuera Is. in the Juan Fernández Archipelago (Larraín 2012).
Exsiccata: Río Carrera bridge (JL 26455), road south of Cochrane at Tortel/Yungay fork (JL 26474B2 + *Racomitrium geronticum*), mirador Baker (JL 26502A), pasarela Lago Vargas (JL 26838), road to Tortel just S of Yungay fork (JL 26843), Río Bravo (JL 27017, JL 27885), road to Villa O'Higgins (JL 27055A, JL 27057B), between Vagabundo and Puerto Yungay (AM 1078).
Notes: previously reported for the province by Donat (1936a) from the west side of Lago O'Higgins as *Racomitrium plicatum* Herzog (AD 16/II).

Racomitrium lamprocarpum (Müll.Hal.) A.Jaeger (Figure 4F)
On rocks in rivers or at lake shores or other water bodies, often submerged; alt. 30-300 m a.s.l.; common in the studied area.

Distribution: a widely-distributed mostly southern hemispheric species, growing in southern South America, along the Andes north to Venezuela, Falkland Is., Kerguelen, Macquire Is., Heard Is., Gough Is., Tristan da Cunha, central and southern Africa and the Iberian Peninsula (Larraín 2012, Ochyra and Van Rooy 2013), distributed in Chile between Talca Province and Cape Horn, and in the Juan Fernández Is. (Müller 2009a).
Exsiccata: road south of Cochrane at Tortel/Yungay fork (JL 26489), Fiordo Mitchell NE of Puerto Yungay (JL 26579), río Carrera (JL 26600), balsa Baker (JL 26734A), Río Bravo (JL 27008), Los Mellizos bridge (JL 27758, JL 27759, JL 27760), Lago Quetru (JL 27857, JL 27861A, JL 27862, JL 27865).
Notes: new to Capitán Prat.

Racomitrium orthotrichaceum (Müll.Hal.) Paris
On rocks in open site next to a *Nothofagus pumilio* forest; alt. 360 m a.s.l.; very rare in the studied area.
Distribution: a south-temperate taxon distributed in southern South America, South Georgia, Gough Is. and Tristan da Cunha (Larraín 2012), in Chile between Valdivia Province and Cape Horn, and in Masafuera Is. in the Juan Fernández Archipelago (Larraín 2012).
Exsiccata: Mirador Lago Chacabuco (JL 27690A).
Notes: new to Capitán Prat.

Racomitrium pachydictyon Cardot
On rocks in open tundras or inside *Nothofagus dombeyi* forest; alt. 70-450 m a.s.l.; rare in the studied area.
Distribution: a south-temperate taxon distributed in southern South America, the Falkland Is., South Georgia, Kerguelen, Prince Edward Is., Crozet Is. and South Shetland Is., and Antarctica (Larraín 2012; Ochyra et al. 2008), in Chile between Aisén Province and Cape Horn (Larraín 2012).
Exsiccata: road to Villa O'Higgins (JL 27070C, JL 27072), pasarela Río Ñadis (JL 26785).
Notes: new to Capitán Prat.

Racomitrium rupestre (Hook.f. & Wilson) Wilson & Hook.f.
On rocks in open tundras or in dry shrublands; alt. 260-450 m a.s.l.; rare in the studied area.
Distribution: A south-temperate taxon distributed in southern South America (Larraín 2012), South Georgia (Bell 1974), Tasmania (Dalton et al. 1991), New Zealand (Dixon 1926) and Kerguelen (Cardot 1908, 1916), reported in Chile between Ñuble Province and Cape Horn (Larraín 2012).
Exsiccata: Tamango National Reserve (JL 26657B), road to Villa O'Higgins (JL 27070A).
Notes: new to Capitán Prat.

Racomitrium subcrispipilum (Müll.Hal.) A.Jaeger
=*Racomitrium striatipilum* Cardot
On rocks inside evergreen *Nothofagus-Pilgerodendron* forests or in deciduous *Nothofagus pumilio* forest, or in open sites next to forests or in open tundras; alt. 5-400 m a.s.l.; very common in the studied area.
Distribution: a south-temperate taxon native to southern South America, the Falkland Is., South Georgia (Larraín 2012), Crozet Is. (Bednarek-Ochyra and Ochyra 2009), Tristan da Cunha (Bednarek-Ochyra 2011), and Kerguelen (Bednarek-Ochyra and Ochyra 2010), reported in Chile between Ñuble Province and Cape Horn, as well as in Masafuera Is. in Juan Fernández Archipelago (Larraín 2012).
Exsiccata: 26 km S of Cochrane, by road (JL 26398A), 40 km S of Cochrane, by road (JL 26411, JL 26414), road between Cochrane and Tortel (JL 26422), Río Carrera bridge (JL 26446C), Fiordo Mitchell NE of Puerto Yungay (JL 26545), Tamango National Reserve (JL 26644A), pasarela Río Ñadis (JL 26786B), pasarela Lago Vargas (JL 26819), Caleta Tortel (JL 26875A, JL 26890, JL 26894,

JL 26909), road to Villa O'Higgins (JL 27059), S of Cochrane S of Laguna Esmeralda (JL 27676C), mirador Lago Chacabuco (JL 27690B), Lago Chacabuco (JL 27715), Lago Quetru (JL 27866), Río Bravo (JL 27883), Route 7 south of Cochrane km 90 (JL 27969), road S of Los Ñadis (AM 1010).
Notes: new to Capitán Prat.

Schistidium andinum (Mitt.) Herzog
On rocks in dry steppe; alt. 400-680 m a.s.l.; rare in the studied area.
Distribution: an Andean-south-temperate taxon growing in southern South America, along the Andes north to Ecuador, and also in South Georgia and the maritime Antarctic (Ochyra et al. 2008), distributed in Chile between Valdivia Province and Cape Horn (Müller 2009a).
Exsiccata: Estancia Chacabuco (JL26933, JL 26963).
Notes: first reported for the province by Cardot and Brotherus (1923), as *Grimmia occulta* Müll.Hal. from NW side of Lago San Martín [=Lago O'Higgins] (CS 364).

Family Seligeriaceae Schimp.

Blindia contecta (Hook.f. & Wilson) Müll.Hal.
On irrigated rocks in beach next to a *Drimys-Weinmannia-Podocarpus* forest; alt. 5 m a.s.l.; rare in the studied area.
Distribution: a south-temperate taxon native to southern South America, Campbell and Auckland Islands, Kerguelen Is. and Heard Is., extending north to Easter Island (Andreas 2013), distributed in Chile between Aisén and Magallanes provinces (Müller 2009a).
Exsiccata: Fiordo Mitchell NE of Puerto Yungay (JL 26552).
Notes: new to Capitán Prat.

Blindia magellanica Schimp.
On irrigated rocks along rivers or creeks, ocassionally on wet soil on river banks; alt. 30-400 m a.s.l.; common in the studied area.
Distribution: a south-temperate taxon widely distributed in the Southern Hemisphere, extending to the tropics in Ecuador, Colombia, Brazil, Easter Island and New Guinea (Bartlett and Vitt 1986, Andreas 2013), distributed in Chile between Talca Province and Cape Horn (Müller 2009a).
Exsiccata: 20 km S of Cochrane by road (JL 26388), balsa Baker (JL 26692, JL 26710, JL 26741), road to Tortel (JL 27856), Lago Quetru (JL 27858, JL 27859).
Notes: first reported for Capitán Prat Province by Skottsberg (1916) from the NW side of Lago O'Higgins (CS 250).

Order Dicranales H. Philib. ex M. Fleisch.
Family Fissidentaceae Schimp.

Fissidens curvatus Hornsch.
On rocks inside a *Nothofagus dombeyi-Embothrium coccineum-Amomyrtus luma* forest, with *Fuchsia* and *Chusquea* in the understory; alt. 50 m a.s.l.; rare in the studied area.
Distribution: a widespread species found in southern South America, New Zealand, Australia, New Caledonia, throughout the Americas, Southern and Eastern Africa, Asia, and Europe (Beever et al. 2002; Müller and Pursell 2003), previously known in Chile between El Loa and Aisén provinces and in Juan Fernández (Müller 2009a), so this record represents an extension in the southern distribution limit of the species.
Exsiccata: Route 7 south of Cochrane km 90 (JL 27962, JL 27972A + *Eurhynchiella acanthophylla*).
Notes: new to Capitán Prat.

Fissidens rigidulus Hook.f. & Wilson
On wet soil in river bank inside a *Nothofagus dombeyi* forest; alt. 100 m a.s.l.; rare in the studied area.
Distribution: an Andean-south-temperate species distributed in southern South America, New Zealand, Australia, Malesia, the SW Pacific, subantarctic islands, and extending north along the Andes into Central America and Mexico (Müller and Pursell 2003), reported in Chile between Petorca Province and Cape Horn (Müller 2009a).
Exsiccata: balsa Baker (JL 26709).
Notes: new to Capitán Prat.

Family Eustichiaceae Broth.

Eustichia longirostris (Brid.) Brid
On irrigated road bank; alt. 380 m a.s.l.; rare in the studied area.
Distribution: an Andean-African taxon, growing along the Andes into Central America, the Caribbean and Brazil, also and in the Subantarctic Islands, South Africa, Middle Atlantic Ocean and the Western Indian Ocean (Allen 2002), distributed in Chile between Petorca and Magallanes provinces and in Juan Fernández Is. (Müller 2009a).
Exsiccata: Route 7 south of Cochrane km 20 (JL 26393).
Notes: new to Capitán Prat.

Family Ditrichaceae Limpr.

Ceratodon purpureus (Hedw.) Brid. subsp. ***purpureus***
Growing in a wide variety of habitats, both disturbed or pristine, on soil in evergreen and deciduous *Nothofagus* forest floor, or in the forest edge or in open areas, sometimes on rocks or fallen trees, seldom on dry soil amongst grasses and shrubs in dry steppe; alt. 20-500 m a.s.l.; very common in the studied area.
Distribution: a cosmopolitan species (Ochyra et al. 2008) distributed in Chile between Limarí Province and Cape Horn (Müller 2009a).
Exsiccata: Route 7 south of Cochrane km 20 (JL 26386), Tamango National Reserve (JL 26635), balsa Baker (JL 26701 p.p. + *Pohlia nutans*, JL 26729), pasarela Río Ñadis (JL 27733, JL 26787 p.p. + *Polytrichum juniperinum* + *P. piliferum*), pasarela Lago Vargas (JL 26810, JL 26821), Caleta Tortel (JL 26923b + *Brachytheciastrum paradoxum*, JL 26898b + *Polytrichum juniperinum* + *Campylopus introflexus*), Estancia Chacabuco (JL 26930b + *Polytrichum juniperinum*), Río Bravo (JL 27031), past Villa O'Higgins by road front of río Mayer (JL 27097), Lago Leal (JL 27133B + *Chorisodontium aciphyllum* + *Campylopus pyriformis* + *Zygodon reinwardtii*, JL 27805b + *Bryum* sp.), pasarela El Salto (JL 27639), by road front of Laguna Esmeralda (JL 27672), mirador Lago Chacabuco (JL 27691A, JL 27696), Route 7 south of Cochrane km 90 (JL 27924, JL 27934 p.p. + *Pohlia nutans*), Río El Paso (AJ 1A).
Notes: new to Capitán Prat.

Ceratodon purpureus subsp. ***convolutus*** (Reichardt) Burley
On rock crevices in dry steppe, on exposed road banks or on fallen trees at the edge of *Podocarpus-Amomyrtus-Nothofagus* forests; alt. 15-670 m a.s.l.; relatively rare in the studied area.
Distribution: a sub-cosmopolitan species distributed in South, Central and North America, south and central Africa, Azores Is., the Mediterranean and south Europe, south India, the Himalayas, SW China, and SE Asia (Burley and Pritchard 1990), reported in Chile between Ñuble and Tierra del Fuego provinces (Müller 2009a).

Exsiccata: In Tortel/Yungay fork, S of Cochrane (JL 26469), road between Route 7 and Tortel (JL 26850), Estancia Chacabuco (JL 26954).
Notes: new to Capitán Prat.

Chrysoblastella chilensis (Mont.) Reimers
On soil on road banks or inside *Nothofagus* forests or in exposed river banks or *Escallonia* shrubland, ocassionally on rocks; alt. 20-240 m a.s.l.; common in the studied area.
Distribution: an Andean-south-temperate species distributed along the Andes from Magallanes to Colombia, reported for Brazil and Dominican Republic, also native to Tristan da Cunha, Marion Is., South Georgia, Macquarie Is., New Zealand and SE Australia (Ochyra 1999), reported in Chile between Talca and Magallanes provinces (Müller 2009a).
Exsiccata: road between Cochrane and Tortel (JL 26416, JL 26419A, JL 26421), Río Carrera bridge (JL 26449A), Tortel/Yungay fork (JL 26471, JL 26487), pasarela Lago Vargas (JL 26818, JL 26832), road to villa O'Higgins (JL 27061), Lago Chacabuco (JL 27707), pasarela Ñadis (JL 27740, JL 27741A + *Philonotis scabrifolia*), Lago Quetru (JL 27879), Route 7 south of Cochrane km 90 (JL 27923 + *Ditrichum* sp.).
Notes: new to Capitán Prat and to Aisén Region.

Distichium capillaceum (Hedw.) Bruch & Schimp.
On soil in road banks or open ground in the edge of *Nothofagus* forests; alt. 50-210 m a.s.l.; not common in the studied area.
Distribution: a sub-cosmopolitan species growing throughout the Americas, northern Europe, northern and central Asia, Japan, New Guinea, Australia and New Zealand (Crum and Anderson 1981), distributed in Chile between Curicó Province and Cape Horn (Müller 2009a).
Exsiccata: Lago Quetru (JL 27774), road to Puerto Yungay before Vagabundo (JL 26965A1 + *Bartramia patens*).
Notes: first reported for the province by Skottsberg (1916) from the NW side of Lago O'Higgins (CS 279), also known from Tamango National Reserve (Cuvertino et al. 2012).

Ditrichum cylindricarpum (Müll.Hal.) F.Muell.
On rocks in shaded forest habitats often near creeks or small rivers, seldom on soil, road banks, or fallen logs; alt. 5-400 m a.s.l.; common in the studied area.
Distribution: a south-temperate species growing in southern South America, SW Australia, Tasmania and New Zealand (Seppelt 1982), distributed in Chile between Ñuble Province and Cape Horn (Müller 2009a).
Exsiccata: road S of Cochrane at Tortel/Yungay fork (JL 26477, JL 26499, JL 27916), Fiordo Mitchell NE of Puerto Yungay (JL 26548), road to Villa O'Higgins (JL 27078), passed Villa O'Higgins by road front of río Mayer (JL 27087), Lago Leal (JL 27811).
Notes: new to Capitán Prat.

Ditrichum difficile (Duby) M.Fleisch.
On soil on road banks or forest trails, seldom on roots; alt. 20-50 m a.s.l.; not common in the studied area.
Distribution: a sub-cosmopolitan species growing in southern South America, South Africa, Madagascar, Indonesia, India, Asia, Taiwan, Australia, Tasmania and New Zealand (Seppelt 1982), distributed in Chile between Linares Province and Tierra del Fuego, as well as in Juan Fernández Is. and Easter Is. (Müller 2009a).
Exsiccata: pasarela Lago Vargas (JL 26815), Lago Leal (JL 27814), Route 7 south of Cochrane km 90 (JL 27931A).

Notes: new to Capitán Prat.

Ditrichum ditrichoideum (Cardot) Ochyra
≡*Austrophilibertiella ditrichoidea* (Cardot) Ochyra
On soil on forest edge or open disturbed places; alt. 5-60 m a.s.l.; not common in the studied area.
Distribution: a south-temperate taxon native to southern South America, the maritime Antarctic, Prince Edward Is., Marion Is., Crozet Is. and Kerguelen (Ochyra et al. 2008), distributed in Chile between Valdivia and Magallanes provinces (Müller 2009a).
Exsiccata: Puerto Yungay (JL 26532), pasarela Ñadis (JL 27739).
Notes: new to Capitán Prat.

Ditrichum heteromallum (Hedw.) E.Britton
On soil on forest edges or open disturbed places, seldom on rocks in open sites; alt. 5-250 m a.s.l.; not common in the studied area.
Distribution: a bipolar species growing in southern South America, the Falkland Is., South Georgia, the maritime Antarctic, Europe, Asia, and western North America (Ochyra et al. 2008), distributed in Chile between Aisen Province and Cape Horn (Müller 2009a).
Exsiccata: Puerto Yungay (JL 26535), road to Villa O'Higgins (JL 27056A), Lago Leal (JL 27130), Lago Quetru (JL 27777B).
Notes: new to Capitán Prat.

Family Dicranaceae Schimp.

Camptodontium cryptodon (Mont.) Reimers
On fallen logs and roots in relatively dry deciduous *Nothofagus pumilio* forests; alt. 70-400 m a.s.l.; common in the studied area.
Distribution: a southern South American endemic taxon (Reimers 1936), distributed in Chile from Cordillera Province to Magallanes Province (Müller 2009a).
Exsiccata: along Route 7 S of Cochrane km 10 (JL 26381B + *Orthotrichum pariatum*), Tamango National Reserve (JL 26645, JL 26652), balsa Baker (JL 26695, JL 26742), pasarela El Salto (JL 27645, JL 27646, JL 27648B + *Orthotrichum incanum*, JL 27650).
Notes: new to Capitán Prat.

Campylopodium euchlorum (Mont.) Matteri
On soil or fallen trees in shady evergreen *Nothofagus* forests; alt. 20-90 m a.s.l.; rare in the studied area.
Distribution: an endemic species of the southern Andes (Matteri and Calabrese 1999), previously known in Chile between Valdivia and Aisén provinces and in Juan Fernández Is. (Müller 2009a), so this record represents an extension in the southern distribution limit of the species.
Exsiccata: Route 7 south of Cochrane km 90 (JL 27931B, JL 27933), Río Bravo (JL 26985, JL 27146 p.p. + *Campylopodium medium*).
Notes: new to Capitán Prat.

Campylopodium medium (Duby) Giese & J.-P.Frahm
On soil or seldom on rocks in road banks in shady evergreen *Nothofagus* forest habitats; alt. 20-70 m a.s.l.; rare in the studied area.
Distribution: a taxon with an uncommon distribution in SE Asia (Indonesia, Malaysia, Philippines, New Guinea), New Zealand, the South Pacific (Samoa, Society Is., Fiji, New Caledonia), Japan, Taiwan, Hawaii, Puerto Rico, and Chile (Giese and Frahm 1985); previously known in Chile between

Arauco and Aisén provinces (Müller 2009a), so these records represent an extension in the southern distribution limit of the species.
Exsiccata: Río Bravo (JL 27146 p.p. + *Campylopodium euchlorum*), Lago Quetru (JL 27116, JL 27777A), Lago Leal (JL 27812).
Notes: new to Capitán Prat.

Chorisodontium aciphyllum (Hook.f. & Wilson) Broth.
On soil, rocks or fallen logs in shady evergreen *Nothofagus dombeyi* forests; alt. 20-340 m a.s.l.; common in the studied area.
Distribution: a south-temperate species native to southern South America, the Falkland Islands, South Georgia, Antarctica, Tristan da Cunha, Amsterdam Is., Kerguelen Is. and New Zealand (Hyvönen 1991; Ochyra et al. 2008), distributed in Chile from Cauquenes Province to Cape Horn, as well as in Juan Fernandez (Müller 2009a).
Exsiccata: Río Carrera (JL 26602), pasarela Río Ñadis (JL 26781), pasarela Lago Vargas (JL 26816), Río Bravo (JL 26976, JL 27013), Lago Quetru (JL 27102), Lago Leal (JL 27133C + *Ceratodon purpureus* + *Campylopus pyriformis* + *Zygodon reinwardtii*), Lago Chacabuco (JL 27723, JL 27727).
Notes: first reported for the province by Skottsberg (1916) as *Dicranum lanigerum* Müll.Hal. from the NW side of Lago O'Higgins (CS 167).

Chorisodontium dicranellatum (Dusén) Roiv.
Either epiphytic on dead truncks or branches in evergreen *Nothofagus dombeyi-Podocarpus-Drimys* forests, or on rocks or humid soil in exposed habitats; alt. 30-130 m a.s.l.; not common in the studied area.
Distribution: an endemic taxon of the southern Andes (Hyvönen 1991), distributed in Chile between Cautín Province and Cape Horn (Müller 2009a).
Exsiccata: Cuesta Barrancoso (JL 26408), Río Bravo (JL 27030D + *Ulota pusilla* + *Matteria papillosula* + *Weymouthia mollis*), Lago Quetru (JL 27784, JL 27790, JL 27793C + *Matteria papillosula* + *Ulota rufula* + *U. pygmaeothecia*, JL 27824).
Notes: new to Capitán Prat.

Chorisodontium spegazzinii (Müll.Hal.) Roiv.
On peaty soil in *Donatia-Astelia-Lepidothamnus* tundras; alt. 100 m a.s.l.; extremely rare in the studied area.
Distribution: an endemic taxon of southern Chile and Argentinian Tierra del Fuego (Hyvönen 1991, Matteri 2003), previously known in Chile between Última Esperanza Province and Cape Horn (Müller 2009a), so this record represents an extension in the northern distribution limit of the species.
Exsiccata: Caleta Tortel (JL 26914).
Notes: new to Capitán Prat.

Dicranella campylophylla (Taylor) A.Jaeger
=*Dicranella cardotii* (R.Br.bis) Dixon
On very humid soil along rivers and streams or irrigated road banks, generally at forest edges, seldom growing at the bases of trees or shrubs or fallen trees; alt. 70-430 m a.s.l.; common in the studied area.
Distribution: an Andean-south-temperate species occurring in the northern Andes, southern South America, several subantarctic islands, the maritime Antarctic, South Africa, and temperate Australasia (Ochyra and Newton 1985; Ochyra et al. 2008), growing in Chile from Aconcagua Province to Tierra del Fuego and Easter Island (Müller 2009a).
Exsiccata: by road 20 km S of Cochrane (JL 26390), balsa Baker (JL 26705, JL 26711), road to Villa O'Higgins by Lago O'Higgins (JL 27074), by road past Villa O'Higgins at Río Mayer (JL 27091), Lago

Quetru (JL 27122B), road front of Laguna Esmeralda (JL 27685), Puerto Río Bravo (AM 1037A).
Notes: new to Capitán Prat.

Dicranella hookeri (Müll.Hal.) Cardot
On wet soil in river bank along *Nothofagus antarctica* forest patches; alt. 430 m a.s.l.; rare in the studied area.
Distribution: an Andean-south-temperate species occurring in the northern Andes, southern South America, the maritime Antarctic, some subantarctic islands, South Africa, and temperate Australasia (Ochyra et al. 2008), present in Chile from Huasco Province to Cape Horn (Larraín et al. 2010), also in Easter Island (Ireland and Bellolio 2002).
Exsiccata: Estancia Chacabuco (JL 26949A + *Campylium stellatum*, JL 26947).
Notes: new to Capitán Prat.

Dicranella pseudorufescens Cardot & Broth.
On humid soil on trails or trail banks inside *Nothofagus dombeyi-Drimys winteri-Podocarpus nubigena* forests; alt. 50-90 m a.s.l.; rare in the studied area.
Distribution: a Chilean endemic taxon previously known from just two collections from one single locality at Isla Riesco, Magallanes Province (Larraín et al. 2010), so these records represent an extension in the northen distribution limit of the species.
Exsiccata: Río Bravo (JL 26971B), Lago Quetru (JL 27776).
Notes: new to Capitán Prat and to Aisén Region. This is the second reported locality for the species after its original description by Cardot and Brotherus (1923).

Dicranoloma billardieri (Brid.) Paris
On peaty soil at the margins of peatlands or swamps, on soil on the forest floor or seldom epiphytic on *Weinmannia-Nothofagus-Podocarpus* forests; alt. 5-70 m a.s.l.; common in the studied area.
Distribution: a south-temperate species native to the Falkland Islands (Matteri 2003), southern South America, Australia, New Zealand, eastern Africa, Madagascar, and sub-Antarctic islands (Klazenga 2003), distributed in Chile from Valparaíso to Tierra del Fuego, as well as in Juan Fernandez (Müller 2009a).
Exsiccata: road between Cochrane and Tortel at Lago Vargas fork (JL 26435), near Vagabundo (JL 26468), Fiordo Mitchell NE of Puerto Yungay (JL 26550 p.p. + *Dicranoloma imponens*, JL 26553), Lago Quetru (JL 27099).
Note: first reported for the province by Cardot and Brotherus (1923) from Caleta Hale (CS 188).

Dicranoloma chilense (De Not.) Ochyra & Matteri
On soil in the shady floor of evergreen *Nothofagus dombeyi* forests; alt. 30-210 m a.s.l.; rare in the studied area.
Distribution: an endemic southern South American species distributed in the Argentinan Tierra del Fuego and the Falkland Islands (Matteri 2003), and in Chile from Valparaíso Province to Cape Horn, as well as in Juan Fernandez Is. (Müller 2009a).
Exsiccata: Lago Quetru (JL 27853).
Notes: new to Capitán Prat.

Dicranoloma imponens (Mont.) Renauld
On soil in tundras or peatlands, ocassionally in the floor of very wet forests; alt. 20-450 m a.s.l.; common in the studied area.
Distribution: an endemic southern South American species distributed in in the Argentinan Tierra del Fuego (Matteri 2003) and in Chile from Valdivia Province to Cape Horn (Müller 2009a).

Exsiccata: road between Cochrane and Tortel near Vagabundo (JL 26458), Puerto Yungay (JL 26512), Fiordo Mitchell NE of Puerto Yungay (JL 26550b + *Dicranoloma billardieri*), Caleta Tortel (JL 26880), road to Villa O'Higgins (JL 27067).
Notes: new to Capitán Prat and to Aisén Region.

Dicranoloma menziesii (Hook.f. & Wilson) Paris
Epiphytic on base of *Embothrium coccineum* in *Nothofagus dombeyi-Embothrium-Amomyrtus* forest; alt. 50 m a.s.l.; rare in the studied area.
Distribution: a south-temperate species growing in southern South America, Australia, Tasmania, New Zealand and New Caledonia (Klazenga 2003), distributed in Chile between Valdivia and Magallanes provinces, as well as in Juan Fernandez (Müller 2009a).
Exsiccata: Route 7 south of Cochrane km 90 (JL 27951).
Notes: new to Capitán Prat.

Dicranoloma perremotifolium (Dusén) Broth.
On fallen log in a river, very wet habitat; alt. 50 m a.s.l.; rare in the studied area.
Distribution: a Chilean endemic taxon distributed between Bío-Bío and Aisén provinces (Müller 2009a), so this record represents an extension in the southern distribution limit of the species.
Exsiccata: Lago Quetru (JL 27782).
Notes: new to Capitán Prat. This taxon is very close to *D. robustum*, but differs from it by the thinner stems, the narrower and much longer and patent leaves, which are widely spaced along stems, forming very loose and big mats often pendent from tree trunks. It might be a synonym of *D. robustum*, but it is here retained as a different taxon until the genus is revised in the area as a whole.

Dicranoloma robustum (Hook.f. & Wilson) Paris
On the forest floor in *Drimys-Weinmannia-Podocarpus* forests, or in *Nothofagus dombeyi* forest, sometimes epiphytic or on fallen trees inside the forest; alt. 5-340 m a.s.l.; common in the studied area.
Distribution: a south-temperate species distributed in southern South America, Australia, Tasmania, Macquarie Is., New Zealand, Campbell, Stwart and Auckland Is., and sub-Antarctic islands (Klazenga 2003), reported in Chile from Cautín Province to Cape Horn (Müller 2009a).
Exsiccata: road between Cochrane and Tortel near Vagabundo (JL 26463), Fiordo Mitchell NE of Puerto Yungay (JL 26542, JL 26569), Río Carrera (JL 26592, JL 26598, JL 26606), road between Cochrane and Tortel (JL 26425, JL 26427), Río Bravo (JL 26996, JL 27016), Lago Leal (JL 27138), S of Cochrane front of Laguna Esmeralda (JL 27662), Lago Chacabuco (JL 27711), Pasarela Ñadis (JL 27749), Lago Quetru (JL 27778, JL 27794, JL 27823A, JL 27828, JL 27840), road between Route 7 and Tortel (JL 27908), Route 7 south of Cochrane km 90 (JL 27945).
Notes: first reported for the province by Cardot & Brotherus (1923) as *Dicranoloma australe* (Besch.) Paris from Caleta Hale (CS 175).

Eucamptodon perichaetialis (Mont.) Mont.
Epiphytic on *Drimys*, *Nothofagus antarctica* or fallen trees, twigs or branches, inside the forest or on isolated trees in open sites, restricted to very humid lowland forests; alt. 30-130 m a.s.l.; uncommon in the studied area.
Distribution: a southern South American endemic species (Allen 1987) distributed in Chile from Talca to Tierra del Fuego provinces, as well as in Juan Fernandez (Müller 2009a).
Exsiccata: Río Bravo (JL 27024), Lago Quetru (JL 27114, JL 27787B + *Daltonia gracilis* + *Hypnum cupressiforme* var. *filiforme* + *Ulota rufula* + *Weymouthia cochlearifolia*, JL 27788E + *Hypnum cupressiforme* var. *filiforme* + *Weymouthia mollis* + *Zygodon pentastichum* + *Z. reinwardtii*, JL 27867).

Notes: new to Capitán Prat.

Kiaeria pumila (Mitt.) Ochyra
Epiphytic on *Nothofagus antarctica* or on rocks inside *Nothofagus dombeyi* forests; alt. 20-50 m a.s.l.; rare in the studied area.
Distribution: a pan-south-temperate species growing in southern South America, Falkland Is., South Georgia, Amsterdam Is., Tristan da Cunha, Prince Edward Is. and New Zealand (Ochyra et al. 2008) distributed in Chile between Aisén Province and Cape Horn (Müller 2009a).
Exsiccata: Route 7 south of Cochrane km 90 (JL 27966), pasarela Lago Vargas (JL 26823).
Notes: new to Capitán Prat.

Pilopogon schilleri Herzog & Thér.
On soil either inside *Nothofagus* or other evergreen forests or in exposed sites, often in road banks; alt. 20-210 m a.s.l.; relatively common in the studied area.
Distribution: a southern South American endemic species (Frahm 1983; Suárez and Schiavone 2009) distributed in Chile between Chiloé and Magallanes provinces (Müller 2009a).
Exsiccata: Road S of Cochrane at Tortel/Tungay fork (JL 26476B), Río Bravo (JL 27003), pasarela Lago Vargas (JL 26822).
Notes: new to Capitán Prat and to Aisén Region.

Platyneuron praealtum (Mitt.) Ochyra & Bednarek-Ochyra (Figure 3H)
On soil on the forest floor or at forest edges, in both evergreen and deciduous *Nothofagus* forests, seldom on fallen trees; alt. 20-400 m a.s.l.; common in the studied area.
Distribution: a south-temperate species widely distributed in southern South America, extending to South Georgia, South Africa and Kerguelen Is. (Ochyra and Bednarek-Ochyra 1997), reported in Chile between Ñuble Province and Cape Horn (Müller 2009a).
Exsiccata: road between Route 7 and Tortel (JL 26424), balsa Baker (JL 26736), pasarela Río Ñadis (JL 26776, JL 27751), pasarela Lago Vargas (JL 26800), road S of Cochrane front of Laguna Esmeralda (JL 27667), Lago Chacabuco (JL 27710), Puente Santo Domingo, Río El Paso (AM 1028), Río Bravo (AM 1046B).
Notes: new to Capitán Prat.

Family Leucobryaceae Schimp.

Campylopus acuminatus Mitt.
On humid soil or submerged in tundras and peatlands; alt. 20-90 m a.s.l.; rare in the studied area.
Distribution: a south-temperate species distributed in southern South America, New Zealand, southern Australia and Tasmania (Frahm 1988, 1994), known in Chile from Malleco Province to Cape Horn (Müller 2009a).
Exsiccata: Puerto Yungay (JL 26511), Caleta Tortel (JL 26878).
Notes: new to Capitán Prat.

Campylopus clavatus (R.Br.) Wilson
In open sites on peaty soil, fallen trees or road banks; alt. 20-140 m a.s.l.; uncommon in the studied area.
Distribution: a south-temperate species distributed in southern South America, New Zealand including Auckland and Campbell Is., southern Australia, Tasmania, Prince Edward Is. (Frahm 1984) and South Africa (O'Shea 2006), known in Chile from Valparaíso Province to Cape Horn, and in Juan Fernández and Easter Is. (Müller 2009a).

Exsiccata: Lago Quetru (JL 27874), road to Tortel south of Yungay fork (JL 26845), road between Tortel/Yungay fork and Puerto Yungay (JL 26503B).
Notes: new to Capitán Prat.

Campylopus incrassatus Müll.Hal.
On open soil in disturbed place by the sea shore; alt. 5 m a.s.l.; very rare in the studied area.
Distribution: a south-temperate species distributed in southern South America, southern Australia, New Zealand, St. Paul, Gough Is., Ascension Is., and Madeira (Frahm 1988), reported in Chile from Malleco Province to Cape Horn, and in Juan Fernández and Easter Is. (Müller 2009a).
Exsiccata: Puerto Yungay (JL 26530).
Notes: new to Capitán Prat.

Campylopus introflexus (Hedw.) Brid.
On open soil in disturbed places, on road banks, on tundras or on fallen trees in open places; alt. 5-190 m a.s.l.; very common in the studied area.
Distribution: a south-temperate species known from southern South America, southern Australia, Tasmania, New Zealand, South Africa, the South Sandwich Is. and virtually all sub-antarctic islands, also introduced in western Europe from the British Is. to Poland (Ochyra et al. 2008), distributed in Chile from Limarí Province to Cape Horn, and in Juan Fernández and Easter Is. (Müller 2009a).
Exsiccata: Río Carrera (JL 26443), road S of Cochrane at Tortel/Yungay fork (JL 26475), Puerto Yungay (JL 26534, JL 26537), pasarela Río Ñadis (JL 26794), road to Tortel (JL 26850B), Caleta Tortel (JL 26899, JL 26898d + *Ceratodon purpureus* + *Polytrichum juniperinum*), Lago Quetru (JL 27103, JL 27822B), Lago Leal (JL 27807), Route 7 south of Cochrane km 90 (JL 27917).
Notes: new to Capitán Prat.

Campylopus purpureocaulis Dusén
On soil or fallen trees in open sites (Frahm 1988); very rare in the studied area.
Distribution: a south-temperate species known from southern South America, New Zealand and Marion Is. (Bartlett and Frahm 1983), distributed in Chile between Concepción and Magallanes provinces (Müller 2009a).
Notes: reported by Skottsberg (1916) from Caleta Hale (CS 216), not found among my collections.

Campylopus pyriformis (Schultz) Brid.
On open soil in shrublands, on fallen trees and seldom on animal dung; alt. 50-90 m a.s.l.; rare in the studied area.
Distribution: a bipolar temperate species widely distributed in the Northern Hemisphere and in southern South America, sub-antarctic islands and in continental Antarctica (Ochyra et al. 2008), Australia (Frahm 1994), New Zealand (Beever et al. 1992), New Caledonia (Frahm 1990), Brazil (Frahm 1991), previously known in Chile between Choapa and Aisén provinces, and in Juan Fernández Is. (Müller 2009a), so this record represents an extension in the southern distribution limit of the species.
Exsiccata: Río Carrera (JL 26454), Caleta Tortel (JL 26886B), Lago Leal (JL 27133A + *Ceratodon purpureus* + *Chorisodontium aciphyllum* + *Zygodon reinwardtii*).
Notes: new to Capitán Prat.

Campylopus vesticaulis Mitt.
On peaty soil in peatlands, seldom on rocks in exposed sites; alt. 50-90 m a.s.l.; relatively common in the studied area.
Distribution: a south-temperate species known from southern South America, the Falkland Is., Tristan

da Cunha, Marion Is., New Zealand and the South Sandwich Is. in the maritime Antarctic (Ochyra et al. 2008), distributed in Chile from Concepción Province to Tierra del Fuego, and in Juan Fernández and Easter Is. (Müller 2009a).
Exsiccata: Río Carrera (JL 26452), road S of Cochrane at Tortel/Yungay fork (JL 26483), Caleta Tortel (JL 26887, JL 26892).
Notes: new to Capitán Prat.

Family Oncophoraceae Stech

Amphidium tortuosum (Hornsch.) Cufod.
On shaded rocks near creek; alt. 50 m a.s.l.; rare in the studied area.
Distribution: a widely distributed taxon growing along the Andes from Magallanes into the Neotropics, and in western North America to British Columbia, Hawaii, south, central and western Africa, Canary Is., Madeira, New Guinea, Australia and Tasmania (Frahm et al. 2000), reported in Chile between Valparaíso and Magallanes provinces, as well as Juan Fernández (Müller 2009a).
Exsiccata: balsa Baker (JL 26685A + *Zygodon magellanicus*).
Notes: new to Capitán Prat.

Hymenoloma crispulum (Hedw.) Ochyra
≡*Dicranoweisia crispula* (Hedw.) Milde
On rocks; very rare in the studied area, but eventually more common around glaciers.
Distribution: a bipolar species with a continuous distribution in temperate areas of the Northern Hemisphere and scattered in altimontane regions in warmer areas of Europe, Asia and the Neotropics, growing in southern South America and extending into the maritime Antarctic and the Antarctic Peninsula (Ochyra et al. 2008), distributed in Chile from Llanquihue Province to Cape Horn (Müller 2009a).
Notes: reported by Donat (1936a) as *Dicranoweisia austro-crispula* (Müll.Hal.) Paris from west side of Lago O'Higgins (AD s.n.).

Hymenoloma turpe (Cardot) Cardot & Broth.
≡*Verrucidens turpis* (Cardot) Cardot
On dry soil in sub-steppe habitat; 220 m a.s.l.; rare in the studied area.
Distribution: a southern South American endemic taxon distributed in Chile between Aisén and Magallanes provinces (Müller 2009a).
Exsiccata: Tamango National Reserve (JL 26631).
Notes: first reported for the province by Donat (1936a) from the W side of Lago O'Higgins (AD s.n.).

Order Pottiales M.Fleisch.
Pottiaceae Schimp.

Calyptopogon mnioides (Schwägr.) Broth.
Epiphytic on *Nothofagus dombeyi*, *Fuchsia magellanica* and seldom on other tree species, or in rocks in open sites; alt. 5-50 m a.s.l.; common in the studied area.
Distribution: a south-temperate species native to southern South America, Australia, Tasmania and New Zealand (Zander 1993), distributed in Chile from Valaparaíso Province to Cape Horn (Müller 2009a).
Exsiccata: road S of Cochrane at Tortel/Yungay fork (JL 26470b + *Lepyrodon patagonicus*, JL 26480), Caleta Tortel (JL 26926), Route 7 south of Cochrane km 90 (JL 27938b + *Cryphaea consimilis*).
Notes: new to Capitán Prat.

Didymodon andreaeoides Cardot & Broth.
On rocks or wet soil in exposed places; alt. 30-350 m a.s.l.; rare in the studied area.
Distribution: a southern South American endemic taxon distributed in Chile between Cautín and Magallanes provinces (Müller 2009a).
Exsiccata: by road past Villa O'Higgins at Río Mayer (JL 27089), Lago Quetru (JL 27864A).
Notes: new to Capitán Prat.

Didymodon australasiae (Hook. & Grev.) R.H.Zander
On soil of *Nothofagus dombeyi* forests; alt. 50-160 m a.s.l.; rare in the studied area.
Distribution: a sub-cosmopolitan taxon widely distributed throughout the Americas, also present in Europe, southern Africa, New Zealand and Australia (Zander 2007), distributed in Chile between Parinacota and Última Esperanza provinces (Müller 2009a).
Exsiccata: Playa Vidal at Lago Cochrane (JL 26675), balsa Baker (JL 26682 p.p. + *Leptobryum pyriforme*).
Notes: new to Capitán Prat.

Didymodon fuscus (Müll.Hal.) J.A.Jiménez & M.J.Cano
On soil or rocks inside *Nothofagus dombeyi* forests, or in open shrubland with scattered trees, or on rocks in dry steppe; alt. 50-400 m a.s.l.; rare in the studied area.
Distribution: a southern South American endemic species (Jiménez and Cano 2006) previously known in Chile between Elqui and Aisén provinces (Müller 2009a) and in the Argentinian Province of Chubut (Matteri 2003), so these records represent and extension of the southern distribution limit of the species.
Exsiccata: Tamango National Reserve (JL 26625), balsa Baker (JL 26684, JL 26721), Estancia Chacabuco (JL 26939).
Notes: new to Capitán Prat.

Gymnostomum calcareum Nees & Hornsch.
On shaded, irrigated rock face next to the river; alt. 150 m a.s.l.; rare in the studied area.
Distribution: a sub-cosmopolitan species recorded from North and South America, Europe, Asia, Azores and Canary Is., Reunion Is., New Zealand and Australia (Zander et al. 2007), previously known in Chile between Valparaíso and General Carrera provinces, and in Juan Fernández Is. (Müller 2009a), so this record represents an extension in the southern distribution limit of the species in Chile.
Exsiccata: Río Baker, Pasarela El Manzano (JL 26746).
Notes: new to Capitán Prat.

Hennediella antarctica (Ångström) Ochyra & Matteri
On dry soil among grasses and shrubs; alt. 600 m a.s.l.; rare in the studied area.
Distribution: a south-temperate species growing in southern South America, South Georgia, maritime and continental Antarctica, Kerguelen Is., Crozet Is., Heard Is., and New Zealand (Ochyra et al. 2008), apparently introduced in the British Is. (Blockeel 1990), previously known in Chile only from Magallanes Region (Müller 2009a), so this record represents an extension in the northern distribution limit of the species in Chile.
Exsiccata: Estancia Chacabuco (JL 26951).
Notes: new to Capitán Prat and to Aisén Region.

Hennediella arenae (Besch.) R.H.Zander
On dry soil inside *Nothofagus dombeyi* forests; alt. 50-70 m a.s.l.; rare in the studied area.
Distribution: a south-temperate species native to southern South America, New Zealand, Auckland Is.,

Crozet Is., Kerguelen Is., Marion Is., South Georgia and Tristan da Cunha (Cano 2008), distributed in Chile between Ñuble Province and Tierra del Fuego (Müller 2009a).
Exsiccata: balsa Baker (JL 26720), pasarela Río Ñadis (JL 26788), Route 7 south of Cochrane km 90 (JL 27930).
Notes: new to Capitán Prat.

Hennediella heimii (Hedw.) R.H.Zander
On dry soil among grasses and shrubs in steppe or on *Nothofagus dombeyi* forests floor; alt. 160-670 m a.s.l.; rare in the studied area.
Distribution: a bipolar species widely distributed in southern South America, temperate Asia, Europe, Iceland, North America, Australia, New Zealand, Antarctic Peninsula and several sub-Antarctic islands, including Campbell, Falkland, Kerguelen, South Orkneyand South Shetland Is. (Cano 2008), known in Chile between Parinacota Province to Cape Horn (Müller 2009a).
Exsiccata: Playa Vidal at Lago Cochrane (JL 26671), Estancia Chacabuco (JL 26956).
Notes: new to Capitán Prat and to Aisén Region.

Hymenostylium recurvirostrum (Hedw.) Dixon
On rocks by the lake; alt. 160 m a.s.l.; rare in the studied area.
Distribution: a sub-cosmopolitan species native to North, Central and South America, Europe, Asia, Africa and Australasia (Zander 1977), distributed in Chile between Aisén and Tierra del Fuego provinces (Müller 2009a).
Exsiccata: Playa Vidal at Lago Cochrane (JL 26668).
Notes: new to Capitán Prat.

Leptodontium longicaule Mitt. var. ***microruncinatum*** (Dusén) R.H.Zander
Among lawn grasses in human disturbed place; alt. 20 m a.s.l.; very rare in the studied area.
Distribution: an Andean-south-temperate species extending north along the Andes to Bolivia, Peru, Ecuador and Venezuela, and in Tristan da Cunha, Gough Is., Crozet Is., Reunion, and south and central-east Africa (Zander 1972), distributed in Chile between Limarí and Magallanes provinces and in Juan Fernández Is. (Müller 2009a).
Exsiccata: Puerto Yungay (JL 26513).
Notes: new to Capitán Prat and to Aisén Region.

Pseudocrossidium crinitum (Schultz) R.H.Zander
On rocks or soil inside dry deciduous or evergreen *Nothofagus* forests, *Mulinum spinosum* shrublands or steppes; alt. 50-400 m a.s.l.; very common in the studied area.
Distribution: an essentially south-temperate species native to southern South America, southern Africa and Australasia, with disjunct populations in Mexico and southwestern United States (Zander 1993), distributed in Chile between Elqui and Magallanes provinces (Müller 2009a).
Exsiccata: Tamango National Reserve (JL 26608, JL 26614C + *Orthotrichum rupestre*, JL 26617A), balsa Baker (JL 26737b + *Syntrichia anderssonii*), Estancia Chacabuco (JL 26936), road S of Cochrane front of Laguna Esmeralda (JL 27673, JL 27677, JL 27678B), Lago Chacabuco (JL 27698, JL 27722A + *Orthotrichum rupestre*, JL 27728), pasarela Ñadis (JL 27746, JL 27747).
Notes: new to Capitán Prat.

Syntrichia anderssonii (Ångström) R.H.Zander
On rocks or more rarely on base of trees inside deciduous *Nothofagus pumilio* forests, seldom on soil in forest floor; alt. 100-400 m a.s.l.; very common in the studied area.
Distribution: a south-temperate species native to Australia, New Zealand, South Georgia, Macquire Is.,

Crozet Is., Kerguelen (Lightowlers 1986), SW Argentina and the Falkland Is. (Matteri 2003), distributed in Chile between Elqui and Magallanes provinces (Müller 2009a).
Exsiccata: Route 7 south of Cochrane km 10 (JL 26382), Route 7 south of Cochrane km 26 (JL 26398B), Tamango National Reserve (JL 26632, JL 26651), balsa Baker (JL 26688, JL 26694, JL 26724b + *Hypnum cupressiforme* var. *filiforme*, JL 26737 p.p. + *Pseudocrossidium crinitum*), Río Bravo (JL 27011), road S of Cochrane front of Laguna Esmeralda (JL 27678A), mirador Lago Chacabuco (JL 27693A + *Acrocladium auriculatum* + *Cratoneuropsis relaxa* subsp. *minor* + *Polytrichum juniperinum*, JL 27703), pasarela Río Ñadis (JL 27753B + *Brachytheciastrum paradoxum*).
Notes: first reported for the province by Skottsberg (1916) as *Tortula andersonii* Ångström from W Lago O'Higgins (CS 327), and later mentioned by Donat (1936b) as growing at NW Lago O'Higgins (in text).

Syntrichia costesii (Thér.) R.H.Zander
On base of old growth *Nothofagus* trees in *N. antarctica* open forest with cattle grazing; alt. 70 m a.s.l.; rare in the studied area.
Distribution: a Chilean endemic taxon distributed between Valparaíso and Magallanes provinces (Müller 2009a).
Exsiccata: Pasarela El Salto (JL 27653).
Notes: new to Capitán Prat and to Aisén Region.

Syntrichia epilosa (Broth. ex Dusén) R.H.Zander
On rocks in dry steppe and dry shrubland; alt. 190-680 m a.s.l.; rare in the studied area.
Distribution: a Chilean endemic species previously known only from the central part of the country, between Valparaíso and Arauco provinces (Müller 2009a), so these records represent a considerable extension in the southern distribution limit of the taxon.
Exsiccata: Tamango National Reserve (JL 26613), Estancia Chacabuco (JL 26962A).
Notes: new to Capitán Prat and to Aisén Region.

Syntrichia fragilis (Taylor) Ochyra
On rocks and soil in dry steppe to sub-steppe habitats; alt. 160-670 m a.s.l.; common in steppes of the studied area, rare elsewhere.
Distribution: a sub-cosmopolitan species widely distributed throughout the Americas, Africa, Macaronesia, Europe and southern Asia (Gallego 2005), previously known in Chile from a few collections in the north and central parts of the country, between Parinacota and Valparaíso provinces (Müller 2009a), so this record represents a considerable extension in the southern distribution limit of the taxon.
Exsiccata: Playa Vidal at Lago Cochrane (JL 26667B + *Zygodon hookeri* var. *hookeri*), Estancia Chacabuco (JL 26932, JL 26934, JL 26958).
Notes: new to Capitán Prat and to Aisén Region.

Syntrichia glacialis (Kunze ex Müll.Hal.) R.H.Zander
On rocks, fallen trunks or epiphytic on *Nothofagus antarctica*, in both open and shady conditions; alt. 5-350 m a.s.l.; common in the studied area.
Distribution: a poorly understood southern South American endemic taxon described from Antuco, Bío-Bío Province, as *Barbula glacialis* Kunze ex Müll.Hal. (Müller 1849), and so far recorded in Chile between Ñuble and Magallanes provinces (TROPICOS 2016).
Exsiccata: Tamango National Reserve (JL 26659), Caleta Tortel (JL 26922), by road past Villa O'Higgins at Río Mayer (JL 27084A, JL 27093), Route 7 south of Cochrane km 90 (JL 27942, JL

27959b + *Neckera scabridens*).
Notes: new to Capitán Prat and to Aisén Region. This taxon has been recently synonymyzed by Müller (2009b) under *S. anderssonii*, and its distribution has been merged with the distribution of the latter in his recent checklist (Müller 2009a). However, *S. glacialis* differs from *S. anderssonii* in a number of morphological characters: (i) the papillosity of the dorsal part of the costa reaches the base of the costa in *S. glacialis* and only cover the distal half in *S. anderssonii*; (ii) *S. anderssonii* is synoicous whereas *S. glacialis* is dioicous; (iii) *S. glacialis* often has smaller laminal cells than *S. anderssonii*; and (iv) *S. glacialis* has margins recurved to the distal third of the leaves, whereas *S. anderssonii* only until the midleaf (M. Gallego, pers. comm.).

Syntrichia lithophila (Dusén) Ochyra & R.H.Zander
On rocks in dry steppe habitats; alt. 400-680 m a.s.l.; common in dry areas in the studied area.
Distribution: a south-temperate species present in southern South America, where it has been known also as *Syntrichia pygmaea* (Dusén) R.H.Zander (Matteri 2003) and *Sarconeuron glacialis* (Müll.Hal.) Cardot & Bryhn (Matteri 1982), and in New Zealand, where it has been known as *Tortula abruptinervis* Dixon (Zander 1993; Ochyra and Zander 2007; Ochyra et al. 2008), distributed in southern Chile between Aisén and Tierra del Fuego provinces (Müller 2009a).
Exsiccata: Estancia Chacabuco (JL 26937, JL 26938, JL 26960, JL 26964B).
Notes: new to Capitán Prat. Ochyra et al. (2008) has recently demonstrated that the southern South American records of *Syntrichia sarconeurum* Ochyra & R.H.Zander (≡*Sarconeuron glacialis, cf.* Matteri 1982) rather correspond to *S. lithophila*. The very small plants with lingulate leaves abruptly contracted into a caducous propaguliferous point are diagnostic for this species.

Syntrichia magellanica (Mont.) R.H.Zander
On dry soil in steppe habitats or dry *Nothofagus antarctica* shrublands; alt. 370-670 m a.s.l.; common in dry places in the studied area.
Distribution: a southern South American-Antarctic species distributed in southern South America, the Falkland Is. (Matteri 2003), and in Kerguelen Is., South Georgia and Antarctica (Ochyra et al. 2008), reported in Chile between Coihaique and Tierra del Fuego provinces (Müller 2009a).
Exsiccata: Route 7 south of Cochrane km 40 (JL 26406), Estancia Chacabuco (JL 26940, JL 26953, JL 26955).
Notes: new to Capitán Prat.

Syntrichia princeps (De Not.) Mitt.
On dry soil in *Mulinum spinosum* shrublands or on rocks in open sub-steppe habitats; alt. 160-380 m a.s.l.; rare in dry areas in the studied area.
Distribution: a widely distributed species in North, Central and South America, throughout Africa, Mediterranean Europe, Macaronesia, Scandinavia, the Middle East, North and South Asia, Australia, New Zealand and Oceania (Gallego 2005; Ochyra et al. 2008), reported in Chile from Valparaíso Province to Cape Horn (Müller 2009a).
Exsiccata: Playa Vidal at Lago Cochrane (JL 26665), Río Baker, pasarela El Manzano (JL 26745), Estancia Chacabuco (JL 26962B).
Notes: new to Capitán Prat. Although the taxonomic studies of this taxon are still in progress (M. Gallego, pers. comm.), specially its relationship with closely related species like *Syntrichia magellanica* and *S. antarctica*, this taxon is here accepted based in the leaf costa anatomy with hydroids.

Syntrichia pseudorobusta (Dusén) R.H.Zander
On wet soil in river bank inside *Nothofagus dombeyi* forest; alt. 70 m a.s.l.; rare in evergreen forests in

the studied area.
Distribution: a southern South American endemic taxon present in Chubut Province in SW Argentina (Matteri 2003) and known previously in Chile from Talca to Aisén provinces (Müller 2009a), so the present record extends its distribution to the south.
Exsiccata: Pasarela Río Ñadis (JL 26790).
Notes: new to Capitán Prat.

Syntrichia ruralis (Hedw.) F.Weber & D.Mohr
≡*Tortula ruralis* (Hedw.) G.Gaertn., B.Mey. & Scherb.
On soil or rotten logs inside deciduous *Nothofagus antarctica* forests or open evergreen *N. dombeyi* forests; alt. 70-300 m a.s.l.; rare in the studied area.
Distribution: a sub-cosmopolitan species known from NW South America, Central and North America, throughout Africa, Macaronesia, Europe and Asia (Gallego et al. 2002), previously known in Chile from a few collections from Talca Province in central Chile and from Magallanes and Última Esperanza provinces in the far south (Müller 2009a).
Exsiccata: Route 7 south of Cochrane km 10 (JL 26384), pasarela El Salto (JL 27637).
Notes: new to Capitán Prat and to Aisén Region.

Syntrichia scabrella (Dusén) R.H.Zander
On soil inside evergreen *Nothofagus dombeyi* forest; alt. 100 m a.s.l.; rare in the studied area.
Distribution: a Chilean endemic taxon previously known only from central Chile between Colchagua and Concepción provinces (Dusén 1906; Müller 2009a), so the present record represents a significative extension of its distribution limit to the south.
Exsiccata: road between Cochrane and Tortel (JL 26430).
Notes: new to Capitán Prat, to Aisén Region, and to Patagonia.

Tortella knightii (Mitt.) Broth.
On soil inside *Nothofagus dombeyi* evergreen forest; alt. 340 m a.s.l.; extremely rare in the studied area.
Distribution: a south-temperate species present in Tierra del Fuego, New Zealand, Tasmania, and SE Australia (Eckel 1997), previously known in southern Chile only from Antártica Chilena Province (Müller 2009a; Eckel 1997), so this record represents an extension of its northern distribution limit in South America.
Exsiccata: Lago Chacabuco (JL 27731B + *Cratoneuropsis relaxa* subsp. *minor*).
Notes: new to Capitán Prat and to Aisén Region.

Tortula atrovirens (Sm.) Lindb.
On dry soil in steppe with dominant *Mulinum spinosum*; alt. 400 m a.s.l.; rare in the studied area.
Distribution: a sub-cosmopolitan species known from Argentina, Uruguay, Bolivia, North America, Africa, Australia and New Zealand (Cano and Gallego 2008), previously reported in Chile only from the north and central zone, between Antofagasta and Talca provinces (Müller 2009a), so this record represents a considerable extension of its southernmost distribution limit.
Exsiccata: Estancia Chacabuco (JL 26932).
Notes: new to Capitán Prat, to Aisén Region, and to Patagonia.

Subclass Bryidae Engl.
Order Hedwigiales Ochyra
Family Hedwigiaceae Schimp.

Hedwigia ciliata Hedw. var. ***nivalis*** Hampe (Figure 4D)
On acidic rocks in open areas and inside deciduous *Nothofagus pumilio* forests; alt. 190-400 m a.s.l.; rare but locally common in the studied area.
Distribution: this taxon has a South American distribution, being reported previously from Venezuela to Córdoba in central Argentina, the latter being its southernmost known occurrence limit prior to this finding (Biasuso 2007).
Exsiccata: Laguna Esperanza (JL 27680A), Lago Chacabuco (JL 27702A), Route 7 south of Cochrane km 26 (JL 26394), Tamango National Reserve (JL 26612).
Notes: these records were recently published as an addition to the moss flora of Chile in another contribution based on these same collections (Ellis et al. 2014).

Family Rhacocarpaceae Kindb.

Pararhacocarpus patagonicus (Broth.) J.-P.Frahm
On bare peat or rock outcrops in Magellanic tundra habitats; alt. 100-450 m a.s.l.; rare in the studied area.
Distribution: an endemic taxon of southern Chile (Frahm 1996), known between Valdivia Province and Cape Horn (Müller 2009a).
Exsiccata: Caleta Tortel (JL 26907, JL 26913), Villa O'Higgins (JL 27069).
Notes: new to Capitán Prat.

Rhacocarpus purpurascens (Brid.) Paris
On seeping rock outcrops in Magellanic tundra environments; alt. 15-400 m a.s.l.; common in tundra habitats in the studied area.
Distribution: a pantropical species extending into the temperate areas of the southern Hemisphere, recorded for the tropical Andes extending into central America and Mexico, Falkland Islands, tropical Africa, Madagascar, South Africa, New Guinea, Borneo, Celebes, Asutralia, New Zealand, Campbell Islands, and southern South America (Frahm 1996), reported in Chile between Valdivia Province and Cape Horn, and also in Juan Fernández (Müller 2009a).
Exsiccata: Río Bravo (JL 27882), Caleta Tortel (JL 26873, JL 26901), Villa O'Higgins (JL 27077), cuesta Mirador Baker (JL 26505).
Notes: new to Capitán Prat.

Order Bartramiales D.Quandt, N.E.Bell & Stech
Family Bartramiaceae Schwägr.

Bartramia ithyphylloides Schimp. ex Müll.Hal.
On rocks, decaying wood or soil in evergreen mixed and *Nothofagus dombeyi* forests; alt. 50-400 m a.s.l.; common in the studied area.
Distribution: an endemic species of southern Chile, also present in Juan Fernández (Fransén 2004a), recorded for Chile between Cordillera Province and Cape Horn (Müller 2009a).
Exsiccata: along Route 7 south of Cochrane km 90 (JL 26434), road S of Cochrane at Tortel/Yungay fork (JL 26493), balsa Baker (JL 26713, JL 26740, JL 26716 p.p. + *Didymodon* sp.), lago Chacabuco (JL 27732).
Notes: new to Capitán Prat.

Bartramia mossmaniana Müll.Hal.
On soil and rocks in different kinds of forests, seldom epiphytic on *Podocarpus nubigena*; alt. 50-300 m a.s.l.; very common in the studied area.

Distribution: a species growing in Australasia and southern South America (Fransén 2004b), distributed in Chile between Ñuble Province and Cape Horn (Müller 2009a).
Exsiccata: Laguna Esmeralda (JL 27671), Lago Chacabuco (JL 27692), pasarela Ñadis (JL 27748, AM 1001), Lago Quetru (JL 27795, JL 27117), along Route 7 south of Cochrane km 90 (JL 27961, JL 26383), Río Bravo (JL 26973), Río Carrera (JL 26595), Tamango National Reserve (JL 26638), balsa Baker (JL 26718b + *Bartramia stricta*), Río Barrancoso (AM 994, AM 995), Lago Chico (ET 148a + *Lepyrodon lagurus* + *L. patagonicus* + *Hygroamblystegium chilense* + *Hypnum skottsbergii* + *Sanionia uncinata*).
Notes: previously reported by Skottsberg (1916) from NE Lago O'Higgins (CS 644).

Bartramia patens Brid.
On soil in evergreen *Nothofagus dombeyi* forests; alt. 50-200 m a.s.l.; rare in the studied area.
Distribution: a sub-Antarctic species growing in southern South America, Juan Fernández, Falkland Is., South Georgia, Prince Edward Is., Kerguelen, Heard Is. and Antarctica (Fransén 2004a, as *B. ithyphilla* subsp. *patens;* Ochyra et al. 2008), recorded in Chile between Aisén Province and Cape Horn (Müller 2009a).
Exsiccata: road to Puerto Yungay before Vagabundo (JL 26965A2 + *Distichium capillaceum*).
Notes: previously reported by Skottsberg (1916) from NE Lago O'Higgins (CS 647).

Bartramia robusta Hook.f. & Wilson
On dry soil next to vehicular road; alt. 50 m a.s.l.; rare in the studied area.
Distribution: a circumsubantarctic taxon growing in southern South America, New Zealand, Australia, Kerguelen Is., and Heard Is. (Matteri 1985, as *B. patens* var. *robusta*), distributed in Chile between Aisén Province and Cape Horn (Müller 2009a).
Exsiccata: along Route 7 south of Cochrane km 90 (JL 27932).
Notes: new to Capitán Prat.

Bartramia stricta Brid.
On soil in evergreen *Nothofagus dombeyi* forests, on dry soil in steppes, and on rocks in dry shrublands; alt. 60-200 m a.s.l.; common in the studied area.
Distribution: a wide-spread taxon growing in western North America, Mediterranean Europe and Africa, SE Asia, tropical Andes and southern South America (Matteri 1985; Smith 2004), recorded for Chile between Limarí and Magallanes provinces (Müller 2009a).
Exsiccata: pasarela El Salto (JL 27636A + *Orthotrichum rupestre*), pasarela Ñadis (JL 27752), Tamango National Reserve (JL 26617B, JL 26622, JL 26653, JL 26628 p.p. + *Bryum australe* + *B. canariense*), balsa Baker (JL 26718 p.p. + *Bartramia mossmaniana*), Lago Vargas (ET 86b + *Encalypta ciliata*), Estancia Chacabuco (JL 26931, JL 26935).
Notes: new to Capitán Prat.

Breutelia angustiretis E.B.Bartram
On soil banks in evergreen *Nothofagus dombeyi* forests or in open areas next to vehicular roads; alt. 210-350 m a.s.l.; not common in the studied area.
Distribution: an endemic species of southern South America (Matteri 1973, 1985), reported in Chile between Ñuble Province and Cape Horn (Müller 2009a).
Exsiccata: Villa O'Higgins (JL 27064), Río Barrancoso (AM 987).
Notes: new to Capitán Prat.

Breutelia aureola (Besch. ex Müll.Hal.) Besch.
On soil banks next to river; alt. 50 m a.s.l.; very rare in the studied area.

Distribution: an endemic taxon of southern South America (Matteri 1973, 1985), reported in Chile between Cautín Province and Cape Horn (Müller 2009a).
Exsiccata: road S of Cochrane at Tortel/Yungay fork (JL 26472).
Notes: new to Capitán Prat.

Breutelia dumosa Mitt.
On soil in the ground of very wet lowland forests and in soil banks next to vehicular roads; alt. 5-130 m a.s.l.; common in the studied area.
Distribution: a sub-antarctic taxon recorded for southern South America, Kerguelen and Marion Islands (Matteri 1973, 1985), reported in Chile between Concepción Province and Cape Horn (Müller 2009a).
Exsiccata: Lago Quetru (JL 27772, JL 27785), Lago Leal (JL 27809), Río Bravo (JL 26967, JL 27004), road S of Cochrane at Tortel/Yungay fork (JL 26473), Puerto Yungay (JL 26538), Fiordo Mitchell (JL 26564), Río Pascua (AJ 107F).
Notes: new to Capitán Prat.

Breutelia integrifolia (Taylor) A.Jaeger
On wet soil or seeping rocks in open areas, seldom on decaying wood; alt. 40-450 m a.s.l.; very common in the studied area.
Distribution: a South American-subantarctic taxon recorded for the tropical Andes, southern Chile and Argentina, and Tristan da Cunha, Kerguelen, Cozet, and Marion Islands (Matteri 1973, 1985), distributed in Chile between Ñuble Province and Cape Horn (Müller 2009a).
Exsiccata: Route 7 around Los Mellizos (JL 27761), Villa O'Higgins (JL 27073, JL 27084B, JL 27086), Route 7 south of Cochrane past Lago Vargas fork (JL 26440), Route 7 south of Cochrane km 30 (JL 26392).
Notes: first reported for the province by Skottsberg (1916) from NE Lago O'Higgins (CS 686).

Breutelia plicata Mitt.
On the forest floor in very wet lowland forest; alt. 50-130 m a.s.l.; rare in the studied area.
Distribution: an endemic taxon of southern Chile and Argentina (Matteri 1973, 1985), reported in Chile between Cautín Province and Cape Horn (Müller 2009a).
Exsiccata: Lago Quetru (JL 27803, JL 27848A), Río Bravo (JL 27032).
Notes: new to Capitán Prat.

Breutelia subplicata Broth.
On wet soil in evergreen forests, or on peat in Magellanic moorland; alt. 30-210 m a.s.l.; common in the studied area.
Distribution: an endemic taxon of southern South America and Juan Fernández (Matteri 1973, 1985), reported in Chile between Talca and Magallanes provinces (Müller 2009a).
Exsiccata: Lago Quetru (JL 27875), pasarela Lago Vargas (JL 26804), Caleta Tortel (JL 26885), Río Bravo (JL 27014).
Notes: new to Capitán Prat.

Conostomum pentastichum (Brid.) Lindb.
On rock outcrops and rock crevices in open Magellanic moorland; alt. 20-400 m a.s.l.; common in the studied area.
Distribution: a widely distributed circumsubantarctic species, extending into the tropical Andes and into New Guinea in Australasia, present in New Zealand, Tasmania, most of the subantarctic islands and Antarctica (Matteri 1985; Ochyra et al. 2008), recorded in Chile from Malleco Province to Cape Horn (Müller 2009a).

Exsiccata: Caleta Tortel (JL 26874), Río Carrera (JL 26457), road S of Cochrane past Tortel/Yungay fork (JL 26841), Villa O'Higgins (JL 27062, JL 27079).
Notes: new to Capitán Prat.

Philonotis brevifolia Herzog
On stony soil in disturbed area; alt. 20 m a.s.l.; extremely rare in the studied area.
Distribution: endemic from southern Chile (Jimenez et al. 2016), so far only known from two collections from Aisén region, the type material and the specimen here reported (Jimenez et al. 2016).
Exsiccata: Puerto Yungay (JL 26508).
Notes: first reported for the province by Jimenez et al. (2016); this taxon has been considered a synonym of *Philonotis krausei* (Seki 1974), but it differs in a number of morphological traits (cf. Jimenez et al. 2016).

Philonotis esquelensis Matteri
On soil in dry shrubland; alt. 200 m a.s.l.; rare in the studied area.
Distribution: an endemic taxon restricted to the southern Andes, recently reported for Chile between Melipilla Province and Capitán Prat (Jimenez et al. 2014), being the Capitán Prat record based on the same specimen here reported.
Exsiccata: Reserva Nacional Tamango (JL 26624).
Notes: first reported for the province by Jimenez et al. (2014) based on this same voucher.

Philonotis krausei (Müll.Hal.) Broth.
On soil on waterfall banks or seldom on bases of *Berberis* shrubs; alt. 70-380 m a.s.l.; rare in the studied area.
Distribution: an endemic species of the southern Chilean Andes, with a single record for Argentina, and common in the Juan Fernández Islands (Matteri 1968), previously reported for Chile between Petorca and Aisén provinces (Müller 2009a) so these records represents a southern limit range extension.
Exsiccata: Route 7 south of Cochrane, km 20 (JL 26389, JL 26391), Lago Quetru (JL 27122A).
Notes: new to Capitán Prat.

Philonotis polymorpha (Müll.Hal.) Kindb.
=*Philonotis acicularis* (Müll.Hal.) Kindb.
On soil in river bank; alt. 200 m a.s.l.; rare in the studied area.
Distribution: a circumsubantarctic taxon reported for the South Shetland Is., South Georgia, Prince Edward and Marion Is., Heard Is., Crozet Is, Île de la Possession, Kerguelen, Macquarie Is. and southern South America (Ochyra et al. 2008; Bednarek-Ochyra 2014), reported for Chile between Choapa Province and Tierra del Fuego (Müller 2009a).
Exsiccata: Playa Vidal at Lago Cochrane (JL 26672).
Notes: new to Capitán Prat and to Aisén Region.

Philonotis scabrifolia (Hook.f. & Wilson) Braithw.
On soil banks, in the forest floor or on base of trees inside evergreen and deciduous *Nothofagus* forests; alt. 30-350 m a.s.l.; common in the studied area.
Distribution: a wide-spread Southern Hemisphere species, distributed in New Zealand, Tasmania, South Africa, Auckland Is., Marion Is., Kerguelen, Tristan de Cunha, South Georgia, and the southern and tropical Andes, extending north even to Mexico (Matteri 1968, 1985); reported in Chile between Talca Province and Cape Horn (Müller 2009a).
Exsiccata: pasarela Ñadis (JL 27741B + *Chrysoblastella chilensis*), Laguna Esmeralda (JL 27663B), Lago Quetru (JL 27852), Villa O'Higgins (JL 27094), along Route 7 south of Cochrane km 90 (JL

26428), puente El Manzano (ET & RR s.n.), Colonia Norte (ET 14).
Notes: new to Capitán Prat and to Aisén Region.

Philonotis vagans (Hook.f. & Wilson) Mitt.
On wet soil or on river banks in evergreen or deciduous *Nothofagus* forests; alt. 130-400 m a.s.l.; not common in the studied area.
Distribution: an almost endemic southern South American species, also growing in Juan Fernández, the Falkland Islands, and extending its distribution range to Tristan da Cunha (Matteri 1968, 1985), previously reported for Chile between Maipo Province and Cape Horn (Müller 2009a).
Exsiccata: Río Bravo (JL 27022), Route 7 south of Cochrane km 26 (JL 26405), balsa Baker (JL 26733).
Notes: first reported for the province by Donat (1936a) as *Goniobryum reticulatum* (Hook.f. & Wilson) Lindb. var. *evanidum* Cardot & Brotherus from western Lago O'Higgins (AD 13).

Order Splachnales
Family Splachnaceae

Tayloria dubyi Broth.
On open peaty soil on lake shore; alt. 50-70 m a.s.l.; rare in the studied area, only found in peatlands.
Distribution: a southern South American endemic (Koponen 1982; Jofre et al. 2010, 2011), distributed in Chile from Aisén Province to Cape Horn (Müller 2009a), and in Argentinian side of Tierra del Fuego (Matteri 2003).
Exsiccata: Lago Quetru (JL 27124B + *Tetraplodon fuegianus*, JL 27769).
Notes: new to Capitán Prat.

Tayloria stenophysata (Herzog) A.K.Kop.
On soil in tundras; alt. 20 m a.s.l.; rare in the studied area.
Distribution: a southern South American endemic (Koponen 1982; Jofre et al. 2010) distributed in Chile between Valdivia and Magallanes provinces (Müller 2009a), and in the Argentinian province of Santa Cruz (Matteri 2003).
Exsiccata: Fiordo Mitchell (AJ 311).
Notes: new to Capitán Prat and to Aisén Region. This species looks superficially very similar to *Tetraplodon fuegianus*, but it differs from the latter by the basal exothecial cells isodiametric and with vertical walls not thickened.

Tayloria magellanica (Brid.) Mitt.
On soil in the forest floor; alt. 400 m a.s.l.; rare in the studied area.
Distribution: an Andean species growing all the way from the Colombian Andes to Cape Horn (Churchill and Linares 1995; Koponen 1982; Matteri 2003; Menzel 1992), previously reported in Chile only for Magallanes region (Müller 2009a), so this record represents an extension to the north in the distribution limit of the taxon in Chile.
Exsiccata: Laguna Caiquenes, between Vagabundo and Yungay (AJ 6A).
Notes: new to Capitán Prat and to Aisén Region.

Tetraplodon fuegianus Besch. (Figure 3E)
On soil in open tundras or other exposed soil, frequently on animal excrements; alt. 5-90 m a.s.l.; common in the studied area.
Distribution: a southern South American endemic (Goffinet et al. 2010), reported in Chile between Valdivia Province and Cape Horn (Müller 2009a).

Exsiccata: Puerto Yungay (JL 26536), Caleta Tortel (JL 26876), Lago Quetru (JL 27124A + *Tayloria dubyi*, JL 27768), Lago Vargas (AJ 353).
Notes: new to Capitán Prat. Commonly treated as *Tetraplodon mnioides* (e.g. Müller 2009a), although molecular studies support it as a separate species (Goffinet et al. 2010).

Family Meesiaceae Schimp.

Leptobryum pyriforme (Hedw.) Wilson
On soil banks in disturbed *Nothofagus dombeyi* forests; alt. 50-160 m a.s.l.; not common in the studied area.
Distribution: a cosmopolitan species (Arts 2001) distributed in Chile between Limarí and Magallanes provinces, also in Easter Island (Müller 2009a).
Exsiccata: Playa Vidal at Lago Cochrane (JL 26674), balsa Baker (JL 26682 p.p. + *Didymodon australasiae*).
Notes: new to Capitán Prat.

Order Bryales Limpr.
Family Bryaceae Schwägr.

Bryum algovicum Sendtn. ex Müll.Hal.
On fallen trunk in deciduous *Nothofagus antarctica* forest; alt. 70 m a.s.l.; rare in the studied area.
Distribution: a bipolar species (Ochi 1990) previously known in Chile only from Magallanes and Última Esperanza provinces in Magallanes region (Müller 2009a), so this record represents an extension of the northern limit of the distribution of this taxon in Chile.
Exsiccata: Pasarela El Salto (JL 27643).
Notes: new to Capitán Prat and to Aisén Region.

Bryum archangelicum Bruch & Schimp.
On soil in disturbed deciduous *Nothofagus* forests or at the forest edge; alt. 70-360 m a.s.l.; rare in the studied area.
Distribution: a mostly bipolar species widely distributed in the Arctic, extending southwards to California, Mediterranean Europe, northern Africa, the Middle East, the Caucasus and Japan, also in the Neotropics and in some subantarctic islands and Antarctica (Ochyra et al. 2008), distributed in Chile between Santiago Province and Cape Horn (Müller 2009a).
Exsiccata: pasarela El Salto (JL 27655), mirador Lago Chacabuco (JL 27691B + *Ceratodon purpureus*).
Notes: new to Capitán Prat and to Aisén Region.

Bryum australe Hampe
On open soil in dry steppe; alt. 200-380 m a.s.l.; rare in the studied area.
Distribution: a south-temperate to south-subtropical species distributed in southern South America, NE Argentina (Matteri 2003), Uruguay, New Guinea (Ochi 1982), Australia, Tasmania (Streimann and Curnow 1989) and New Zealand (Ochi 1970), reported in Chile between San Antonio and Magallanes provinces (Müller 2009a).
Exsiccata: Reserva Nacional Tamango (JL 26628B2 + *Bryum canariense* + *Bartramia stricta*), Puente El Manzano (RR & ER s.n.).
Notes: new to Capitán Prat. The triangular to ovate, not bordered leaves, ended in a long excurrent costa, with revolute margins throughout, and the lax leaf areolation are diagnostic for this taxon. The very long-necked capsules in these specimens are different from typical *B. australe* (J. Spence, pers.

comm.).

Bryum billarderi Schwägr. (Figure 4E)
On soil or fallen logs in open evergreen *Nothofagus dombeyi* or deciduous *N. pumilio* forests, or on open soil in stony lake banks; alt. 30-300 m a.s.l.; not rare in the studied area.
Distribution: a pan-tropical species extending to temperate areas in both hemispheres (Ochi 1970, 1980), previously known in Chile between Limarí and Llanquihue provinces (Müller 2009a) so these new records considerably extend its southern distributional limit to the south.
Exsiccata: pasarela Río Ñadis (JL 26799), Laguna Esmeralda (JL 27670), Lago Quetru (JL 27877).
Notes: new to Capitán Prat and to Aisén Region.

Bryum caespiticium Hedw.
On soil or rotten logs on evergreen *Nothofagus dombeyi* or deciduous *N. antarctica* forests; alt. 70-100 m a.s.l.; not common in the studied area.
Distribution: a cosmopolitan species (Ochi 1980) distributed in Chile between Aisén Province and Cape Horn (Müller 2009a).
Exsiccata: road between Cochrane and Tortel (JL 26419B + *Chrysoblastella chilensis*), Pasarela El Salto (JL 27644).
Notes: new to Capitán Prat.

Bryum canariense Brid.
On soil in river banks in evergreen *Nothofagus dombeyi* forests or in open soil in steppe habitats; alt. 100-200 m a.s.l.; rare in the studied area.
Distribution: a "Mediterranean" species known from west coastal regions of Europe, west to south coastal regions of Africa, southern South America, and west coastal regions of North America (Ochi 1980), previously reported in Chile between Marga-Marga and Valdivia provinces (Ochi 1980), so these new records extend its southern distribution limit.
Exsiccata: Reserva Nacional Tamango (JL 26628B1 + *Bryum australe* + *Bartramia stricta*), Balsa Baker (JL 26697).
Notes: new to Capitán Prat and to Aisén Region. The plants with oblong leaves crowded in a rosette, indistinctly bordered and with a short excurrent costa are diagnostic for this species. The material here reported has much narrower leaves than normal phenotypes.

Bryum capillare Hedw.
On fallen trunck inside open and disturbed *Nothofagus dombeyi* forest; alt. 300 m a.s.l.; not common in the studied area.
Distribution: a cosmopolitan species (Ochi 1980) distributed in Chile between Concepción Province and Cape Horn (Müller 2009a).
Exsiccata: on Route 7, 10 km S of Cochrane (JL 26378).
Notes: new to Capitán Prat.

Bryum clavatum (Schimp.) Müll.Hal.
Distribution: a circumsubantarctic taxon extending north along the Andes into the tropics, known from southern South America, Tristan da Cunha, Australia, New Zealand, South Georgia and the Juan Fernández Islands (Ochi 1980), reported in Chile between Valparaíso Province and Cape Horn (Müller 2009a).
Notes: Reported by Donat (1936a) as *B. cochlearifolium* Cardot & Broth. fo. *minor nom. nud.* from W side of Lago O'Higgins (AD s.n.), and by Cardot and Brotherus (1923) as the new species *B. microphyllum* Cardot & Broth. from Glaciar Schönemayer (CS 542). Both names are synonyms of *B.*

clavatum (Ochi 1980).

Bryum coronatum Schwägr.
On irrigated rock walls by a river; alt. 150 m a.s.l.; rare in the studied area.
Distribution: a pantropical species extending its distribution range into the temperate areas of both hemispheres (Ochi 1980), previously reported for Chile from some collections from Aisén Province (Seki 1974), so this record extends its known distribution in Chile to the south.
Exsiccata: pasarela El Manzano (JL 26747).
Notes: new to Capitán Prat. This collection represents an odd phenotype with huge bulbils with leafy primordia, rhizoidal tubers dark red-brown, spherical, 120-160 µm, leaves narrowly triangular, laminal cells very incrassate, costa strong and short excurrent, no capsules. This might represent an undescribed taxon, presented here as *B. coronatum* until more thorough studies are done (J. Spence, pers. comm.).

Bryum crassinervium Lorentz
On open soil, sometimes on animal dung growing together with *Tetraplodon fuegianus*; alt. 70-90 m a.s.l.; not common in the studied area.
Distribution: a species distributed in Africa, South and Southeastern Asia, Japan and at high altitudes or high latitudes in South America (Ochi 1980), previously reported in Chile from a couple of localities in Valparaíso Province and Los Ríos Region (Müller 2009a), so this new record is a considerable extension for the southern distribution limit of the species in Chile.
Exsiccata: Caleta Tortel (JL 26886A), Lago Quetru (JL 27124C).
Notes: new to Capitán Prat and to Aisén Region. Ochi (1980) sunks this taxon into synonymy of the Asian *B. paradoxum* Schwaegr., but there are morphological differences between these species so the name is retained as *B. crassinervium* pending more studies (J. Spence, pers. comm.).

Bryum dichotomum Hedw.
On peat or open soil next to peatlands; alt. 5-100 m a.s.l.; common in the studied area.
Distribution: this is a wide-spread species in temperate areas of both the northern and southern hemispheres (Ochi 1980), previously reported for Chile from Valparaíso Province to Tierra del Fuego (Müller 2009a).
Exsiccata: embarcadero Río Bravo (JL 27764), Puerto Yungay (JL 26515 p.p. + *Funaria hygrometrica*), Caleta Tortel (JL 26905).
Notes: new to Capitán Prat. These specimens represent an odd phenotype with short and thick capsules, with moderately thick apophysis, cilia rudimentary, basal membrane low, exostome teeth short, spores 20-24 um, large elongate-cylindrical bulbils with leafy primordia present, small reddish tubers on rhizoids arising from leaf axils present, leaves ovate with long-excurrent spinose/denticulate costa (J. Spence, pers. comm.).

Bryum donatii Thér.
Distribution: endemic to Capitán Prat (Ochi 1982).
Notes: this taxon is only known from the type collection made by A. Donat in January 1933 in Lago O'Higgins (AD 18), at 600 m a.s.l. in an old glaciar moraine growing together with *Epilobium glaciale*; erroneously reported for Aisén Province by Müller (2009); Ochi (1982) considers it a good species, closely related to *B. algovicum*, differing from the latter by the leaves without a distinct border, costa short excurrent, and endostome segments free from exostome and without vertical thickenings.

Bryum gemmatum Müll.Hal.
Distribution: a little known southern South American endemic taxon, known only from Tierra del Fuego and Capitán Prat Province (Müller 1885; Cardot and Brotherus 1923; Matteri and Schiavone

2002).
Notes: Reported by Cardot and Brotherus (1923) from Glaciar Schönemayer (CS 541), but not recorded for the Province in Müller's (2009a) checklist.

Bryum laevigatum Hook.f. & Wilson
On humid soil, irrigated rocks, soil banks, often associated with water courses but sometimes growing in dry conditions too, in open areas, shrublands or different types of forests; alt. 120-430 m a.s.l.; a very common and very characterisitc moss in the studied area.
Distribution: a circumsubantarctic species, getting into the tropics of South America and Australia at high elevation (Ochi 1980), reported for Chile between Bío-Bío Province and Cape Horn (Müller 2009a).
Exsiccata: road to Villa O'Higgins (JL 27065, JL 27075), Río Mayer (JL 27091B), Route 7 south of Cochrane km 26 (JL 26404), road to Tortel (JL 26433).
Notes: new to Capitán Prat.

Bryum macrophyllum Cardot & Broth.
On rocks inside *Nothofagus pumilio* forest; alt. 360 m a.s.l.; rare in the studied area.
Distribution: a species distributed in southern Chile between Chiloé and Última Esperanza provinces (Müller 2009a) as well as in the Falkland Islands (Ochi 1982).
Exsiccata: mirador Lago Chacabuco (JL 27697).
Notes: new to Capitán Prat. First reported for Chile by Ochi (1990) based on a collection from Aisén Province.

Bryum perlimbatum Cardot
On fallen trunk along trail in open and dry shrub vegetation; alt. 260 m a.s.l.; rare in dry areas in the studied region.
Distribution: a circumsubantarctic species (Ochi 1982), recorded for Chile between Limarí Province and Cape Horn (Müller 2009a).
Exsiccata: Tamango National Reserve (JL 26647).
Notes: new to Capitán Prat.

Bryum platyphyllum (Schwägr.) Müll.Hal.
On soil in river bank, open; alt. 400 m a.s.l.; rare in the studied area.
Distribution: a rare moss previously known only from central Chile and Córdoba Province in Argentina (Ochi 1982; Müller 2009a), so this record represents a remarkable extension in the southern distribution range of the taxon.
Exsiccata: balsa Baker (JL 26731).
Notes: new to Capitán Prat, to Aisén Region, and to Patagonia.

Bryum pseudotriquetrum (Hedw.) Schwägr.
On soil in evergreen *Nothofagus* forests; alt. 100-250 m a.s.l.; rare in the studied area.
Distribution: a sub-cosmopolitan species only absent from SE Asia, north and central Australia, India, and north and west Africa (Ochyra et al. 2008). In Chile it has been reported between Santiago Province and Cape Horn (Müller 2009a).
Exsiccata: balsa Baker (JL 26690).
Notes: first reported for the province by Cardot and Brotherus (1923) from Glaciar Schönemayer (CS 529), and by Donat (1936a) from Lago O'Higgins (AD s.n.); although the specimens from Lago O'Higgins and Schönemayer glacier were not observed, the specimen from balsa Baker is synoicous and might correspond to another species (J. Spence, pers. comm.).

Bryum puconense Herzog & Thér.
On soil bank inside *Nothofagus dombeyi* forest; alt. 160 m a.s.l.; rare in the studied area.
Distribution: a rare Chilean endemic, previously known only from the type material collected in Cautín Province (Ochi 1982), so this record represents the second known locality for the species and an extension in its southern distribution range.
Exsiccata: Playa Vidal at Lago Cochrane (JL 26669).
Notes: new to Capitán Prat, to Aisén Region and to Patagonia. Ochi (1982) sunks this species into the synonymy of *Bryum capillare*, but *B. puconense* is quite distinct in having large 200-300 µm orange tubers (J. Spence, pers. comm.).

Bryum subapiculatum Hampe
On open soil bank of vehicular road by a humid *Nothofagus-Podocarpus-Drymis* forest; alt. 50 m a.s.l.; rare in the studied area.
Distribution: a wide-spread moss in subtropical to temperate areas of both hemispheres (Ochi 1980), previously reported from Chile between Llanquihue and Aisén provinces., so this new record represents a southern extension of the distribution range of the taxon.
Exsiccata: Lago Quetru (JL 27767).
Notes: new to Capitán Prat. This collection represents a rare phenotype with no rhizoidal tubers.

Family Mniaceae Schwägr.

Pohlia cruda (Hedw.) Lindb.
On wet soil in evergreen *Nothofagus* forests and shrublands, or on river banks; alt. 50-360 m a.s.l.; common in the studied area.
Distribution: a bipolar species with some populations occurring at high altitude in the tropics (Ochyra et al. 2008), distributed in Chile from Chacabuco Province to Cape Horn (Müller 2009a).
Exsiccata: Tamango National Reserve (JL 26641A, JL 26618), mirador Lago Chacabuco (JL 27705), Villa O'Higgins (JL 27092), balsa Baker (JL 26677, JL 26700, JL 26686, JL 26722b + *Pohlia nutans*), puente El Manzano (ET & RR s.n).
Notes: first reported for the province by Donat (1936b), without mentioning any voucher.

Pohlia longicollis (Hedw.) Lindb.
On wet soil in evergreen *Nothofagus dombeyi* forest; alt. 60 m a.s.l.; rare in the studied area.
Distribution: this species is known from Africa, Europe, India, Japan and North America, and in South America has only been reported for the tropical Andes of Perú, Ecuador and Bolivia, above 4000 m (Suárez and Schiavone 2011), then being this the first record of the species for Chile, for southern South America, and an extension in the southern distribution range of the species.
Exsiccata: balsa Baker (JL 26725).
Notes: new to Capitán Prat, to Aisén Region, and to Chile. The glossy plants with setaceous and recurved leaf apices, and costa relatively short, are diagnostic characters for this taxon.

Pohlia nutans (Hedw.) Lindb. (Figure 3A)
On dry soil in a wide range of habitats, from evergreen forests to dry shrubland, common on soil next to trails and vehicular roads; alt. 15-300 m a.s.l.; very common in the studied area.
Distribution: a bipolar species widely distributed in the temperate and cold areas of both hemispheres, and rare at high altitudes in tropical mountains (Ochyra et al. 2008; Suárez and Schiavone 2011), known in Chile between Chacabuco Province and Cape Horn (Müller 2009a).
Exsiccata: balsa Baker (JL 26701 p.p. + *Ceratodon purpureus*, JL 26678, JL 26722 p.p. + *Pohlia cruda*,

JL 26715b + *Polytrichum piliferum*), Lago Leal (JL 27126, JL 27808), Laguna Esmeralda (JL 27668, JL 27674), pasarela Río Ñadis (JL 27754, JL 26792), Lago Quetru (JL 27773, JL 27822A), Río Baker by road between Route 7 and Tortel (JL 27890, JL 26847), Río Carrera (JL 26442A + *Leptotheca gaudichaudii*, JL 26450, JL 26444 p.p. + *Leptotheca gaudichaudii*), road S of Cochrane at Tortel/Yungay fork (JL 26484), Puerto Yungay (JL 26529), Caleta Tortel (JL 26903), Río Bravo (JL 26969), Route 7 south of Cochrane km 90 (JL 27934 p.p. + *Ceratodon purpureus*), Río El Paso (AJ 1B), Río Balboa valley (RR & ET 70-2), road to Yungay front of Laguna Caiquenes (AJ 6B), pasarela El Salto (AM 960), Río Barrancoso (AM 993).
Notes: new to Capitán Prat.

Pohlia wahlenbergii (F.Weber & D.Mohr) A.L.Andrews (Figure 4G)
On wet soil in deciduous *Nothofagus pumilio* forests, in soil banks in evergreen *N. dombeyi* forests, and seldom on decaying wood of man-made constructions or even epiphytic on *Berberis darwinii*; alt. 15-400 m a.s.l.; common in the studied area.
Distribution: a bipolar species with a few stations at high altitudes in the tropics (Ochyra et al. 2008), reported in Chile between Petorca Province and Cape Horn (Müller 2009a).
Exsiccata: Caleta Tortel (JL 26908b + *Bryum* sp., JL 26912b + *Funaria hygrometrica*, JL 27887), Tamango National Reserve (JL 26649, JL 26637), Lago Quetru (JL 27122C), Playa Vidal at Lago Cochrane (JL 26673), balsa Baker (JL 26743), Laguna Esmeralda (JL 27686).
Notes: new to Capitán Prat.

Family Leptostomataceae Schwägr.

Leptostomum menziesii R.Br.
Epiphytic in evergreen mixed forests, seldom on fallen trees or rocks; alt. 30-130 m a.s.l.; not common in the studied area.
Distribution: an endemic species of the forests of southern South America (Hyvönen 1987), distributed in Chile from Cauquenes Province to Cape Horn (Müller 2009a).
Exsiccata: Route 7 south of Cochrane km 90 (JL 27967), Río Bravo (JL 27015), Fiordo Mitchell (JL 26571 p.p. + *Lepyrodon hexastichus*).
Notes: new to Capitán Prat and to Aisén Region.

Order Orthotrichales Dixon
Family Orthotrichaceae Arn.

Macromitrium pertriste Müll.Hal.
Epiphytic on *Nothofagus dombeyi*; alt. 5 m a.s.l.; rare in the studied area.
Distribution: a little known species known as a Valdivian rainforest endemic (Müller 1898; Thériot 1934), reported for Chile from Valdivia and Chiloé provinces (Müller 2009a), being this record a southern extension in its distribution range.
Exsiccata: Caleta Tortel (JL 26925).
Notes: new to Capitán Prat and to Aisén Region.

Matteria gracillima (Besch.) Goffinet
Epiphytic on *Nothofagus dombeyi* or *Pilgerodendron uviferum* in very wet lowland mixed forests; alt. 20-130 m a.s.l.; not rare in the most wet forests in the studied area.
Distribution: a southern Chile endemic species (Matteri and Calabrese 1999), known between Valdivia and Magallanes provinces (Müller 2009a).
Exsiccata: Lago Quetru (JL 27799b + *Ulota rufula* + *Cryphaea consimilis*), Caleta Tortel (JL 26917),

Río Bravo (JL 27009), Puerto Yungay (JL 26524).
Notes: new to Capitán Prat.

Matteria papillosula (Thér.) Goffinet
Epiphytic on *Nothofagus dombeyi* in very wet lowland mixed forests; alt. 50-130 m a.s.l.; not rare in the most wet forests in the studied area.
Distribution: a southern Chile endemic species (Matteri and Calabrese 1999), known between Llanquihue and Aisén provinces (Müller 2009a), so this record represents a southern extension of its distribution range.
Exsiccata: Lago Quetru (JL 27869B, JL 27793D + *Chorisodontium dicranelltum* + *Ulota pygmaeothecia* + *U. rufula*), Caleta Tortel (JL 26918), Río Bravo (JL 27030C + *Chorisodontium dicranellatum* + *Ulota pusilla* + *Weymouthia mollis*, JL 26965B2 + *Ulota rufula*).
Notes: new to Capitán Prat. The name of this taxon has often been spelled *Matteria papillosa* (e.g. Müller 2009a), although Matteri and Calabrese (1999) present strong evidence towards the use of *M. papillosula* instead.

Orthotrichum assimile Müll.Hal.
Epiphytic on *Nothofagus antarctica* in dry forest; alt. 70-220 m a.s.l.; rare in the studied area.
Distribution: a species growing in Australia, New Zealand and southern South America (Lewinsky 1984), reported for Chile between Valparaíso and Magallanes provinces (Müller 2009a).
Exsiccata: Tamango National Reserve (JL 26633A + *Orthotrichum elegantulum* + *O. incanum* + *O. ludificans* + *O. rupestre*), pasarela Río Ñadis (JL 26793D + *Orthotrichum brotheri* + *O. hortense* +*O. incanum*).
Notes: new to Capitán Prat.

Orthotrichum brotheri Dusén ex Lewinsky
Epiphytic on *Nothofagus dombeyi*; alt. 70-110 m a.s.l.; rare in the studied area.
Distribution: an endemic taxon of Chile (Lewinsky 1987), where it has been reported from Bío-Bío Province to Cape Horn (Müller 2009a).
Exsiccata: pasarela Río Ñadis (JL 26793A + *Orthotrichum assimile* + *O. hortense* +*O. incanum*, AM 1002A + *O. elegantulum* + *O. incanum*).
Notes: new to Capitán Prat.

Orthotrichum elegantulum Schimp. ex Mitt.
Epiphytic on *Nothofagus antarctica* and on *N. dombeyi*; alt. 70-220 m a.s.l; rare in the studied area.
Distribution: an endemic species from southern Chile and Argentina (Lewinsky 1987), distributed in Chile between Valparaíso Province and Cape Horn (Müller 2009a).
Exsiccata: pasarela Río Ñadis (AM 1002B + *O. brotheri* + *O. incanum*), Tamango National Reserve (JL 26633E + *Orthotrichum assimile* + *O. incanum* + *O. ludificans* + *O. rupestre*, 26660B + *O. incanum*).
Notes: new to Capitán Prat.

Orthotrichum hortense Bosw.
Epiphytic on *Nothofagus dombeyi*; alt. 70 m a.s.l.; extremely rare in the studied area.
Distribution: a species native to Australia, New Zealand and southern South America (Lewinsky 1987), reported for Chile between Talca and Valdivia provinces (Müller 2009a), so this collection represents an extension in the southern distribution limit of the taxon.
Exsiccata: pasarela Río Ñadis (JL 26793C + *Orthotrichum assimile* + *O. brotheri* + *O. incanum*).
Notes: new to Capitán Prat and to Aisén Region.

Orthotrichum incanum Müll.Hal.
=*Orthotrichum bicolor* Thér.
Epiphytic on *Nothofagus antarctica* and on *N. dombeyi*; alt. 70-220 m a.s.l.; common in the studied area.
Distribution: a southern South American endemic taxon (Lewinsky 1987, as *O. bicolor*; Matteri 2000), reported in Chile between Valaparíso Province and Cape Horn (Müller 2009a).
Exsiccata: Tamango National Reserve (JL 26633D + *Orthotrichum assimile + O. elegantulum + O. ludificans + O. rupestre*, JL 26660A + *O. elegantulum*), pasarela Río Ñadis (JL 26793B + *Orthotrichum assimile + O. brotheri + O. hortense*, AM 1002C + *O. brotheri + O. elegantulum*), pasarela El Salto (JL 27648A + *Camptodontium cryptodon*, JL 27649).
Notes: new to Capitán Prat. This taxon was synonymyzed by Lewinsky (1987) under *O. elegantulum*, based in a very poor type specimen. This name was readdressed by Matteri (2000) who demonstrates its differences with *O. elegantulum,* and regarded it as conspecific with *O. bicolor*, for which the oldest available name is *O. incanum* (Matteri 2000).

Orthotrichum ludificans Lewinsky
Epiphytic on *Nothofagus antarctica* and on shrubs in dry forests; alt. 220-260 m a.s.l.; rare in the studied area.
Distribution: endemic from southern South America (Lewinsky 1987), reported for Chile between Bío-Bío and Última Esperanza provinces (Müller 2009a).
Exsiccata: Tamango National Reserve (JL 26633B + *Orthotrichum assimile + O. elegantulum + O. incanum + O. rupestre*, JL 26640), pasarela El Salto (JL 27647).
Notes: new to Capitán Prat.

Orthotrichum pariatum Mitt.
Epiphytic in *Azara* shrubs or on fallen logs in *Nothofagus dombeyi* or in mixed forest understorey; alt. 50-300 m a.s.l.; rare in the studied area.
Distribution: an Andean taxon that extends its distribution north up to Costa Rica (Lewinsky 1987), just recently reported for Chile from Talca and Ñuble provinces (Müller 2009a, 2009b), this record representing a great extension in its southern distribution limit and the first record for Patagonia.
Exsiccata: Route 7 south of Cochrane km 90 (JL 27937A + *Ulota rufula*), Route 7 south of Cochrane km 10 (JL 26381A, JL 26381C + *Camptodontium cryptodon*).
Notes: new to Capitán Prat, to Aisén Region, and to Patagonia.

Orthotrichum rupestre Schleich. ex Schwägr.
On rocks in *Nothofagus pumilio* forests, seldom epiphytic in *N. dombeyi*; alt. 220-400 m a.s.l.; common in the studied area.
Distribution: a cosmopolitan species (Lewinsky 1987), reported for Chile between Valparaíso Province and Cape Horn (Müller 2009a).
Exsiccata: Route 7 south of Cochrane km 26 (JL 26397), Tamango National Reserve (JL 26633C + *Orthotrichum assimile + O. elegantulum + O. incanum + O. ludificans*, JL 26614A, JL 26614D + *Pseudocrossidium crinitum*, JL 26639), pasarela El Salto (JL 27636B + *Bartramia stricta*), Laguna Esmeralda (JL 27681A + *Schistidium* sp.), Lago Chacabuco (JL 27722B + *Pseudocrossidium crinitum*).
Notes: new to Capitán Prat.

Ulota billbuckii Garilleti, Mazimpaka & F.Lara
Epiphytic on *Nothofagus dombeyi*, seldom on stony ground, in very humid lowland mixed forests; alt. 5-100 m a.s.l.; rare in the studied area.

Distribution: a recently described taxon endemic to Chile, known in the country from the Cape Horn Archipelago and from these collections from Capitán Prat Province (Garilleti et al. 2012).
Exsiccata: Caleta Tortel (JL 26884B, 26884C + *Ulota larrainii*, JL 26927b, JL 26927d + *Ulota streptodon* + *U. fuegiana* + *U. magellanica* + *U.pusilla*), Puerto Yungay (JL 26510, JL 26525), Lago Quetru (JL 27123C + *Ulota fuegiana* + *U. pusilla*).
Notes: recently described by Garilleti et al. (2012).

Ulota carinata Mitt.
Epiphytic on *Nothofagus dombeyi*; alt. 30 m a.s.l.; rare in the studied area.
Distribution: an endemic species from southern Chile (Mitten 1859; Malta 1927), distributed between Llanquihue and Chiloé provinces (Müller 2009a), so this record extends to the south the known distribution of the taxon.
Exsiccata: Río Carrera (JL 26593).
Notes: new to Capitán Prat and to Aisén Region.

Ulota fuegiana Mitt.
Epiphytic on *Baccharis* sp., *Berberis illicifolia* and *Gaultheria insana* shrubs, and on *Nothofagus dombeyi*; alt. 5-130 m a.s.l.; common in the studied area.
Distribution: a southern South American endemic species (Malta 1927), reported for Chile between Valdivia Province and Cape Horn (Müller 2009a).
Exsiccata: road S of Cochrane past Tortel/Yungay fork (JL 26840), Caleta Tortel (JL 26927F + *Ulota streptodon* + *U. billbuckii* + *U. magellanica* + *U.pusilla*), Río Bravo (JL 27033), Lago Quetru (JL 27123A + *Ulota billbucki* + *U. pusilla*, JL 27869A), Lago Leal (JL 27820A + *Porella* sp. + *Ulota rufula* + *U. luteola*).
Notes: new to Capitán Prat.

Ulota germana (Mont.) Mitt.
Epiphytic on branches of *Azara* shrubs inside *Drimys-Weinmannia-Podocarpus* lowland forest; alt. 30 m a.s.l.; rare in the studied area.
Distribution: an endemic species from southern Chile (Malta 1927), distributed between Valdivia Province and Cape Horn (Müller 2009a).
Exsiccata: Fiordo Mitchell (JL 26578).
Notes: new to Capitán Prat.

Ulota larrainii Garilleti, Mazimpaka & F.Lara
Epiphytic on *Nothofagus dombeyi* in Magellanic moorland; alt. 90 m a.s.l.; extremely rare in the studied area.
Distribution: a recently described taxon endemic to Chile, known only from the type specimen here reported (Garilleti et al. 2015).
Exsiccata: Caleta Tortel (JL 26884A + *Ulota billbuckii*).
Notes: recently described by Garilleti et al. (2015).

Ulota luteola (Hook.f. & Wilson) Wijk & Marg.
Epiphytic on *Berberis illicifolia* and on *Nothofagus antarctica* in *Pilgerodendron uviferum* swampy open forests; alt. 20-30 m a.s.l.; common in the studied area.
Distribution: an endemic species from southern Chile (Malta 1927, as *Ulota fulvella* Mitt.), distributed between Osorno Province and Cape Horn (Müller 2009a).
Exsiccata: along Route 7 near Puerto Vagabundo (JL 26461A + *Ulota* sp., JL 26462, JL 26467A + *Ulota pusilla* + *Ulota pycnophylla*), Río Pascua (AJ 107C), Lago Leal (JL 27820A1 + *Porella* sp. +

Ulota rufula + *U. fuegiana*), pasarela Lago Vargas (JL 26834).
Notes: new to Capitán Prat.

Ulota macrocalycina Mitt.
Epiphytic on *Nothofagus pumilio* and on *N. dombeyi* inside the forests; alt. 400 m a.s.l.; not rare in the studied area.
Distribution: an endemic species from southern Chile (Malta 1927), distributed between Osorno Province and Cape Horn (Müller 2009a).
Exsiccata: Route 7 south of Cochrane km 26 (JL 26401A + *Ulota cf. magellanica*), pasarela Río Ñadis (JL 26796B + *Ulota magellanica*).
Notes: new to Capitán Prat and to Aisén Region.

Ulota macrodontia Dixon & Malta
Epiphytic on twigs along river inside the forest; alt. 50 m a.s.l.; rare in the studied area.
Distribution: an endemic species from southern Chile (Malta 1927), distributed between Ñuble and Aisén provinces (Müller 2009a), so this record extends the southern distribution limit of the taxon.
Exsiccata: road S of Cochrane at Tortel/Yungay fork (JL 26479).
Notes: new to Capitán Prat.

Ulota magellanica (Mont.) A.Jaeger
Epiphytic exclusively on *Nothofagus dombeyi*; alt. 5-210 m a.s.l.; common in the studied area.
Distribution: a southern South American endemic species (Malta 1927), reported for Chile between Malleco Province and Cape Horn (Müller 2009a).
Exsiccata: Río Carrera (JL 26594A + *Ulota pusilla*), pasarela Río Ñadis (JL 26796A + *Ulota macrocalycina*), Caleta Tortel (JL 26927A + *Ulota streptodon* + *U. fuegiana* + *U. billbuckii* + *U.pusilla*).
Notes: new to Capitán Prat.

Ulota phyllantha Brid.
Epiphytic on shrubs in a *Pilgerodendron uviferum* swampy open forest; alt. 30 m a.s.l.; rare in the studied area.
Distribution: a bipolar species growing in both coastlines of North America, Iceland, western and northern Europe, Australia, South Africa and Macquarie Is. (Seppelt 1978; Crum and Anderson 1981; Smith 2004), previously known only from Magallanes and the Cape Horn Archipelago (Müller 2009a), so this new record extends its northern distribution limit in Chile.
Exsiccata: along Route 7 near Puerto Vagabundo (JL 26460B + *Ulota pygmaeothecia*).
Notes: new to Capitán Prat and to Aisén Region.

Ulota pusilla Malta
Epiphytic on shrubs in swamp forests, on *Nothofagus dombeyi* in pure *N. dombeyi* forests, or in mixed lowland forest, seldom in stony soil; alt. 5-130 m a.s.l.; common in the studied area.
Distribution: an endemic species from southern Chile (Malta 1927), distributed between Malleco and Magallanes provinces (Müller 2009a).
Exsiccata: along Route 7 near Puerto Vagabundo (JL 26467C + *Ulota luteola* + *U. pycnophylla*), Rio Carrera (JL 26594B + *Ulota magellanica*), Río Bravo (JL 27030A + *Chorisodontium dicranellatum* + *Matteria papillosula* + *Weymouthia mollis*), Lago Quetru (JL 27123B + *Ulota billbucki* + *U. fuegiana*), Caleta Tortel (JL 26927E + *Ulota streptodon* + *U. fuegiana* + *U. magellanica* + *U. billbuckii*).
Notes: new to Capitán Prat and to Aisén Region.

Ulota pycnophylla Dusén ex Malta
Epiphytic on shrubs in *Pilgerodendron uviferum* swampy forests or in *Nothofagus dombeyi* in pure *N. dombeyi* forests; alt. 30-210 m a.s.l.; extremely rare in the studied area.
Distribution: an endemic species from southern Chile (Malta 1927), ignored in the two recent checklists of Chilean mosses (He 1998; Müller 2009a), and so far known only from the type from Angol, Cautín Province, representing this new record a great extension of its distribution range to the south; rare in the studied area.
Exsiccata: along Route 7 near Puerto Vagabundo (JL 26467B + *Ulota luteola* + *U. pusilla*).
Notes: new to Capitán Prat and to Aisén Region. This name might be a synonym of *Ulota macrocalycina*, but there are some morphological differences that would require a thorough study before neglecting this name (R. Garilleti, pers. comm.).

Ulota pygmaeothecia (Müll.Hal.) Kindb.
On dead twigs, epiphytic on *Nothofagus pumilio* and *N. dombeyi*, or on shrubs in burned *Pilgerodendron* forest; alt. 50-360 m a.s.l.; common in the studied area.
Distribution: this taxon grows in New Zealand and southern South America (Seppelt 1978), and it has been reported for Chile between Aisén Province and Cape Horn (Müller 2009b).
Exsiccata: Lago Quetru (JL 27793B + *Chorisodontium dicranelltum* + *Matteria papillosula* + *Ulota rufula*), Lago Chacabuco (JL 27706), along Route 7 near Puerto Vagabundo (JL 26460A + *Ulota phyllantha*), Caleta Tortel (JL 26883).
Notes: new to Capitán Prat.

Ulota rufula (Mitt.) A.Jaeger
Epiphytic on *Azara* shrubs, on *Colletia* sp., on *Nothofagus dombeyi* twigs, in mixed forest understorey; alt. 5-90 m a.s.l.; common in the studied area.
Distribution: this species is distributed in New Zealand and southern South America (Seppelt 1978), and it has been reported for Chile between Ñuble and Aisén provinces, also in Juan Fernández (Müller 2009b), so these records represent a southern extension in its distribution range.
Exsiccata: Lago Quetru (JL 27799c + *Matteria gracillima* + *Cryphaea consimilis*, JL 27787C + *Daltonia gracilis* + *Hypnum cupressiforme* var. *filiforme* + *Eucamptodon perichaetialis* + *Weymouthia cochlearifolia*, JL 27793A + *Matteria papillosula* + *Chorisodontium dicranelltum* + *Ulota pygmaeothecia*), Route 7 south of Cochrane km 90 (JL 27937B + *Orthotrichum pariatum*), Río Bravo (JL 26965B1 + *Matteria papillosula*), Lago Vargas (JL 26809), Caleta Tortel (JL 26928), Lago Leal (JL 27820A2 + *Porella* sp. + *Ulota fuegiana* + *U. luteola*).
Notes: new to Capitán Prat.

Ulota streptodon Garilleti, Mazimpaka & F.Lara
Epiphytic on *Nothofagus dombeyi* and on *Podocarpus* in lowland very humid mixed rainforest; alt. 5-30 m a.s.l.; rare in the studied area.
Distribution: a recently described taxon endemic to Chile, known in the country from the Cape Horn Archipelago and from these collections from Capitán Prat Province (Garilleti et al. 2012).
Exsiccata: Caleta Tortel (JL 26927c + *Ulota billbuckii* + *U. fuegiana* + *U. magellanica* + *U.pusilla*), Fiordo Mitchell (JL 26574).
Notes: recently described by Garilleti et al. (2012).

Zygodon hookeri Hampe var. ***hookeri***
On base of *Berberis*, on dry soil, on rocks, on dead stumps, or epiphytic in lowland mixed forests; alt. 20-260 m a.s.l.; common in the studied area.
Distribution: an austral taxon distributed in New Zealand, Australia and southern South America

(Calabrese 2006), and it has been reported in Chile between Última Esperanza and Tierra del Fuego provinces (Müller 2009a), so these records represent an extension in the northern distribution limit of the species.
Exsiccata: Lago Quetru (JL 27868), Tamango National Reserve (JL 26648B + *Pseudocrossidium* sp.), Playa Vidal at Lago Cochrane (JL 26667A + *Syntrichia fragilis*), road S of Cochrane past Tortel/Yungay fork (JL 26846 p.p. + *Zygodon pentastichus*), Río Bravo (JL 27027).
Notes: new to Capitán Prat and to Aisén Region.

Zygodon hookeri var. ***leptobolax*** (Müll.Hal.) Calabrese
On rocks inside the forest, or epiphytic on *Nothofagus dombeyi* and *Pilgerodendron uviferum*; alt. 20-70 m a.s.l.; common in the studied area.
Distribution: this variety is known from South Africa and southern South America (Calabrese 2006), reported in Chile between Cauquenes and Magallanes provinces (Müller 2009a).
Exsiccata: Route 7 south of Cochrane km 90 (JL 27973), Río Ñadis (JL 26797), pasarela Lago Vargas (JL 26817, JL 26835).
Notes: new to Capitán Prat.

Zygodon magellanicus Dusén ex Malta
On rocks next to streams and on fallen logs inside the forest; alt. 50-340 m a.s.l.; rare in the studied area.
Distribution: a southern South American endemic taxon (Calabrese 2006), reported in Chile between Cauquenes and Tierra del Fuego provinces (Müller 2009a).
Exsiccata: balsa Baker (JL 26685B + *Amphidium tortuosum*), Lago Chacabuco (JL 27709).
Notes: new to Capitán Prat.

Zygodon papillatus Mont.
Epiphytic inside lowland mixed rainforest; alt. 70 m a.s.l.; extremely rare in the studied area.
Distribution: an endemic from the southern Andes, known only from Neuquén Province in Argentina (Calabrese 2006), and between Talca and Magallanes provinces in Chile (Müller 2009a).
Exsiccata: Lago Quetru (JL 27100).
Notes: new to Capitán Prat.

Zygodon pentastichus (Mont.) Müll.Hal.
≡*Pentastichella pentasticha* (Mont.) Müll.Hal. ex Thér.
Epiphytic on *Berberis*, on *Nothofagus*, on Myrtaceae, on dead stumps, on twigs at the forest edge, and on rocks; alt. 5-130 m a.s.l.; very common in the studied area.
Distribution: a southern Andean taxon extending its northern distribution range to Perú, and also recorded for Juan Fernández (Calabrese 2006), in Chile it has been reported between Valparaíso and Magallanes provinces (Müller 2009a).
Exsiccata: road S of Cochrane past Tortel/Yungay fork (JL 26846 p.p. + *Zygodon hookeri* var. *hookeri*), Lago Quetru (JL 27788D + *Eucamptodon perichaetialis* + *Hypnum cupressiforme* var. *filiforme* + *Weymouthia mollis* + *Zygodon reinwardtii*, JL 27870), Río Bravo (JL 26995, JL 27006, JL 27023), road S of Cochrane at Tortel/Yungay fork (JL 26488), Puerto Yungay (JL 26527E).
Notes: new to Capitán Prat.

Zygodon pichinchensis (Taylor) Mitt.
On bases of trees and on rocks in both deciduous *Nothofagus pumilio* and in evergreen *N. dombeyi* forests; alt. 80-340 m a.s.l.; rare in the studied area.
Distribution: an Andean species widely distributed in the tropical Andes between Bolivia and

Venezuela, extending its distribution range into central America and Mexico, with disjunct populations in the southern Andes (Calabrese 2006), reported in Chile between Coihaique and Última Esperanza provinces (Müller 2009a).
Exsiccata: Laguna Esmeralda (JL 27664A), Lago Chacabuco (JL 27725), Río Baker, El Saltón (RR & ET 25).
Notes: new to Capitán Prat.

Zygodon reinwardtii (Hornsch.) A.Braun
On twigs at the forest edge, on fallen logs inside the forest, and epiphytic on *Nothofagus dombeyi*; alt. 50-100 m a.s.l.; common in the studied area.
Distribution: a pantropical species extending into temperate regions of both hemispheres, known from Mexico, Central America, the Andes of South America from Venezuela to Tierra del Fuego, Africa, Madagascar, India, Sri Lanka, SW Asia, China, Alaska, Philippines, Java and New Guinea (Calabrese 2006), and reported in Chile from Osorno Province to Cape Horn (Müller 2009a).
Exsiccata: Lago Quetru (JL 27788C + *Eucamptodon perichaetialis* + *Hypnum cupressiforme* var. *filiforme* + *Weymouthia mollis* + *Zygodon pentastichum*), Lago Leal (JL 27133D + *Ceratodon purpureus* + *Chorisodontium aciphyllum* + *Campylopus pyriformis*), Río Carrera (JL 26601).
Notes: previously reported for the province by Skottsberg (1916, as *Zygodon hyadesii* Besch.) from NW Lago O'Higgins (CS 436).

Order Orthodontiales N.E.Bell, A.E.Newton & D.Quandt
Family Orthodontiaceae Goffinet

Leptotheca gaudichaudii Schwägr.
On soil in different kinds of forests and shrublands, on base of trees, or on fallen trees inside the forest; alt. 5-400 m a.s.l.; one of the most common mosses in the studied area.
Distribution: an austral species growing in southern South America, South Georgia, South Africa, southern Australia, Tasmania, and New Zealand (Churchill and Buck 1982), reported in Chile between Concepción Province and Cape Horn, and in the Juan Fernández Islands (Müller 2009a).
Exsiccata: road S of Cochrane at Tortel/Yungay fork (JL 26490, JL 26495), Laguna Esmeralda (JL 27669), Lago Chacabuco (JL 27729A), pasarela Ñadis (JL 27737, JL 27755, JL 26791), Lago Quetru (JL 27829, JL 27110), Route 7 south of Cochrane km 90 (JL 27950, JL 27952), Río Carrera (JL 26442B + *Pohlia nutans*, JL 26444b + *Pohlia nutans*), Fiordo Mitchell (JL 26540, JL 26557), balsa Baker (JL 26738), pasarela Lago Vargas (JL 26828), Río Bravo (JL 26968, JL 27040A), Lago Leal (JL 27141).
Notes: previously reported for the province by Skottsberg (1916) from NW Lago O'Higgins (CS 627).

Orthodontium lineare Schwägr.
On rotten logs inside the forest; alt. 50 m a.s.l.; rare in the studied area.
Distribution: an austral taxon distributed in southern South America, the Falkland Islands, South Africa, east Africa, New Zealand, southern Australia and Tasmania, introduced and rapidly expanding in Europe (Smith 2004; Hassel and Söderström 2005; Essl et al. 2014), reported for Chile between Llanquihue Province and Cape Horn (Müller 2009a).
Exsiccata: Route 7 south of Cochrane km 90 (JL 27943).
Notes: new to Capitán Prat and to Aisén Region.

Order Aulacomniales N.E.Bell, A.E.Newton & D.Quandt
Family Aulacomniaceae Schimp.

Hymenodontopsis mnioides (Hook.) N.E.Bell, A.E.Newton & D.Quandt (Figure 4H)

≡*Pyrrhobryum mnioides* (Hook.) Manuel
On the forest floor and seldom on rocks and fallen logs inside the forest, in a wide range of evergreen different forests; alt. 20-350 m a.s.l.; very common in the studied area.
Distribution: an austral moss native to new Zealand, Australia, Tasmania and southern South America (Sainsbury 1955), reported in Chile between Aconcagua Province and Cape Horn, as well as in Juan Fernandez Islands (Müller 2009a).
Exsiccata: Lago Chacabuco (JL 27713A), Lago Quetru (JL 27792A), road between Route 7 to Tortel (JL 27909), Route 7 south of Cochrane km 90 (JL 27926), pasarela Lago Vargas (JL 26830), Río Bravo (JL 26990B, JL 26999), Villa O'Higgins (JL 27096), road S of Cochrane at Tortel/Yungay fork (JL 26497), Río Bravo, aeródromo (AJ 11A), Lago Vargas (ET 87, ET 88).
Notes: new to Capitán Prat.

Order Hypnodendrales N.E.Bell, A.E.Newton & D.Quandt
Family Hypnodendraceae Broth.

Hypnodendron microstictum Mitt. ex A.Jaeger & Sauerb.
In very protected, dark places inside thick old-growth forests; alt. 70 m a.s.l.; extremely rare in the studied area.
Distribution: an endemic species of the southern Andes, also growing in the Juan Fernández Islands (Touw 1971), reported in Chile between Cordillera and Aisén provinces (Müller 2009a), so this new record represents an extension in the southern distribution limit of the taxon.
Exsiccata: Lago Quetru (JL 27109).
Notes: new to Capitán Prat.

Order Ptychomniales W.R.Buck, C.J.Cox, A.J.Shaw & Goffinet
Family Ptychomniaceae M.Fleisch.

Glyphothecium sciuroides (Hook.) Hampe
Epiphytic in very wet lowland rainforests, on dead bark of different trees and on *Nothofagus dombeyi*; alt. 50-130 m a.s.l.; not rare in the studied area.
Distribution: an austral taxon growing in Australia, New Zealand, extending north into the tropics to the Philippines and Sri Lanka, and in southern Chile (Hattaway 1984), where it has been reported between Valdivia and Aisén provinces (Müller 2009a), being these new records an extension in the southern distribution limit of the species.
Exsiccata: Lago Quetru (JL 27780), road between Route 7 and Tortel (JL 27898), Río Bravo (JL 27042).
Notes: new to Capitán Prat.

Ombronesus stuvensis N.E.Bell, N.Pedersen & A.E.Newton
Epiphytic on twigs of *Nothofagus dombeyi* or shrubs like *Azara* sp. inside very wet lowland rainforests; alt. 30-50 m a.s.l.; not rare in the most wet places of the studied area.
Distribution: a recently described taxon formerly known only from the type material collected in Juan Stuven Island, Katalalixar National Reserve, just west of Caleta Tortel (Bell et al. 2007), so far believed to be the only genus endemic to Capitán Prat Province.
Exsiccata: Lago Quetru (JL 27798A, JL 27800, JL 27842), road between Route 7 and Tortel (JL 27897A), Juan Stuven Island (NB 1247, type).
Notes: recently described by Bell et al. (2007).

Ptychomniella ptychocarpon (Schwägr.) W.R.Buck, C.J.Cox, A.J.Shaw & Goffinet

≡*Ptychomnion ptychocarpon* (Schwägr.) Mitt.
Epiphytic on *Podocarpus nubigena*, *Nothofagus dombeyi*, and *N. nitida* in lowland wet rainforests, seldom on rocks or on soil in the forest floor; alt. 30-90 m a.s.l.; common in the studied area.
Distribution: an endemic taxon of southern Chile and Juan Fernández (Hattaway 1984), reported for Chile between Valparaíso and Magallanes provinces (Müller 2009a).
Exsiccata: Lago Quetru (JL 27796A + *Ancistrodes genuflexa*, JL 27835, JL 27770A), road between Route 7 and Tortel (JL 27907B, JL 27913), road S of Cochrane at Tortel/Yungay fork (JL 26492), Fiordo Mitchell (JL 26576, AJ 95), Río Bravo (JL 26975A), Lago Leal (JL 27818A2 + *Ptychomnion cygnisetum*).
Notes: previously reported for the province by Skottsberg (1916) from Caleta Hale (CS 952).

Ptychomnion cygnisetum (Müll.Hal.) Kindb.
On the forest floor, seldom on base of *Nothofagus dombeyi*, on tree stumps, or on peat; alt. 20-90 m a.s.l.; very common in the studied area.
Distribution: an endemic taxon of the southern Andes rainforests (Hattaway 1984), reported in Chile between Coquimbo Province and Cape Horn, as well as in Juan Fernández Islands (Müller 2009a).
Exsiccata: Río Bravo (JL 26988, JL 26966, JL 27000, AJ 11B, AM 1046A), Route 7 south of Cochrane km 90 (JL 27949), pasarela Lago Vargas (JL 26825), Caleta Tortel (JL 26919, JL 26920), Lago Quetru (JL 27845), road S of Cochrane at Tortel/Yungay fork (JL 26500), road between Route 7 and Tortel (JL 27905 p.p. + *Arbusculohypopterygium arbuscula*), Lago Leal (JL 27818A1 + *Ptychomniella ptychocarpon*), Fiordo Mitchell (AJ 93B).
Notes: new to Capitán Prat.

Order Hookeriales M.Fleisch.
Family Hypopterygiaceae Mitt.

Arbusculohypopterygium arbuscula (Brid.) M.Stech, T.Pfeiff. & W.Frey
≡*Hypopterygium arbuscula* Brid.
≡*Dendrohypopterygium arbuscula* (Brid.) Kruijer
Exclusively on the forest floor in very wet lowland rainforests of *Nothofagus dombeyi, Drimys winteri, Podocarpus nubigena,* and *Weinmannia trichosperma*; alt. 30-50 m a.s.l.; common in the studied area.
Distribution: a southern South American endemic with a couple of populations known from the yungas of Perú and Bolivia, also in the Juan Fernandez Islands (Kruijer 2002), reported in Chile between Aconcagua and Tierra del Fuego provinces (Müller 2009a).
Exsiccata: Lago Quetru (JL 27802, JL 27849), Fiordo Mitchell (JL 26566, AJ 93C), road between Route 7 and Tortel (JL 27905b + *Ptychomnion cygnisetum*), Río Bravo (AJ 11C).
Notes: previously reported for the province by Skottsberg (1916) from Caleta Hale (CS s.n.).

Hypopterygium didictyon Müll.Hal.
On the forest floor, on base of tree trunks, or on stumps or epiphytic on twigs inside *Drimys-Podocarpus-Amomyrtus* forests; alt. 20-50 m a.s.l.; not common in the studied area.
Distribution: an austral taxon distributed in south Australia, Tasmania, New Zealand, and southern South America (Kruijer 2002), reported for Chile between Valparaíso and Cape Horn (Müller 2009a).
Exsiccata: Fiordo Mitchell (JL 26563, JL 26573), Río Bravo (JL 27152, AJ 12), road between Route 7 and Tortel (JL 27899 p.p. + *Lepyrodon* sp. + *Zygodon* sp. + *Ancistroes genuflexa* + *Plagiochila* sp.).
Notes: new to Capitán Prat.

Lopidium concinnum (Hook.) Wilson
On rotten log in *Drimys-Podocarpus-Amomyrtus* forest; alt. 50 m a.s.l.; extremely rare in the studied

area.
Distribution: an austral taxon distributed in east and south Australia, Tasmania, New Zealand, Bolivian yungas, SE Brazil and southern South America including the Juan Fernández Islands (Kruijer 2002), formerly known in Chile between Valparaíso and Aisén provinces (Müller 2009a), so this record extends its distribution range to the south.
Exsiccata: road between Route 7 and Tortel (JL 27903B1).
Notes: new to Capitán Prat.

Family Saulomataceae W.R.Buck, C.J.Cox, A.J.Shaw & Goffinet

Ancistrodes genuflexa (Müll.Hal.) Crosby
Epiphytic on twigs of *Podocarpus nubigena*, and on dead twigs in *Drimys-Podocarpus-Amomyrtus* forest; alt. 15-130 m a.s.l.; common in the studied area.
Distribution: an endemic species of the Valdivian rainforests (Calabrese and Matteri 1999), previously reported for Chile between Cauquenes and Aisén provinces (Müller 2009a), so these new records extend the species distribution range to the south.
Exsiccata: Lago Quetru (JL 27796B + *Ptychomniella ptychocarpon*, JL 27836), road between Route 7 and Tortel (JL 27901, JL 26849, JL 27899d + *Lepyrodon* sp. + *Zygodon* sp. + *Hypopterygium didictyon* + *Plagiochila* sp.), Río Bravo (JL 27041).
Notes: new to Capitán Prat.

Sauloma tenella (Hook.f. & Wilson) Mitt.
On the forest floor or on tree stumps inside very wet *Drimys-Weinmannia-Podocarpus* rainforests; alt. 30 m a.s.l.; rare in the studied area.
Distribution: an austral taxon native to New Zealand, Tasmania and southern South America (Matteri 1972), reported in Chile between Llanquihue and Cape Horn (Müller 2009a).
Exsiccata: Fiordo Mitchell (JL 26583b + *Achrophyllum magellanicum*, JL 26584).
Notes: new to Capitán Prat.

Family Daltoniaceae Schimp.

Achrophyllum anomalum (Schwägr.) H.Rob. var. ***anomalum***
On the forest floor, in *Nothofagus dombeyi-Drimys winteri-Podocarpus nubigena* rainforest; alt. 90 m a.s.l.; extremely rare in the studied area.
Distribution: an endemic taxon from southern South America and the Juan Fernández Islands (Robinson 1975), reported in Chile between Malleco Province and Cape Horn (Müller 2009a).
Exsiccata: Río Bravo (JL 26989).
Notes: new to Capitán Prat.

Achrophyllum anomalum var. ***pallidum*** (Cardot & Broth.) S.He
Distribution: this variety is so far known only from Caleta Hale in Canal Messier, Capitán Prat Province (Skottsberg 1916; Cardot and Brotherus 1923).
Notes: previously reported for the province by Cardot and Brotherus (1923) and Skottsberg (1916) as *Pterygophyllum anomalum* (Schwägr.) Mitt. var. *pallidum* Cardot & Broth. from Caleta Hale (CS 796).

Achrophyllum haesselianum (Matteri) Matteri
On the forest floor, in wet *Nothofagus dombeyi-Drimys winteri-Podocarpus nubigena* forest; alt. 30-130 m a.s.l.; rare in the studied area.
Distribution: an endemic taxon from southern South America (Matteri 1972), reported in Chile between

Aisén Province and Cape Horn (Müller 2009a).
Exsiccata: Lago Quetru (JL 27854, JL 27850 p.p. + *Achrophyllum magellanicum*), Río Bravo (JL 26990A, JL 27038).
Notes: new to Capitán Prat.

Achrophyllum magellanicum (Besch.) Matteri (Figure 3G)
On the forest floor and on creek banks in very wet lowland forests, seldom on rotten wood; alt. 30-100 m a.s.l.; common in the studied area.
Distribution: an endemic taxon from southern South America extending into the Falkland Islands (Matteri 1972), reported for Chile between Talca Province and Cape Horn (Müller 2009a).
Exsiccata: Lago Quetru (JL 27850b + *Acrophyllum haesselianum*, JL 27844, JL 27112), Fiordo Mitchell (JL 26562, JL 26567, JL 26583 p.p. + *Sauloma tenella*), road between Route 7 and Tortel (JL 27892, JL 27902, JL 27903A, JL 27904), balsa Baker (JL 26708), Río Bravo (JL 26981), Lago Leal (JL 27136), Río Ñadis (AJ 91).
Notes: new to Capitán Prat.

Daltonia gracilis Mitt.
Epiphytic on twigs of trees and shrubs like *Fuchsia magellanica* inside very wet *Nothofagus dombeyi-Podocarpus nubigena-Drimys winteri* rainforests; alt. 50-130 m a.s.l.; rare in the studied area.
Distribution: a South American taxon found throughout the Andes and extending north to Costa Rica (Matteri 1975), reported in Chile between Arauco and Magallanes provinces, and also in Juan Fernández (Müller 2009a).
Exsiccata: Lago Quetru (JL 27787D + *Eucamptodon perichaetialis* + *Hypnum cupressiforme* var. *filiforme* + *Ulota rufula* + *Weymouthia cochlearifolia*, JL 27831), Río Bravo (JL 27043), Fiordo Mitchell (JL 26585).
Notes: new to Capitán Prat.

Daltonia trachyodonta Mitt.
Epiphytic on twigs inside very wet *Nothofagus dombeyi-Podocarpus nubigena-Drimys winteri* rainforests; alt. 50 m a.s.l.; extremely rare in the studied area.
Distribution: an Andean species found in the tropical Andes of Ecuador and Bolivia and disjunct in southern South America (Matteri 1975), previously known in Chile between Bío-Bío and Aisén provinces (Müller 2009a), so this record represents a southern extension of its distribution range.
Exsiccata: Río Bravo (JL 27018).
Notes: new to Capitán Prat.

Distichophyllum dicksonii (Hook. & Grev.) Mitt.
In vary shady and humid places in the forest floor or epiphytic on *Dendroligotrichum dendroides*, in old-growth *Nothofagus dombeyi-Drimys winteri-Podocarpus nubigena-Weinmannia trichosperma* forests; alt. 30-70 m a.s.l.; rare in the studied area.
Distribution: an endemic species from southern South America extending into the Falkland Islands (Matteri 1975), reported for Chile between Valdivia Province and Cape Horn (Müller 2009a).
Exsiccata:, Lago Quetru (JL 27104A, JL 27846c + *Rhaphidorrhynchium dendroligotrichum* + *Dendroligotrichum dendroides*).
Notes: new to Capitán Prat.

Order Hypnales (M.Fleisch.) W.R.Buck & Vitt
Family Amblystegiaceae G.Roth

Campylium stellatum (Hedw.) C.E.O.Jensen
On wet soil in river bank running along the steppe, intermingled with *Dicranella hookeri*; alt. 430 m a.s.l.; extremely rare in the studied area.
Distribution: a bipolar species with some high-elevation stations in the tropics, known from temperate and arctic North America, Europe and Asia, N and E Africa, Australia, New Zealand, the Neotropics (Hedenäs 2003), and southern South America (Matteri 2003), previously reported for Chile from a single record in Última Esperanza Province (Müller 2009a), so this record represents a northern extension of its distribution range in Chile.
Exsiccata: Estancia Chacabuco (JL 26949B + *Dicranella hookeri*).
Notes: new to Capitán Prat and to Aisén Region.

Drepanocladus aduncus (Hedw.) Warnst.
On fallen logs on creek bank inside the forest; alt. 50 m a.s.l.; extremely rare in the studied area.
Distribution: an almost cosmopolitan species (Hedenäs 2003), reported for Chile between Santiago Province and Tierra del Fuego (Müller 2009a).
Exsiccata: road S of Cochrane at Tortel/Yungay fork (JL 26491).
Notes: new to Capitán Prat and to Aisén Region.

Drepanocladus longifolius (Mitt.) Broth. ex Paris
Submerged in creek inside *Nothofagus pumilio* forest; alt. 400 m a.s.l.; extremely rare in the studied area.
Distribution: an austral species growing in the tropical Andes, southern South America and the Falkland Islands, South Georgia, Kerguelen, Australia (Hedenäs 2003), and Antarctica (Ochyra et al. 2008), reported for Chile between Cordillera Province and Cape Horn (Müller 2009a).
Exsiccata: Route 7 south of Cochrane km 26 (JL 26403).
Notes: new to Capitán Prat.

Drepanocladus polygamus (Schimp.) Hedenäs
On wet soil in river bank running along the steppe; alt. 430 m a.s.l.; extremely rare in the studied area.
Distribution: a bipolar taxon with some occurrences at high elevation in the tropics (Hedenäs 2003; Ochyra and Matteri 2001; Ochyra et al. 2008), reported for Chile between Copiapó Province and Tierra del Fuego (Müller 2009a).
Exsiccata: Estancia Chacabuco (JL 26943).
Notes: new to Capitán Prat.

Cratoneuropsis relaxa (Hook.f. & Wilson) M.Fleisch. ex Broth. subsp. ***minor*** (Hook.f. & Wilson) Ochyra
=*Orthotheciella varia* (Hedw.) Ochyra
=*Hygroamblystegium chilense* (Lorentz) Reimers
=*Hygroamblystegium filum* (Müll.Hal.) Reimers
=*Pseudoleskea fuegiana* (Besch.) Broth.
=*Cratoneuropsis chilensis* (Lorentz) Ochyra
On wet soil in river banks, on the forest floor, on fallen logs, on rocks in streams, and on bases of shrubs and trees inside the forest; alt. 50-340 m a.s.l.; very common in the studied area.
Distribution: a circumsubantarctic species known from southern South America, the Falkland Is., most of the subantarctic islands, South Africa and New Zealand (Ochyra et al. 2008), reported in Chile between Antofagasta Province and Cape Horn (Müller 2009a).
Exsiccata: balsa Baker (JL 26676, JL 26698, JL 26706b + *Brachytheciastrum paradoxum*, JL 26689b + *Hypnum cupressiforme* var. *cupressiforme*), Lago Chacabuco (JL 27712, JL 27708b, JL 27731A +

Tortella knightii, JL 27693E + *Acrocladium auriculatum* + *Syntrichia anderssonii* + *Polytrichum juniperinum*), Caleta Tortel (JL 27886), Villa O'Higgins (JL 27083), pasarela El Salto (JL 27642, JL 27651, JL 27659), Laguna Esmeralda (JL 27663A, JL 27664B), Tamango National Reserve (JL 26629, JL 26655), Playa Vidal at Lago Cochrane (JL 26663), Río Ñadis (JL 26789), Estancia Chacabuco (JL 26946), Lago Chico (ET 148b + *Lepyrodon lagurus* + *L. patagonicus* + *Bartramia mossmaniana* + *Hypnum skottsbergii* + *Sanionia uncinata*).
Notes: first reported for the province by Skottsberg (1916) as *Pseudoleskea fuegiana* (Besch.) Broth. from NW Lago O'Higgins (CS 804).

Sanionia uncinata (Hedw.) Loeske
On the forst floor in *Nothofagus dombeyi* and *N. pumilio* forests, seldom on peatlands and in open swampy shrubland; alt. 20-400 m a.s.l.; very common in the studied area.
Distribution: a wide-spread bipolar taxon with a few stations at high elevation in the tropics (Ochyra et al. 2008), reported in Chile between Ñuble Province and Cape Horn (Müller 2009a).
Exsiccata: Río Bravo (JL 27002, JL 27035), road between Route 7 and Tortel (JL 26429B), Río Carrera (JL 26453, JL 26589, JL 26605), Puerto Yungay (JL 26509, JL 26521), Tamango National Reserve (JL 26616), balsa Baker (JL 26739), pasarela Lago Vargas (JL 26812), Lago Chico (ET 148c + *Lepyrodon lagurus* + *L. patagonicus* + *Cratoneuropsis relaxa* subsp. *minor* + *Bartramia mossmaniana* + *Hypnum skottsbergii*).
Notes: new to Capitán Prat.

Scorpidium revolvens (Sw.) Rubers
≡*Limprichtia revolvens* (Sw.) Loeske
Submerged in slow flowing rivulet, to 40 cm deep, in the middle of dry steppe; alt. 430 m a.s.l.; extremely rare in the studied area.
Distribution: a bipolar species wide-spread in the Northern Hemisphere and occurring in southern South America, S Australia and New Zealand, with a few known populations in New Guinea (Ochyra and Matteri 2001), reported in Chile only from Magallanes region (Müller 2009a), so these reports represent an extension to the north of the distribution limit of the species in Chile.
Exsiccata: Estancia Chacabuco (JL 26944, JL 26950).
Notes: new to Capitán Prat and to Aisén Region.

Vittia pachyloma (Mont.) Ochyra
On rocks and stones in fast flowing streams both inside *Nothofagus pumilio* forests or in open areas; alt. 130-400 m a.s.l.; rare in the studied area.
Distribution: an austral taxon ocurring throughout South America, the Falkland Islands, South Georgia and South Africa (Ochyra and Matteri 2001), reported in Chile between Los Andes Province and Cape Horn, and in the Juan Fernández Islands (Müller 2009a).
Exsiccata: Río Bravo (JL 27026), balsa Baker (JL 26732, JL 26744).
Notes: new to Capitán Prat.

Family Calliergonaceae Vanderpoorten, Hedenäs, C.J.Cox & A.J.Shaw

Straminergon stramineum (Dicks. ex Brid.) Hedenäs
≡*Calliergon stramineum* (Dicks. ex Brid.) Kindb.
Distribution: a wide-spread Northern Hemisphere taxon, also known from the Neotropics (Hedenäs 2003), reported in Chile only from Capitán Prat Province (Donat 1936a, 1936b).
Notes: the unique report for this species in southern South America is that of Donat (1936a, 1936b as *Calliergon stramineum* Dicks.) from glacier moraines at Lago O'Higgins (AD s.n.), incorrectly

attributed to Aisén Province by Müller (2009a). As Hedenäs (2003) already noted, the voucher specimen needs to be checked to confirm the presence of this taxon in Patagonia.

Warnstorfia exannulata (Schimp.) Loeske
On saturated soil in peatlands and fens; alt. 40-50 m a.s.l.; rare in the studied area.
Distribution: a bipolar taxon wide-spread in the Northern Hemisphere, and occurring in Perú, southern South America, the Falkland Is., South Georgia and New Zealand (Ochyra and Matteri 2001), reported in Chile between Santiago Province and Cape Horn (Müller 2009a).
Exsiccata: Route 7 south of Cochrane past Lago Vargas fork (JL 26436, JL 26437B), Río Carrera (JL 26451).
Notes: new to Capitán Prat.

Warnstorfia fluitans (Hedw.) Loeske
On open soil intermingled with *Sphagnum* mats; alt. 380 m a.s.l.; extremely rare in the studied area.
Distribution: a wide-spread taxon known from the Northern Hemisphere, Africa, New Guinea, Australia, New Zealand, and southern South America (Ochyra and Matteri 2001), reported for Chile between San Antonio Province and Cape Horn (Müller 2009a).
Exsiccata: Route 7 between Tortel/Yungay frok and Yungay settlement (JL 26506).
Notes: new to Capitán Prat and to Aisén Region.

Warnstorfia fontinialopsis (Müll.Hal.) Ochyra
On soil at *Nothofagus dombeyi-Drimys winteri-Podocarpus nubigena* forest; alt. 90 m a.s.l.; extremely rare in the studied area.
Distribution: an austral taxon recorded for southern South America, South Georgia, Prince Edward Is., Kerguelen, the maritime Antarctic, and New Zealand (Ochyra and Matteri 2001; Ochyra et al. 2008), reported for Chile between Aisén and Tierra del Fuego provinces (Müller 2009a).
Exsiccata: Río Bravo (JL 26986).
Notes: new to Capitán Prat. Recent molecular studies suggest that this species might be a synonym of the Northern Hemisphere *Warnstorfia pseudostraminea* (Müll.Hal.) Tuom. & T.J.Kop. (Hedenäs 2011).

Family Brachytheciaceae G.Roth

Brachytheciastrum paradoxum (Hook.f. & Wilson) Ignatov & Huttunen
≡*Brachythecium paradoxum* (Hook.f. & Wilson) A.Jaeger
On soil and litter in the forest floor, on decaying wood, on tree roots and bases, and on rocks, in *Nothofagus dombeyi, N. pumilio* or *N. antarctica* forests; alt. 5-340 m a.s.l.; very common in the studied area.
Distribution: an austral species ocurring in southern South America, subantarctic islands (Crozet, Kerguelen, Marion, Prince Edward), South Africa, Tasmania and New Zealand (McFarland 1988), reported for Chile between Cordillera Province and Cape Horn (Müller 2009a).
Exsiccata: Tamango National Reserve (JL 26630), balsa Baker (JL 26726, JL 26706 p.p. + *Cratoneuropsis relaxa* subsp. *minor*), pasarela Río Ñadis (JL 26780, JL 26795, JL 27753A + *Syntrichia anderssonii*), Caleta Tortel (JL 26923 p.p. + *Ceratodon purpureus*), pasarela El Salto (JL 27638), Laguna Esmeralda (JL 27666), Lago Chacabuco (JL 27714, JL 27718b), road between Route 7 and Tortel (JL 26420), Lago Vargas (AJ 201).
Notes: first reported for Capitán Prat by Skotsberg (1916) as *Brachythecium paradoxum* (Hook.f. & Wilson) A. Jaeger, from NE Lago O'Higgins (CS 830).

Brachythecium albicans (Hedw.) Schimp.

In human disturbed places, on wood or sandy soil, seldom on base of *Nothofagus dombeyi* in disturbed forests; alt. 1-50 m a.s.l.; not common in the studied area.
Distribution: a bipolar species, occurring in both coasts of North America, Greenland, Macaronesia, Caucasus, Australia, New Zealand, southern South America and the Falkland Is. (McFarland 1988), reported in Chile between Santiago Province and Cape Horn (Müller 2009a).
Exsiccata: Caleta Tortel (JL 27888A, JL 26921), Puerto Yungay (JL 27763), balsa Baker (JL 26679).
Notes: new to Capitán Prat.

Brachythecium austroglareosum (Müll.Hal.) Paris
On dry soil in disturbed forests and grasslands; alt. 70-260 m a.s.l.; not common in the studied area.
Distribution: a circumsubantarctic species reported for southern South America, Falkland Is., South Georgia, Is. Crozet, Kerguelen, Gough Is. and Auckland Is., Antarctica, and with a few disjunct populations in the tropical Andes (Ochyra et al. 2008), reported for Chile between Chiloé Province and Cape Horn (Müller 2009a).
Exsiccata: Tamango National Reserve (JL 26654), pasarela Río Ñadis (JL 26782), pasarela El Salto (JL 27657).
Notes: new to Capitán Prat.

Brachythecium austrosalebrosum (Müll.Hal.) Paris
On soil inside *Nothofagus dombeyi* forest; alt. 160 m a.s.l.; extremely rare in the studied area.
Distribution: a wide-spread circumsubantarctic species occurring in all subantarctic islands and reaching Antarctica (Ochyra et al. 2008), reported for Chile between Aisén Province and Cape Horn (Müller 2009a).
Exsiccata: Playa Vidal at Lago Cochrane (JL 26670).
Notes: new to Capitán Prat.

Brachythecium subpilosum (Hook.f. & Wilson) A.Jaeger
On litter on the floor of *Nothofagus dombeyi* forest; alt. 100 m a.s.l.; extremely rare in the studied area.
Distribution: an austral taxon occurring in southern South America, Falkland Is., South Georgia, Kerguelen, Tristan da Cunha and Antarctica (Ochyra et al. 2008), reported for Chile between Ñuble Province and Cape Horn (Müller 2009a).
Exsiccata: Río Carrera (JL 26590).
Notes: recently reported for Capitán Prat Province by Cuvertino et al. (2012).

Brachythecium subplicatum (Hampe) A.Jaeger
On wet floor in *Nothofagus dombeyi* forest, humid soil in river banks or along creeks inside the water; alt. 100-430 m a.s.l.; rare in the studied area.
Distribution: an austral taxon occurring in southern South America, Falkland Is., South Georgia, Kerguelen, Crozet Is., and New Zealand (McFarland 1988), reported for Chile between Talca Province and Cape Horn (Müller 2009a).
Exsiccata: Route 7 south of Cochrane km 26 (JL 26402), Río Carrera (JL 26599), Estancia Chacabuco (JL 26948).
Notes: new to Capitán Prat.

Eriodon conostomus Mont.
Epiphytic on *Fuchsia magellanica* in very wet *Nothofagus dombeyi-Drimys winteri-Podocarpus nubigena* old-growth forest; alt. 30 m a.s.l.; extremely rare in the studied area.
Distribution: an endemic moss of the Valdivian rain forests (Larraín 2007), previously reported in Chile between Bío-Bío and Aisén provinces (Müller 2009a), so this new records extend the southern

distribution range of the species.
Exsiccata: Lago Quetru (JL 27833, JL 27830A + *Plagiochila* sp. + *Metzgeria* sp.).
Notes: new to Capitán Prat.

Eurhynchiella acanthophylla (Mont.) M.Fleisch.
On stones on the floor of *Nothofagus dombeyi-Embothrium coccineum-Amomyrtus luma* forest; alt. 50 m a.s.l.; extremely rare in the studied area.
Distribution: an endemic moss of central Chile and Patagonia (Seki 1974), previously reported in Chile between Choapa and Aisén provinces (Müller 2009a), so this new record extends the southern distribution range of the species.
Exsiccata: Route 7 south of Cochrane km 90 (JL 27972B + *Fissidens curvatus*).
Notes: new to Capitán Prat.

Sciuro-hypnum plumosum (Hedw.) Ignatov & Huttunen
=*Brachythecium plumosum* (Hedw.) Schimp.
On litter on the floor of *Nothofagus dombeyi* forest; alt. 60 m a.s.l.; extremely rare in the studied area.
Distribution: a cosmopolitan taxon (McFarland 1988) reported in Chile between Ñuble and Magallanes provinces (Müller 2009a).
Exsiccata: balsa Baker (JL 26717).
Notes: new to Capitán Prat and to Aisén Region.

Family Fabroniaceae Schimp.

Fabronia jamesonii Taylor
On rocks in dry shrublands; alt. 160-190 m a.s.l.; rare in the studied area.
Distribution: a South American taxon occurring in the Andes of Ecuador south to Patagonia, in Uruguay, and on Easter Island (Buck 1983), reported for Chile between Valparaíso and Magallanes provinces (Müller 2009a).
Exsiccata: Tamango National Reserve (JL 26609), Playa Vidal at Lago Cochrane (JL 26664).
Notes: new to Capitán Prat and to Aisén Region.

Family Hypnaceae Schimp.

Hypnum chrysogaster Müll.Hal.
Epiphytic, often pendant, seldom on rocks or on the forest floor, in lowland evergreen humid forests; alt. 30-100 m a.s.l.; common in the studied area.
Distribution: a southern South American-Australasian taxon, known from SE Australia, New Zealand and Chile (Ando 1972a, 1982), where it has been reported between Cauquenes and Aisén provinces, as well as in Juan Fernández (Müller 2009a), so this record extends its distribution limit in Chile to the south.
Exsiccata: Lago Leal (JL 27816A), road between Route 7 and Tortel (JL 27912, JL 27911, JL 26429A), Caleta Tortel (JL 26882), Lago Quetru (JL 27113, JL 27121, JL 27839), Fiordo Mitchell (JL 26572, JL 26586).
Notes: new to Capitán Prat.

Hypnum cupressiforme Hedw. var. ***cupressiforme***
On rocks in creek along *Nothofagus dombeyi* forest; alt. 100 m a.s.l.; extremely rare in the studied area.
Distribution: a cosmopolitan moss (Ando 1972b), reported in Chile between Choapa and Magallanes provinces (Müller 2009a).

Exsiccata: balsa Baker (JL 26689 p.p. + *Cratoneuropsis relaxa* subsp. *minor*).
Notes: new to Capitán Prat.

Hypnum cupressiforme var. ***mossmanianum*** (Müll.Hal.) Ando
On soil in creek banks or on decaying wood inside *Nothofagus dombeyi* forest; alt. 50-100 m a.s.l.; rare in the studied area.
Distribution: an austral taxon, known from SE Australia, Tasmania, New Zealand, South Africa and southern South America (Ando 1982; Ando and Matteri 1982), reported in Chile between Malleco Province and Cape Horn (Müller 2009a).
Exsiccata: road between Route 7 and Tortel (JL 26417), Lago Leal (JL 27806A).
Notes: new to Capitán Prat.

Hypnum cupressiforme var. ***filiforme*** Brid.
Epiphytic on twigs of *Gaultheria insana*, *Mitraria coccinea*, *Nothofagus dombeyi*, or on tree bases inside the forest; alt. 15-70 m a.s.l.; common in the studied area.
Distribution: a bipolar taxon wide-spread in the Northern Hemisphere and occurring in SE Australia, New Zealand and southern South America (Ando 1982), previously known in Chile between Ñuble and Llanquihue provinces (Müller 2009a), so these records represent an extension in the southern distribution range of the taxon in Chile.
Exsiccata: pasarela Ñadis (JL 27745A), Lago Quetru (JL 27120, JL 27786, JL 27788B + *Eucamptodon perichaetialis* + *Weymouthia mollis* + *Zygodon pentastichum* + *Z. reinwardtii*, JL 27787A1 + *Daltonia gracilis* + *Eucamptodon perichaetialis* + *Ulota rufula* + *Weymouthia cochlearifolia*), road between Route 7 and Tortel (JL 26854), balsa Baker (JL 26724 p.p. + *Syntrichia anderssonii*), Route 7 south of Cochrane km 90 (JL 27955).
Notes: new to Capitán Prat and to Aisén Region.

Hypnum skottsbergii Ando
On the forest floor, on fallen logs, on decaying wood, on rocks, on roots and bases of trees inside different kinds of forests; alt. 30-300 m a.s.l.; very common in the studied area.
Distribution: an endemic species of southern South America (Ando and Matteri 1982), reported for Chile between Malleco Province and Cape Horn (Müller 2009a).
Exsiccata: pasarela El Salto (JL 27641, JL 27654), Laguna Esmeralda (JL 27660A), Lago Quetru (JL 27863A, JL 27873), Route 7 south of Cochrane km 10 (JL 26387), balsa Baker (JL 26727), pasarela lago Vargas (JL 26826), Villa O'Higgins (JL 27088), Lago Chico (ET 148d + *Lepyrodon lagurus* + *L. patagonicus* + *Hygroamblystegium chilense* + *Bartramia mossmaniana* + *Sanionia uncinata*).
Notes: new to Capitán Prat.

Mahua enervis W.R.Buck
On rocks inside a *Nothofagus-Embothrium-Amomyrtus* forest; alt. 50 m a.s.l.; extremely rare in the studied area.
Distribution: a Chilean endemic taxon (Buck 1983), previously known in Chile only from the type from Lago Pellaifa, Valdivia Province (Buck 1983, Müller 2009a), so this record represents the second known locality of the taxon, and an extension to its distribution limit to the south.
Exsiccata: Route 7 south of Cochrane km 90 (JL 27965, JL 27970).
Notes: new to Capitán Prat and to Aisén Region.

Family Catagoniaceae W.R.Buck & Ireland

Catagonium nitens (Brid.) Cardot

Epiphytic on *Podocarpus nubigena* or on *Amomyrtus luma*, in lowland evergreen forests; alt. 50-130 m a.s.l.; rare in the studied area.
Distribution: a southern hemispheric taxon distributed in Australia, New Zealand, New Guinea, southern Africa, Réunion, subantarctic islands and southern South America (Lin 1984), reported in Chile from Talca Province to Cape Horn, and in the Juan Fernández Islands (Müller 2009a), and from Neuquén, Río Negro and Tierra del Fuego provinces in Argentina (Matteri 2003).
Exsiccata: road to Tortel (JL 27894A), Río Bravo (JL 27048).
Notes: new to Capitán Prat.

Family Plagiotheciaceae M.Fleisch.

Plagiothecium lucidum (Hook.f. & Wilson) Paris
On base of *Embothrium coccineum* inside a *Nothofagus-Embothrium-Amomyrtus* forest; alt. 50 m a.s.l.; rare in the studied area.
Distribution: a Neotropical taxon, extending its distribution from the Andes of Venezuela to Bolivia, and also in the Dominican Republic and SE Brazil (Buck and Ireland 1989), previously reported in Chile from all four provinces of Magallanes Region (Müller 2009a), so this record extends its distribution limit in Chile to the north.
Exsiccata: Route 7 south of Cochrane km 90 (JL 27956).
Notes: new to Capitán Prat and to Aisén Region.

Family Sematophyllaceae Broth.

Rhaphidorrhynchium amoenum (Hedw.) M.Fleisch.
On rocks in creek inside the forest; alt. 30 m a.s.l.; extremely rare in the studied area.
Distribution: a southern South American-Australasian taxon (Ramsay 2012), known from New Zealand (Sainsbury 1955) and Australia (Streimann and Curnow 1989), reported in Chile between Arauco Province and Cape Horn (Müller 2009a).
Exsiccata: Fiordo Mitchell (JL 26581).
Notes: new to Capitán Prat and to Aisén Region.

Rhaphidorrhynchium callidum (Mont.) Broth. (Figure 3D)
On fallen logs or epiphytic on horizontal branches, seldom on rocks, inside lowland evergreen forests; alt. 20-130 m a.s.l.; common in the studied area.
Distribution: a southern South American endemic taxon (Seki 1974) previously reported in Chile from Limarí to Magallanes provinces (Müller 2009a).
Exsiccata: Lago Leal (JL 27817), Route 7 south of Cochrane km 90 (JL 27944, JL 27954, JL 27929), pasarela Lago Vargas (JL 26831), Río Bravo (JL 26970, JL 27019, JL 27045), Lago Quetru (JL 27119), Fiordo Mitchell (JL 26575), road to Tortel (JL 27893), Route 7 at fork Tortel/Yungay (JL 26494).
Notes: new to Capitán Prat.

Rhaphidorrhynchium dendroligotrichum (Dusén) Broth.
An exclusive epiphyte on *Dendroligotrichum dendroides*, which grows on the forest floor, found only in very humid lowland evergreen forests; alt. 30-90 m a.s.l.; rare in the studied area.
Distribution: a narrow Chilean endemic taxon (Seki 1974), previously known only from Aisén Province (Müller 2009a), so this record extends its southern distribution limit.
Exsiccata: road to Tortel (JL 27906b + *Dendroligotrichum dendroides*, JL 27910b + *Dendroligotrichum dendroides*), Río Bravo (JL 26997b + *Dendroligotrichum dendroides*), Lago Quetru (JL 27846b + *Dendroligotrichum dendroides* + *Distichophyllum dicksonii*).

Notes: new to Capitán Prat.

Sematophyllum scorpiurus (Mont.) Mitt.
On fallen logs, on the forest floor or on rocks in rivulets, in lowland evergreen forests; alt. 5-70 m a.s.l.; rare in the studied area.
Distribution: a Chilean endemic taxon (Seki 1974), previously reported from Arauco to Tierra del Fuego provinces (Müller 2009a).
Exsiccata: Lago Leal (JL 27140, JL 27815), Fiordo Mitchell (JL 26556).
Notes: new to Capitán Prat.

Family Cryphaeaceae Schimp.

Cryphaea consimilis Mont.
Epiphitic on *Campsidium*, *Azara*, *Nothofagus* or *Fuchsia*, in lowland evergreen forests; alt. 20-50 m a.s.l.; rare in the studied area.
Distribution: a Chilean endemic taxon (Rao 2001) previously reported from Talca to Aisén provinces (Müller 2009a), so this record extends its southern distribution limit.
Exsiccata: Río Bravo (JL 27148), Route 7 south of Cochrane km 90 (JL 27938 p.p. + *Calyptopogon mnioides*), Lago Quetru (JL 27841, JL 27799 p.p. + *Matteria gracillima* + *Ulota rufula*).
Notes: new to Capitán Prat.

Cryphaeophilum molle (Dusén) M.Fleisch.
On rocks, rotten stumps, or epiphytic on *Amomyrtus luma* in *Nothofagus dombeyi* forests; alt. 50 m a.s.l.; very rare in the studied area.
Distribution: a Chilean endemic taxon (Kühnemann and Gonçalves Carralves 1976) previously reported from Cauquenes to Aisén provinces (Müller 2009a), so this record extends its southern distribution limit.
Exsiccata: Route 7 at fork Tortel/Yungay (JL 26485), Route 7 south of Cochrane km 90 (JL 27941, JL 27947A2 + *Lepyrodon patagonicus* + *Acrocladium auriculatum*).
Notes: new to Capitán Prat. Outside Chile this taxon has been only reported from Argentina on the road from Puerto Blest to Lago Frías (Río Negro Province), very close to the border with Chile (Calabrese and Matteri 1999).

Family Neckeraceae Schimp.

Neckera scabridens Müll.Hal.
Epiphitic or seldom on rocks in evergreen lowland forests; alt. 30-70 m a.s.l.; rare in the studied area.
Distribution: a Neotropical taxon, extending its distribution from the mountains of Costa Rica all the way down to southern Chile and Argentina, also in the Dominican Republic and SE Brazil (Sastre-de Jesús 1987), previously reported in Chile from Choapa to Aisén Province (Müller 2009a), so this record extends its southern distribution limit.
Exsiccata: Lago Quetru (JL 27834, JL 27101), Route 7 south of Cochrane km 90 (JL 27959 p.p. + *Syntrichia glacialis*, JL 27948).
Notes: new to Capitán Prat.

Porotrichum arbusculans (Müll.Hal.) Ochyra
=*Porothamnium arbusculans* (Müll.Hal.) M.Fleisch. ex Cardot & Broth.
On soil or rocks covered by litter in evergreen *Nothofagus dombeyi* and *Drimys-Weinmannia-Podocarpus* forests; alt. 5-100 m a.s.l.; not common in the studied area.

Distribution: a southern Andes endemic taxon (Robinson 1975), reported in Chile from Choapa to Magallanes provinces, as well as in Juan Fernandez Islands (Müller 2009a).
Exsiccata: Lago Quetru (JL 27851), Route 7 south of Cochrane km 90 (JL 27964), Fiordo Mitchell (JL 26544), Balsa Baker (JL 26699).
Notes: new to Capitán Prat.

Family Lepyrodontaceae Broth.

Lepyrodon hexastichus (Mont.) Wijk & Margad.
Epiphytic or less often on rocks inside evergreen *Nothofagus-Podocarpus-Drimys-Weinmannia* forests; alt. 20-100 m a.s.l.; not rare in the studied area.
Distribution: a southern South American endemic taxon (Allen 1999) growing in Chile between Curicó and Magallanes provinces, including the Juan Fernández Islands (Müller 2009a).
Exsiccata: Lago Quetru (JL 27779), Río Bravo (JL 26977), Balsa Baker (JL 26704), pasarela Lago Vargas (JL 26814), Fiordo Mitchell (JL 26571 p.p. + *Leptostomum menziesii*).
Notes: new to Capitán Prat.

Lepyrodon lagurus (Hook.) Mitt.
Epiphytic from relatively dry deciduous *Nothofagus* forests to more humid evergreen *Nothofagus-Pilgerodendron* forests, sometimes covering the rocks inside the forest; alt. 20-400 m a.s.l.; a very common taxon in the studied area.
Distribution: a southern South American endemic taxon extending into South Georgia (Allen 1999) growing in Chile between Talca Province and Cape Horn (Müller 2009a).
Exsiccata: Laguna Esmeralda (JL 27665, JL 27675), Lago Chacabuco (JL 27724), Tamango National Reserve (JL 26636), pasarela Lago Vargas (JL 26807, JL 26833), Route 7 south of Cochrane km 10 (JL 26385), Route 7 south of Cochrane km 26 (JL 26400), Lago Chico (ET 148e + *Lepyrodon patagonicus* + *Hygroamblystegium chilense* + *Bartramia mossmaniana* + *Hypnum skottsbergii* + *Sanionia uncinata*).
Notes: first reported to Capitán Prat by Skottsberg (1916) from NW Lago O'Higgins (CS 778).

Lepyrodon patagonicus (Cardot & Broth.) B.H.Allen
On rocks, rotten logs or epiphytic on *Azara* or *Amomyrtus* in *Nothofagus dombeyi* forests and *Drimys-Weinmannia-Podocarpus* forests, seldom on rocks in drier *Nothofagus antarctica* forests; alt. 20-340 m a.s.l.; common in the studied area.
Distribution: a southern South American endemic taxon (Allen 1999), reported in Chile from Valparaíso to Tierra del Fuego provinces, as well as in Juan Fernández Islands (Müller 2009a).
Exsiccata: Lago Chacabuco (JL 27717), Lago Quetru (JL 27843A), Route 7 south of Cochrane km 90 (JL 27939B, JL 27947A1 + *Cryphaeophilum molle* + *Acrocladium auriculatum*), Río Bravo (JL 27150), Fiordo Mitchell (JL 26577), Tamango National Reserve (JL 26642), Route 7 at fork Tortel/Yungay (JL 26470 p.p.+ *Calyptopogon mnioides*), Lago Chico (ET 148f + *Lepyrodon lagurus* + *Hygroamblystegium chilense* + *Bartramia mossmaniana* + *Hypnum skottsbergii* + *Sanionia uncinata*).
Notes: first reported to Capitán Prat by Allen (1999).

Lepyrodon tomentosus (Hook.) Mitt.
On rocks or epiphytic on *Nothofagus dombeyi or Podocarpus nubigena*, in evergreen forests; alt. 15-340 m a.s.l.; not common in the studied area.
Distribution: a Caribbean-South American taxon, extending its distribution from the mountains of Mexico all the way down to Tierra del Fuego, also in the Dominican Republic and SE Brazil (Allen 1999), in Chile reported from Talca Province to Cape Horn, as well as in Juan Fernández Islands

(Müller 2009a).
Exsiccata: Lago Chacabuco (JL 27721), road between Route 7 and Tortel (JL 26851), Caleta Tortel (JL 26916).
Notes: new to Capitán Prat.

Family Lembophyllaceae Broth.

Acrocladium auriculatum (Mont.) Mitt. (Figure 3C)
A forest species growing on soil, tree bases, rocks or fallen trees in a broad range of different forest types; alt. 20-300 m a.s.l.; common in the studied area.
Distribution: a southern South American endemic taxon (Tangney et al. 2010) growing in Chile between Ñuble Province and Cape Horn, including the Juan Fernández Islands (Müller 2009a).
Exsiccata: Laguna Esmeralda (JL 27661), Lago Quetru (JL 27783), Route 7 south of Cochrane (JL 27974, JL 27947A3 + *Lepyrodon patagonicus* + *Cryphaeophilum molle*), pasarela Lago vargas (JL 26806, AJ 347), Río Bravo (JL 26994), road to Tortel (JL 26432), Fiordo Mitchell (JL 26561), Lago Chacabuco (JL 27693C + *Syntrichia anderssoni* + *Polytrichum juniperinum* + *Cratoneuropsis relaxa* subsp. *minor*).
Notes: first reported to Capitán Prat by Skottsberg (1916) from NW Lago O'Higgins (CS 851).

Looseria orbiculata (Thér.) D.Quandt, Huttunen, Tangney & M.Stech
≡*Lembophyllum orbiculatum* (Thér.) Tangney
≡*Camptochaete orbiculata* (Thér.) H. Rob.
Epiphytic in *Nothofagus dombeyi-Embothrium coccineum-Amomyrtus luma* forest with dense *Fuchsia* and *Chusquea* understorey, on *Amomyrtus* and *Embothrium* bark; alt. 50 m a.s.l.; rare in the studied area.
Distribution: a southern South American endemic taxon, growing in Chile between Talca and Tierra del Fuego provinces (Müller 2009a).
Exsiccata: Route 7 south of Cochrane km 90 (JL 27939A, JL 27940, JL 27957).
Notes: new to Capitán Prat and to Aisén Region.

Rigodium adpressum Zomlefer
On the forest floor and on rocks, in *Nothofagus pumilio*, in *N. dombeyi*, or in mixed *N. dombeyi-Embothrium coccineum-Amomyrtus luma* forests; alt. 50-350 m a.s.l.; common in the studied area.
Distribution: an endemic species from southern South America (Zomlefer 1993), reported for Chile between Limarí Province and Cape Horn (Müller 2009a).
Exsiccata: Lago Chacabuco (JL 27716), Route 7 south of Cochrane km 90 (JL 27963, JL 27968), Villa O'Higgins (JL 27095), Laguna Esmeralda (JL 27679).
Notes: new to Capitán Prat.

Rigodium brachypodium (Müll.Hal.) Paris
On rocks inside mixed *Nothofagus dombeyi-Embothrium coccineum-Amomyrtus luma* forest; alt. 50 m a.s.l.; rare in the studied area.
Distribution: an endemic species from southern South America and the Juan Fernández Islands (Zomlefer 1993), reported for Chile between Limarí Province and Cape Horn (Müller 2009a).
Exsiccata: Route 7 south of Cochrane km 90 (JL 27925, JL 27971).
Notes: new to Capitán Prat.

Rigodium pseudothuidium Dusén
Epiphytic, on roots and tree bases, on tree stumps, or on litter on the floor of very wet *Weinmannia*

trichosperma-Nothofagus dombeyi-Drimys winteri-Podocarpus nubigena lowland rainforests; alt. 50-130 m a.s.l.; rare in the studied area.
Distribution: an endemic species from southern South America and the Juan Fernández Islands (Zomlefer 1993), reported for Chile between Choapa Province and Tierra del Fuego (Müller 2009a).
Exsiccata: Lago Quetru (JL 27801, JL 27107, JL 27125), Río Bravo (JL 26972, JL 27028), Fiordo Mitchell (JL 26580).
Notes: new to Capitán Prat.

Rigodium tamarix Müll.Hal.
On the forest floor, on tree bases, or on tree stumps in both mixed *Nothofagus dombeyi* forests and in *N. dombeyi-N. antarctica-Pilgerodendron uviferum* forests; alt. 15-30 m a.s.l.; rare in the studied area.
Distribution: an endemic species from central Chile and the Valdivian rainforests area (Zomlefer 1993, Müller 2009a), previously known in Chile between Limarí and Aisén provinces (Müller 2009a), so these records represent an extension in the southern distribution limit of the taxon.
Exsiccata: Lago Quetru (JL 27837), pasarela Lago Vargas (JL 26824), road between Route 7 and Tortel (JL 26848).
Notes: new to Capitán Prat.

Rigodium toxarion (Schwägr.) A.Jaeger var. ***toxarion***
Epiphytic in very wet *Weinmannia trichosperma-Nothofagus dombeyi-Podocarpus nubigena* forest; alt. 70 m a.s.l.; extremely rare in the studied area.
Distribution: a wide-spread taxon throughout South America, including the Juan Fernández Islands and also occurring in eastern Africa (Zomlefer 1993), previously known in Chile between Limarí and Aisén provinces (Müller 2009a), so this record represents an extension in the southern distribution limit of the taxon.
Exsiccata: Lago Quetru (JL 27106A).
Notes: new to Capitán Prat.

Rigodium toxarion var. ***robustum*** (Broth.) Zomlefer
On soil in the edge of *Nothofagus dombeyi-Embothrium coccineum-Amomyrtus luma* forest, on the side of the vehicular road; alt. 50 m a.s.l.; extremely rare in the studied area.
Distribution: this species has been considered an endemic of the Juan Fernández Islands (Zomlefer 1993), so this record represents the first report for the species in continental Chile and a great extension in its southern distribution range.
Exsiccata: Route 7 south of Cochrane km 90 (JL 27918).
Notes: new to Capitán Prat, to Aisén Region and to continental Chile. It differs from the typical variety of the species by the secondary branch leaves lanceolate to narrowly ovate, more than 2.25 times as long as wide, lacking the strong border and the prorulose and porose midlaminar cells characteristic of *R. brachypodium*.

Weymouthia cochlearifolia (Schwägr.) Dixon
Epiphytic in wet lowland forests of *Nothofagus-Drimys-Podocarpus-Amomyrtus*, frequently on branches of Myrtaceae species in the most humid parts of forests, often on river shores; alt. 15-130 m a.s.l.; not common in the studied area.
Distribution: a southern temperate species known from southern South America, New Zealand (Sainsbury 1955) and Australia (Streimann and Curnow 1989), growing in Chile between Limarí and Aisén provinces (Müller 2009a), so these records extend the southern limit of the distribution of the species.
Exsiccata: Lago Quetru (JL 27787A2 + *Hypnum cupressiforme* var. *filiforme* + *Daltonia gracilis* +

Eucamptodon perichaetialis), road to Tortel (JL 26853), Río Bravo (JL 27007, JL 27034).
Notes: new to Capitán Prat. Müller (2009a) checklist reports the taxon from Capitán Prat Province, but any of the references he cites refer to material collected in the Province.

Weymouthia mollis (Hedw.) Broth.
Epiphytic in wet lowland forests of *Nothofagus-Drimys-Podocarpus-Amomyrtus-Weinmannia*, on living or dead branches and twigs of several Myrtaceae species, *Podocarpus*, *Fuchsia*, etc.; alt. 30-130 m a.s.l.; common in the studied area.
Distribution: a southern temperate species known from sothern South America, New Zealand (Sainsbury 1955) and Australia (Streimann and Curnow 1989), reported in Chile between Limarí and Magallanes provinces, as well as in Juan Fernández (Müller 2009a).
Exsiccata: Lago Quetru (JL 27788A + *Eucamptodon perichaetialis* + *Hypnum cupressiforme* var. *filiforme* + *Zygodon pentastichum* + *Z. reinwardtii*, JL 27832), road between Route 7 and Tortel (JL 27900), Río Bravo (JL 26978, JL 27030B + *Chorisodontium dicranellatum* + *Matteria papillosula* + *Ulota pusilla*), Route 7 at Tortel/Yungay fork (JL 26478), Fiordo Mitchell (JL 26570), Lago Vargas (AJ 2).
Notes: new to Capitán Prat.
